# A Scalable Robust Microporous Al‐MOF for Post‐Combustion Carbon Capture

**DOI:** 10.1002/advs.202401070

**Published:** 2024-03-25

**Authors:** Bingbing Chen, Dong Fan, Rosana V. Pinto, Iurii Dovgaliuk, Shyamapada Nandi, Debanjan Chakraborty, Nuria García‐Moncada, Alexandre Vimont, Charles J. McMonagle, Marta Bordonhos, Abeer Al Mohtar, Ieuan Cornu, Pierre Florian, Nicolas Heymans, Marco Daturi, Guy De Weireld, Moisés Pinto, Farid Nouar, Guillaume Maurin, Georges Mouchaham, Christian Serre

**Affiliations:** ^1^ Institut des Matériaux Poreux de Paris Ecole Normale Supérieure ESPCI Paris CNRS PSL University Paris 75005 France; ^2^ ICGM Univ. Montpellier CNRS ENSCM Montpellier 34293 France; ^3^ Service de Thermodynamique et de Physique Mathématique Faculté Polytechnique Université de Mons Mons 7000 Belgium; ^4^ Normandie Université ENSICAEN UNICAEN CNRS Laboratoire Catalyse et Spectrochimie Caen 14000 France; ^5^ Swiss–Norwegian Beamlines European Synchrotron Radiation Facility 71 Avenue des Martyrs Grenoble 38000 France; ^6^ CERENA Departamento de Engenharia Química Instituto Superior Técnico Universidade de Lisboa Lisboa 1049‐001 Portugal; ^7^ CICECO‐ Aveiro Institute of Materials Department of Chemistry University of Aveiro Campus Universitário de Santiago Aveiro 3810‐193 Portugal; ^8^ Centre National de la Recherche Scientifique (CNRS) UPR3079 CEMHTI Université d'Orléans 1D Av. Recherche Scientifique, CEDEX 2 Orléans 45071 France

**Keywords:** CCS, CO_2_, MOFs, scale‐up, shaping, techno‐economic analysis

## Abstract

Herein, a robust microporous aluminum tetracarboxylate framework, MIL‐120(Al)‐AP, (MIL, AP: Institute Lavoisier and Ambient Pressure synthesis, respectively) is reported, which exhibits high CO_2_ uptake (1.9 mmol g^−1^ at 0.1 bar, 298 K). In situ Synchrotron X‐ray diffraction measurements together with Monte Carlo simulations reveal that this structure offers a favorable CO_2_ capture configuration with the pores being decorated with a high density of *µ*_2_‐OH groups and accessible aromatic rings. Meanwhile, based on calculations and experimental evidence, moderate host‐guest interactions *Q*_st_ (CO_2_) value of MIL‐120(Al)‐AP (−40 kJ mol^−1^) is deduced, suggesting a relatively low energy penalty for full regeneration. Moreover, an environmentally friendly ambient pressure green route, relying on inexpensive raw materials, is developed to prepare MIL‐120(Al)‐AP at the kilogram scale with a high yield while the Metal‐ Organic Framework (MOF) is further shaped with inorganic binders as millimeter‐sized mechanically stable beads. First evidences of its efficient CO_2_/N_2_ separation ability are validated by breakthrough experiments while operando IR experiments indicate a kinetically favorable CO_2_ adsorption over water. Finally, a techno‐economic analysis gives an estimated production cost of ≈ 13 $ kg^−1^, significantly lower than for other benchmark MOFs. These advancements make MIL‐120(Al)‐AP an excellent candidate as an adsorbent for industrial‐scale CO_2_ capture processes.

## Graphical abstract

A robust ultra‐microporous Al‐MOF is presented as a promising candidate for point source CO_2_ capture, owing to 1‐D channels decorated with a high density of *µ*_2_‐OH groups and accessible aromatic rings. The MOF is produced at the kg‐scale in water under green conditions, while a techno‐economic analysis gives an estimated production cost significantly lower than other benchmark MOFs.

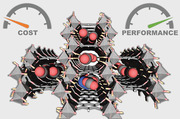

## 1 Introduction

Carbon capture, utilization, and storage (CCUS) is envisioned to significantly tackle increasing levels of atmospheric CO_2_ achieving the prevention of global warming.^[^ ^1^ ^]^ Among the current potential carbon capture technologies, post‐combustion capture of CO_2_ from flue gases emitted from power plants and carbon‐intensive industries such as steel or cement production is considered a feasible and economically viable process, as it might be potentially retrofitted to the existing fleet of coal‐fired power stations or industrial plants.^[^ ^2^ ^]^ So far, aqueous amine solution through a combination of chemical and physical absorption affinity with CO_2_ molecule is the most applicable and mature technology for CO_2_ capture.^[^ ^3^ ^]^ However, it raises important environmental issues as well as a high energy penalty, on average 15–20% but, for instance, up to 80% of total thermal energy consumption for coal‐fired power plants, in the worst case.^[^ ^4^ ^]^ Physisorptive CO_2_ capture as an efficient low‐heat technology is thus expected to provide much lower energy consumption for regeneration with respect to rich amine solutions (≈ 90–160 kJ mol^−1^).^[^ ^5^ ^]^

Porous solid adsorbents, such as zeolites, are potential candidates for CCUS due to their excellent working capacity and selectivity for CO_2_ over other gases present in the industrial gas stream.^[^ ^6^ ^]^ However, despite their relatively low production cost, for the most common ones, and high thermal/chemical stability, the detrimental competitive adsorption of water molecules significantly decreases their CO_2_ working capacity and selectivity as well as requires very high‐temperature regeneration to desorb water.^[^ ^6b^ ^]^ Alternatively, amine‐functionalized porous materials or porous organic materials are suitable alternative candidates.^[^ ^7^ ^]^ For instance, Long et al. grafted tetraamine chains on the large pore Mg_2_(dobpdc) (dobpdc = 4,4′‐dioxidobiphenyl‐3,3′‐dicarboxylate) Metal– Organic Framework (MOF) through post‐synthesis modification, improving the efficiency in CO_2_ capture under harsh conditions relevant to natural gas flue emissions.^[^ ^8^ ^]^ Most notably, the relatively higher thermal stability of the tetraamine‐functionalized framework was exploited, enabling a regeneration through direct contact with steam, resulting in a significant energy saving over the conventional methods. Still, the relatively high energy penalty for the regeneration, long‐term stability issues, lack of sustainability, and/or challenges in the up‐scaling raise important limitations for the use of these classes of solids for real industrial separation processes.^[^ ^9^ ^]^ Robust MOFs, generally built either from high valence metal oxoclusters/chains and poly‐carboxylates or phosphonates, or alternatively metal(II) poly‐azolates, are also candidates for CO_2_ capture. Their high customizability enabling a precise tuning and functionalization of the pore structure, has been intensively investigated for selective gas separation applications.^[^ ^10^ ^]^ Indeed, one can either for instance tune their hydrophobic character to mitigate the negative effect of water, and/or modify them with exogenous chemical species for enhanced performances. Recently, CALF‐20, a microporous robust Zn_2_(1,2,4‐triazolate)_2_(oxalate) material (CALF stands for Calgary Frameworks) has emerged as an alternative benchmark CO_2_ sorbent to standard zeolite 13X with physisorptive mechanism for the CO_2_ capture from cement flue gas or high CO_2_ concentration in industrial flue gases.^[^ ^11^ ^]^ Due to its relatively moderate isosteric enthalpy of adsorption (≈ ‐40 kJ mol^−1^), excellent CO_2_ capacity in post‐combustion conditions (2.47 mmol g^−1^ at 0.1 bar and 298 K), good CO_2_/N_2_ selectivity (230 for a 10CO_2_:90N_2_ mixture), moderate hydrophilicity and long‐term stability under real conditions, it was exploited, once coated on a rotary bed, for the capture of CO_2_ in presence of moisture up to ≈ 25–30% RH (when structured with polysulfone‐based binder). This MOF was also proven to be scalable at the ton‐scale using an ambient pressure optimized protocol leading to an estimated production cost of ≈ 25–30 $ kg^−1^. Its production at the hundred tons scale is now on the way prior to being integrated into larger‐scale flue gas capture units in the cement industry. Fluorinated MOFs, such as SIFSIX‐ and TIFSIX‐based MOFs or NbOFFIVE‐1‐Ni, are also considered potential candidates for post‐combustion capture due to their outstanding CO_2_ adsorption capacity and CO_2_/N_2_ selectivity.^[^ ^12^ ^]^ However, their long‐term hydrolytic stability is in most cases limited, while their large‐scale production is still hampered by strong safety (and cost) issues, mainly due to the potential formation of HF. From the material cost point of view, aluminum‐based MOFs (Al‐MOFs), particularly if relying on widely available low‐cost commercial polycarboxylic acids, and inexpensive Al metal sources, whenever they can endow comparable CO_2_ adsorption performance with CALF‐20, stand as very promising candidates for CO_2_ capture. One could highlight additional potential robust MOF candidates, such as A520 or Al fumarate,^[^ ^13^ ^]^ MOF‐303,^[^ ^14^ ^]^ MIL‐160(Al),^[^ ^15^ ^]^ (MIL stands for Materials from Institute Lavoisier), USTA‐16,^[^ ^16^ ^]^ (USTA stands for University of Texas at San Antonio), MUF‐16,^[^ ^17^ ^]^ (MUF stands for Massey University Framework), which can be obtained at multi‐kg scale via facile, green and one‐pot synthesis procedure and simply manufactured as structured adsorbents, e.g., pellets, beads, monoliths, and fibers.^[^ ^18^ ^]^ However, their CO_2_ capture performances are usually significantly lower compared to the benchmark CALF‐20.

In this regard, we selected the robust microporous aluminum 1,2,4,5‐benzene tetracarboxylate framework MIL‐120(Al), due to its excellent CO_2_ capture efficiency at relatively low pressure (1.9 mmol g^−1^ at 0.1 bar at 298 K), comparable to the best amine‐free benchmark CO_2_ adsorbents, as well as its potentially cheap production cost. Indeed, the high affinity of this MOF for CO_2_ was initially (briefly) described by Loiseau et al. more than a decade ago.^[^ ^19^ ^]^ Thus, due to its promising performances, we have further explored this MOF through an in‐depth joint experimental/computational study to shed light on the key features driving its remarkable CO_2_ adsorption performances. Monte Carlo simulations, confirmed by in situ IR spectroscopy, in situ PXRD, and low‐pressure adsorption experiments, showed that the high density of *µ*_2_‐OH groups, as well as the accessible aromatic rings decorating the channels, play a pivotal role in the CO_2_ adsorption at very low pressure, with a relatively moderate isosteric enthalpy of adsorption (*Q*_st_) (−40 kJ mol^−1^) (comparable to benchmark CO_2_ physisorbents). This MOF also possesses high CO_2_/N_2_ selectivity (≈ 90) for binary gas mixtures 15CO_2_:85N_2_ and 5CO_2_:95N_2_ at 298 K and 1 bar, as estimated based on the Ideal Adsorbed Solution Theory (IAST), further confirmed by coadsorption experiments. Indeed, all these features confer MIL‐120(Al) a high potential for CO_2_ capture from flue gases. An in situ synchrotron radiation Powder X‐Ray Diffraction (SRPD) experiment was also carried out to better understand the structural behavior of these microporous frameworks and revealed that unlike MIL‐120(Al)‐HP, MIL‐120(Al)‐AP exhibits a phase transition, from monoclinic to triclinic, during the removal of the guest (water) molecules. Furthermore, as this material was initially made under hydrothermal conditions, an alternative eco‐friendly ambient‐pressure (AP) synthesis procedure was developed relying on cost‐effective chemicals suitable for the easy scale‐up of MIL‐120(Al)‐AP, while keeping an excellent crystallinity and porosity, comparable to the high‐pressure sample, denoted MIL‐120(Al)‐HP (HP stands for High Pressure). Breakthrough curve measurements confirmed the efficient adsorptive separation of CO_2_/N_2_ using MIL‐120(Al)‐AP. Besides, as MIL‐120 is slightly more hydrophilic than CALF‐20, an advanced operando IR study was carried out and evidenced a faster sorption kinetics for CO_2_ versus H_2_O, suggesting the potential of MIL‐120(Al) for CO_2_/N_2_ separation in the presence of water when an adequate fast cycling process design is applied. Finally, a techno‐economic analysis was performed with the aim of assessing the production costs of this material, considering a large production scale of 1 kton per year, which would cover only <5% of the needs of existing cement plants worldwide and is much lower than the market scale of performant adsorbents,^[^ ^20^ ^]^ revealing a production cost of 13 $ kg^−1^.

## 2 Results and Discussion

### 2.1 Structure, Synthesis Optimization and Adsorption Performances

**Figure 1:**
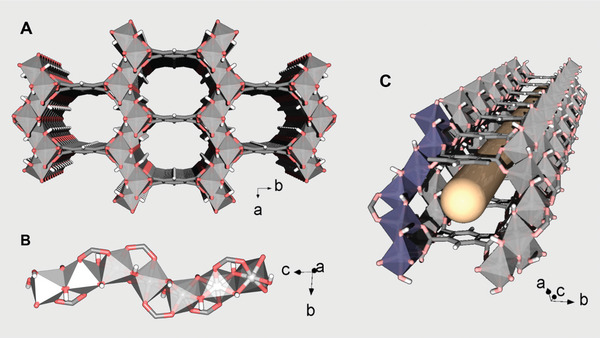
Crystal structure of MIL‐120(Al). A) General view along [0 0 1] highlighting the MOF narrow channels (water molecules were omitted for clarity). B) Constitutive Al hydroxo‐chains built of *trans‐cis* edge sharing Al(OH)_4_O_2_ octahedra. C) Representation of one channel emphasizing its highly confined environment (represented by the yellow tube) due to dense network of *µ*_2_‐OH and stacked phenyl rings of BTeC. Color code: Al(OH)_4_O_2_, gray polyhedra; C, gray; O, light red; H, white. In C), one of the chains is highlighted using purple polyhedra.

The crystal structure of MIL‐120(Al) or Al_4_(OH)_8_(C_10_O_8_H_2_) *x*H_2_O (*x* = 4.8–5) was reported more than a decade ago by Loiseau et al., the MOF being prepared through a hydrothermal route.^[^ ^21^ ^]^ Its inorganic sub‐unit is composed of Al(OH)_4_O_2_ octahedra, with oxygen atoms from four hydroxyl groups and two different carboxylate groups belonging to two neighboring organic ligands (pyromellitate or 1,2,4,5‐benzene‐tetracarboxylate, abbreviated BTeC^4−^). This results in infinite Al hydroxo‐chains, rather uncommon in Al‐MOFs, built of *trans‐cis* edge sharing Al(OH)_4_O_2_ octahedra linked via double *µ*_2_‐OH bridges. These chains are linked together through the four carboxylates of the BTeC^4−^ (hereafter BTeC), thereby generating a 3D framework that delimits 1D narrow channels running along [0 0 1] direction with a free section dimension of ≈ 5.4 Å × 4.7 Å (**Figure** **1**A). Therefore, in comparison with other corner‐sharing modes commonly present in most Al‐MOFs (Figure 1B), MIL‐120(Al) provides not only two‐fold denser polar hydroxyl groups, which could be beneficial for enhanced CO_2_ interactions with the framework, as well as four times less organic spacer per metal center, which might be highly beneficial to reduce the cost production at large scale as it is admitted that the Al‐MOF production cost is highly dependent on the organic ligand price.^[^ ^20^ ^]^ We also considered that this high L/M ratio as well as the four connectivity of the ligand, could lead to enhanced chemical and thermal stabilities; for instance, it is well known that the Fe tetracarboxylate MIL‐127(Fe) is slightly more chemically stable than the iron trimesate MIL‐100(Fe).^[^ ^22^ ^]^ Another feature that has motivated us to explore the capabilities of this MOF for CO_2_ capture is the parallel arrangement of the aromatic groups of BTeC with a distance ranging between 6.5 – 7.0 Å (out of van der Waals radii) (Figure 1C), recently predicted by Smit et al. as ideal adsorbaphore for CO_2_.^[^ ^23^ ^]^

**Figure 2:**
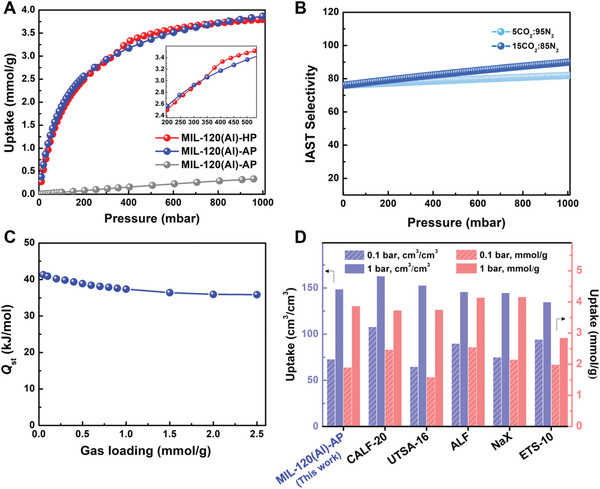
CO_2_ adsorption performances of MIL‐120(Al)‐HP and MIL‐120(Al)‐AP at 298 K. A) CO_2_ (in red and blue, respectively) and N_2_ (in gray) adsorption isotherms at 298 K. Enlargement on the step region is given in the inset. B) IAST selectivity at different compositions for MIL‐120(Al)‐AP using the Langmuir model. C) Isosteric enthalpy of adsorption versus CO_2_ uptake for MIL‐120(Al)‐AP. D) Comparison of volumetric and gravimetric CO_2_ uptakes at 0.1, 1 bar, at 298 K between MIL‐120(Al)‐AP and benchmark adsorbents including MOFs and zeolites. The volumetric uptake was calculated using the crystallographic density.

The high‐pressure CO_2_ adsorption isotherm reported in 2009 showed already promising performances in terms of CO_2_ uptake while Stylaniou et al., during the course of our study,^[^ ^24^ ^]^ reported new insights over the CO_2_ capture properties under post‐combustion conditions with however details about the mechanism in play.^[^ ^25^ ^]^ In both cases the authors also relied exclusively on the hydrothermal sample, limiting the chances of synthesis scalability at a reasonable cost. Herein, for the sake of understanding, we first prepared MIL‐120(Al)‐HP following the reported hydrothermal synthesis, and collected the CO_2_ adsorption data at 298 K. Indeed, the obtained isotherm confirmed, as expected, the promising CO_2_ capacity at low pressure (**Figure** **2**A). Motivated by these attractive adsorption performances, it was of crucial importance to obtain this MOF following a simpler, safer, scalable, and cost‐effective synthesis protocol in view of the large‐scale production and further exploitation in real separation processes. Therefore, we carried out a thorough systematic experimental study to set up an optimized green synthesis at ambient pressure. Taking into account that the H_4_BTeC – Al(III) system is quite complex to optimize as different MOFs such as MIL‐118(Al) and MIL‐121(Al) can easily be formed once exploring this system.^[^ ^26^ ^]^

While both are based on more common infinite chains of *trans*‐connected corner‐shared aluminum‐centered octahedra, similar to that of MIL‐53(Al)’s, in the case of MIL‐121(Al), the ligand only involves two carboxylate groups to bridge the chains, leaving the remaining two others free, pointing toward the center of the channels. If the ratio Al/H_4_BTeC/H_2_O has a direct influence on the crystallization of the different phases, the initial pH of the reaction mixture was shown to be of major importance. Indeed, it was demonstrated, in the previously studied hydrothermal conditions, that the occurrence of MIL‐120(Al), showcasing the highest density of *µ*_2_‐OH and up to 4‐fold higher Al/BTeC ratio, is driven by a basic pH > 10 (≈ 12 vs ≈ 2 and 1.4 for MIL‐118(Al) and MIL‐121(Al), respectively). These observations were capital to optimize the synthesis conditions under ambient pressure. Thus, we selected less acidic Al‐precursors to promote the formation of edge‐sharing Al‐chains, while we varied the Al/H_4_BTeC ratio, the concentration, and additives to control the pH/solubility. More details are provided in Table S1 (Supporting Information). The use of Al(OH)_3_ systematically led to the formation of the MIL‐121(Al) phase. Despite the basic nature of this precursor, its limited solubility in water resulted in acidic pH conditions attributed to the gradual dissolution of H_4_BTeC occurring at a faster rate. To reach a higher solubility of the Al precursor, we first explored the Al(OH)(OAc)_2_ (OAc stands for acetate) which has a weakly basic character and a relatively good solubility in water, while the released acetate groups can also contribute to control the crystallization process (as modulators). Interestingly, all the trials with different Al/H_4_BTeC ratios (ranging from 2 to 8) yielded MIL‐120(Al) phase as a sole, pure, and well‐crystallized structure, whereas the pH at the end of the reaction was neutral. It should, however, be noted that monitoring the starting pH can be misleading because of the higher solubility of H_4_BTeC compared to Al(OH)(OAc)_2_ at room temperature (RT). These first optimizations were carried out in 15–30 mL of water leading to ≈ 1 g product. We then opted to reproduce similar synthetic conditions at a slightly larger scale reacting stoichiometric precursor amounts (4Al/1BTeC: 80 mmol/20 mmol) in 300 mL in water to yield ≈ 10 g of MIL‐120(Al)‐AP, which was further thoroughly washed in warm water whose high purity was verified by a large set of complementary characterizations including PXRD, Fourier‐Transform Infrared (FT‐IR) spectroscopy, Thermal‐Gravimetric Analysis (TGA), N_2_ adsorption at 77 K, CO_2_, and water adsorption isotherms at 298 K (see supplementary information for details, Figure S1, Supporting Information).

Indeed, the PXRD pattern of the synthesized MIL‐120(Al)‐AP sample is in excellent agreement with the theoretical one calculated from the crystal structure (Figure S1A, Supporting Information), with however a slightly broader profile compared to that of MIL‐120(Al)‐HP, mainly due to the difference of particle sizes (i.e., few microns vs submicron, for the HP and AP syntheses, respectively). FT‐IR spectra and TGA analyses (Figure S1B,C, Supporting Information) also showed both a very good agreement for the samples obtained from hydrothermal and ambient pressure methods, although the steps of the TGA curves were slightly less well defined in the case of MIL‐120(Al)‐AP and a total decomposition occurring ≈ 50 °C lower compared to MIL‐120(Al)‐HP. Unexpectedly, the N_2_ isotherms measured at 77 K (Figure S1D, Supporting Information) showed a significant difference in uptakes, with an almost two‐fold higher compared to the reported values, in favor of MIL‐120(Al)‐AP, with a calculated BET surface area for the latter of ≈ 590 m^2^ g^−1^. This could be attributed to a potential shrinkage/flexibility of the structure at 77 K more or less pronounced depending on the particle size. However, in our case, the experimental water adsorption isotherms of MIL‐120(Al)‐HP and MIL‐120(Al)‐AP at 298 K (Figure S1E, Supporting Information) gave similar profiles and uptakes, noting here that the high density of *µ*_2_‐OH groups in this structure confers to MIL‐120(Al) a rather hydrophilic character. Furthermore, single‐component adsorption isotherms of CO_2_ and N_2_ were carried out at 298 K. As shown in Figure 2A and Figure S1F (Supporting Information), the CO_2_ uptake in MIL‐120(Al)‐AP was 1.90 mmol g^−1^ (73.1 cm^3^ cm^−3^, based on the crystallographic density, 1.57 cm^3^ g^−1^, as calculated for the reported crystal structure,^[^ ^21^ ^]^ without taking into account any guest molecule) and 3.87 mmol g^−1^ (148.8 cm^3^ cm^−3^, STP) at 0.1 and 1 bar, respectively, which is comparable, although slightly higher, to that of the hydrothermally synthesized sample and the very recent reported results reported by Stylaniou et al, suggesting a better activation or purity in our case.^[^ ^25^ ^]^ Noteworthy, the occurrence of a small step at 0.35 bar of CO_2_ occurs only in the case of MIL‐120(Al)‐HP. Additionally, under the same measurement conditions, MIL‐120(Al)‐AP adsorbed a much lower amount of N_2_ (0.32 mmol g^−1^ at 0.9 bar). Estimation of the CO_2_/N_2_ selectivity was performed by applying the Ideal Adsorbed Solution Theory (IAST) model (see Table S2, Supporting Information) and revealed for MIL‐120(Al)‐AP an excellent selectivity, >80 when calculated for binary gas mixtures 15CO_2_:85N_2_ and 5CO_2_:95N_2_ at 298 K, 1 bar (Figure 2B). This confirms the high potential of MIL‐120(Al)‐AP for the separation of CO_2_ from flue gases (5‐30% CO_2_) such as those emitted from industrial plants or other sources. Meanwhile, coverage‐dependence of *Q*_st_ was determined for CO_2_ in MIL‐120(Al)‐AP by applying the Clausius‐Clapeyron equation to single component adsorption isotherms collected at 298, 308, and 318 K (Figure S2, Supporting Information). The experimental *Q*_st_ of CO_2_ at near‐zero coverage was found to be −41 kJ mol^−1^ (Figure 2C), close to the value reported previously for benchmark CO_2_ adsorbent CALF‐20 (≈ −40 kJ mol^−1^). Interestingly, this *Q*_st_ shows only a minor decrease (≈ 5 kJ mol^−1^) with the increase of CO_2_ loading. The overall results strongly suggest the good CO_2_ capture performance of this MOF due to its potential high CO_2_ working capacity, and high CO_2_/N_2_ selectivity, while still exhibiting a relatively low energy for regeneration. As highlighted in Figure 2D and Figure S3 (Supporting Information), it is noteworthy to mention that at 0.1 bar and 1 bar at 298 K, the CO_2_ uptake of MIL‐120(Al)‐AP is comparable to those of benchmark CO_2_ adsorbents (if not higher in some cases), such as MOF‐based adsorbents (CALF‐20,^[^ ^11^ ^]^ Mg‐MOF‐74,^[^ ^27^ ^]^ UTSA‐16,^[^ ^28^ ^]^ ALF (Al‐Formate MOF),^[^ ^29^ ^]^ SIFSIX‐3‐Cu,^[^ ^30^ ^]^ mmen‐Mg_2_(dobpdc),^[^ ^8^ ^]^ SIFSIX‐3‐Zn,^[^ ^12c^ ^]^ Ni‐MOF‐74^[^ ^31^ ^]^) as well as several cationic zeolites (NaX,^[^ ^32^ ^]^ ETS‐4/10,^[^ ^33^ ^]^ ETS stands for Engelhard Titanium Silicates). Besides, some of the state‐of‐the‐art PSA sorbent candidates with a potential for commercialization have been listed in Table S3 (Supporting Information).

In fact, if the performances are crucial features for a given application, other aspects including the material's cost, sustainability, chemical and thermal stability, and processability, are also important criteria to take into account, in particular when it potentially implies a very large‐scale application such as CO_2_ capture requiring a huge amount of adsorbents, i.e., more than a few hundred tons per plant.^[^ ^34^ ^]^ TGA coupled with variable‐temperature PXRD analysis (Figure S4, Supporting Information), confirmed that MIL‐120(Al) exhibits excellent thermal stability (up to 400 °C), although some small variations in diffracted Bragg peaks could be observed starting from 100 °C, very likely due to slight structural flexibility induced by guest removal and/or some bond rearrangement (e.g., OH groups, etc.). Moreover, MIL‐120(Al)‐AP also showed exceptional hydrolytic stability, withstanding boiling water for (at least) 10 days as confirmed by the well‐preserved crystallinity, chemical composition, morphology, thermal stability, and CO_2_ capacity, as per the provided PXRD and CO_2_ adsorption data, respectively (Figure S5, Supporting Information). This is, to our knowledge, the most hydrothermally stable Al‐carboxylate‐based MOF reported to date. Consequently, this encouraged us to further characterize deeply the CO_2_ adsorption behavior of MIL‐120(Al)‐AP.

### 2.2 In Situ Synchrotron Radiation Powder Diffraction (SRPD) Studies

**Figure 3:**
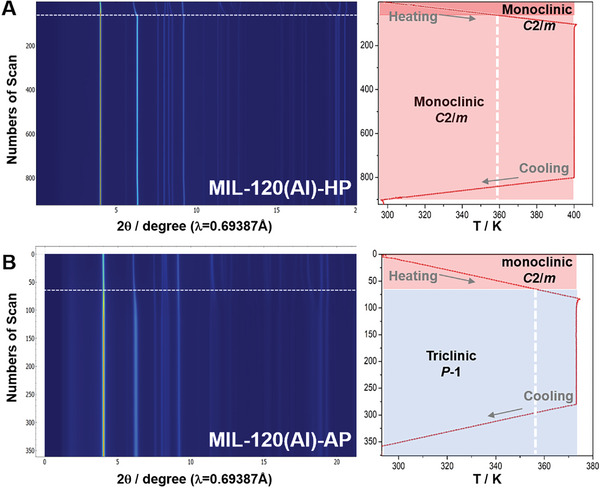
Variable temperature synchrotron SRPD data. Measurements performed under dynamic vacuum (activation step) for A) MIL‐120(Al)‐HP, showing no phase transition, while remaining monoclinic; and B) MIL‐120(Al)‐AP revealing phase transition, at ≈ 357 K, from monoclinic to triclinic phase. Heating rate is equal to 6 K min^−1^, up to 373 K in (A) and 400 K in (B).

In comparison with MIL‐120(Al)‐HP, the CO_2_ adsorption isotherms of MIL‐120(Al)‐AP do not exhibit any step behavior during CO_2_ adsorption, and this is regardless of the purification quality and the activation temperature. In an attempt to better understand this intriguing behavior and to shed light on the preferential interaction sites between CO_2_ and the porous framework, pressure‐ and temperature‐variable in situ SRPD collected at BM01 from the Swiss‐Norwegian beamlines (SNBL) at the European Synchrotron Radiation Facility (ESRF, Grenoble in France) for both samples were carried out, by monitoring the SRPD patterns starting from the activation processes (heating up to 400 K under dynamic secondary vacuum) and during all over the CO_2_ loading up to 4000 mbar. Variable temperature SRPD data collected directly during activation steps are depicted in **Figure** **3**.

In the case of MIL‐120(Al)‐HP, the analysis of the SRPD patterns evolution during the heating process revealed that the related crystal systems and space groups remained unchanged (monoclinic, *C*2/*m* (*n°*15)), despite minor variations in cell parameters, particularly an increase of the *β* angle and *c* parameter (Figure 3A; Figure S6, Supporting Information). Unexpectedly, MIL‐120(Al)‐AP displayed a different behavior associated with a phase transition at ≈350 K from monoclinic (*C*2/*m*) to triclinic (*P*‐1 (*n°*2)) phase (Figure 3B; Figures S11 and S12, Supporting Information). The differences in particle sizes (micrometer vs submicronic for HP and AP phases, respectively) could explain the difference in behavior.^[^ ^35^ ^]^ According to the refinement of crystal structures with the Rietveld method in Fullprof,^[^ ^36^ ^]^ for samples having been heated up to 400 K (for MIL‐120(Al)‐HP) or 373 K (for MIL‐120(Al)‐AP) and cooled to 298 K, shown in both cases, that water molecules have been removed from the pores (see Figures S8 and S13 Supporting Information). A detailed description of the SRPD is included in the Supporting Information.

**Figure 4:**
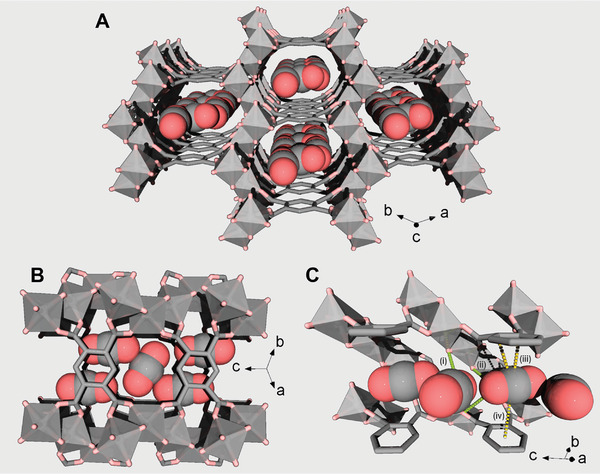
Crystal structure of MIL‐120(Al)‐HP cooled down to 200 K and loaded with 1 bar of CO_2_. A) General view along [0 0 1]. B) Top view of a part of one channel, and C) a cut through a channel showing the arrays of alternated CO_2_ molecules and their interactions with the frameworks throughout *µ*_2_‐OH (represented as green dashed lines) and phenyl groups (yellow dashed lines) of the BTeC, with the respective distances i): 3.6Å; ii): 3.3Å; iii): 3.6Å; iv): 3.7Å). Color code: Al(OH)_4_O_2_, gray polyhedra; C, gray; O, light red. H‐atoms were not localized.

The steady adsorption loadings of CO_2_ at 298 and 200 K were further investigated for both samples. In particular, for a deeper understanding of the positions of CO_2_ molecules and their interactions with the framework, the loading under different pressures up to 4 bar of CO_2_ at 298 K was investigated for MIL‐120(Al)‐HP. The positions of the CO_2_ molecules were found for this high pressure by the direct space methods in FOX^[^ ^37^ ^]^ and were refined using the Rietveld method in Fullprof.^[^ ^36^ ^]^ The MIL‐120(Al)‐HP sample shows a phase transition from *C*2/*m* (*n°*15) to *P*‐1 (*n°*2), contrary to the MIL‐120(Al)‐AP, which maintains the same space group up to 1.5 bar in this study. The crystal structure refinement of MIL‐120(Al)‐HP suggests fully occupied CO_2_ molecules located along [0 0 1] channels, showing the van der Waals O···O guest‐host interatomic distances of 3.37 and 3.46 Å as well as intermolecular O···O separations of 2.95 Å. In order to minimize the motion effects and to get higher gas loading, the emptied (dynamic vacuum) sample was cooled down to 200 K and CO_2_ was loaded stepwise again up to 1 bar. The *P‐*1 crystal structure remained for MIL‐120(Al)‐HP at these conditions. The previous CO_2_ molecules kept their location along the [0 0 1] channels (with a slight shift), however, one more independent molecule is expected in the middle of the pore (with ½ of occupancy), laying between their two arrays, see (**Figure** **4**A). Each array is composed of alternated CO_2_ molecules with occupancies of ≈ 0.82 (Figure 4B). As depicted in Figure 4C, a closer look at the interaction of these molecules with the frameworks showed i) that CO_2_ mainly interacted with the *µ*_2_‐OH groups (*d*(O···O) ranging from ≈ 3.03 Å to 3.34 Å), while ii) the second CO_2_ interacts with two *µ*_2_‐OH between the opposite Al‐oxo chains with relatively weaker interactions (d(O···O) ≈ 3.43 and 4.01 Å); and from another side with the aromatic phenyl rings that belong to two stacked BTeC (delimiting the channels) via dispersion and electrostatic interactions (very likely between the carbon of the aromatic ring and the central carbon atom of CO_2_, in addition to further interactions between one electropositive aromatic H atom (not localized in this structure) and one electronegative O atom of CO_2_).^[^ ^38^ ^]^ As mentioned, the MIL‐120(Al)‐AP sample remains without any significant changes at 298 K (at least up to 1.5 bar), however at 200 K and 1 bar of CO_2_, it adopts monoclinic *C*2/*m* crystal structure with a similar to MIL‐120(Al)‐HP two independent CO_2_ molecules. The two arrays of CO_2_ molecules have a similar to MIL‐120(Al)‐HP (≈ 0.82 occupancies) and are located in parallel to each other in front of two *µ*_2_‐OH (d(O···O) 3.06 Å). They have a comparable intermolecular distance of d(C···C) 3.05 Å. The second CO_2_ molecule fully occupies the center of the pore and has a bit longer connectivity with four *µ*_2_‐OH (d(O···O) 3.26 Å) and shorter d(O···O) 2.60 Å intermolecular contacts with other CO_2_ molecules. More details about the phase transition analysis are included in Figures S6–S14 (Supporting Information).

### 2.3 Molecular Simulations

**Figure 5:**
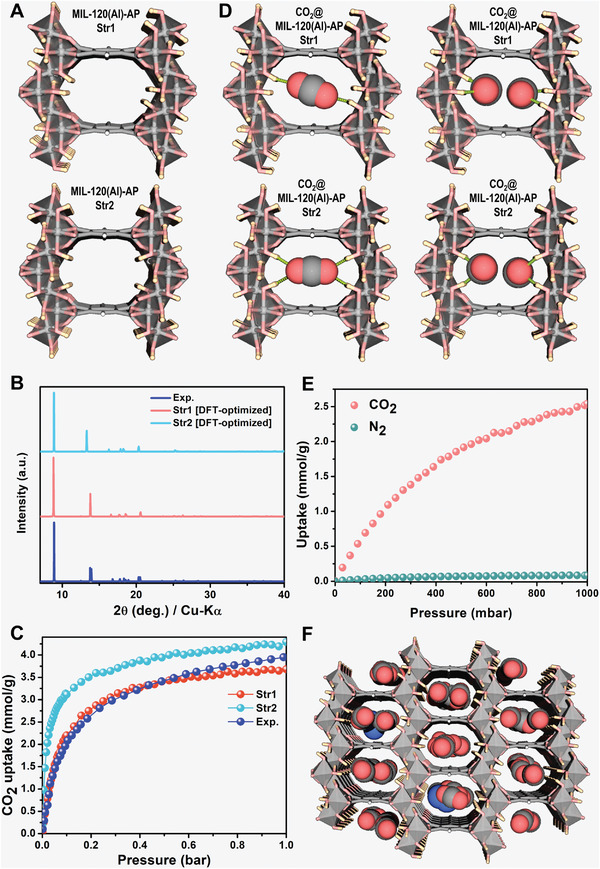
Molecular simulations of MIL‐120(Al)‐AP structure and adsorption properties. A) Most stable DFT‐optimized structure of MIL‐120(Al). The unit cell parameter is not shown to highlight the different orientations of *µ*_2_‐OH group in the channel. The most stable Str1 and Str2 structures correspond to distinct orientations of µ_2_‐OH groups [details in Supporting Information]. B) Calculated X‐ray diffraction patterns for the two most stable DFT‐optimized MIL‐120(Al)‐APs and the corresponding experimental data. C) GCMC‐simulated and experimental single‐component CO_2_ adsorption isotherms at 298 K. D) Illustrations of the DFT‐optimized CO_2_‐loaded MIL‐120(Al)‐AP‐Str1 and MIL‐120(Al)‐AP‐Str2 structures with different CO_2_ loading (one [top] and two [bottom] CO_2_ molecules per unit‐cell). The dashed line represents the interaction between the CO_2_ molecule (O) and the *µ*_2_‐OH group (H) of the framework correspondingly. E) GCMC‐simulated 15CO_2_:85N_2_ binary mixture adsorption isotherm of MIL‐120(Al)‐AP‐Str1 at 298 K calculated by GCMC simulations, F) Corresponding snapshot (zoom in) showing the location of CO_2_/N_2_ in MIL‐120(Al)‐AP‐Str1 at 1 bar and 298 K from GCMC simulation (*cf*. full sharpshot in Figure S16, Supporting Information). Color code in A, D, and F: Al(OH)_4_O_2_, gray polyhedra; C, gray; O, light red; H, white (C‐H) and light yellow (O‐H); N, blue. Interactions between CO_2_ molecules and *µ*_2_‐OH groups are represented as green dashed lines. All views are shown along [0 1 1].

The crystal structure of MIL‐120(Al)‐AP was first fully geometry optimized (both atomic positions and cell parameters allowed to relax) in its empty form at the Density Functional Theory (DFT) level. Since it is not possible to experimentally determine the positions of the H atoms of the framework with X‐ray diffraction data, we, therefore, constructed a set of configurations corresponding to different *µ*_2_‐OH orientations that were subsequently geometry‐optimized. Two stable structures were identified, labeled as MIL‐120(Al)‐AP‐Str1 and MIL‐120(Al)‐AP‐Str2, and illustrated in **Figure** **5**A. The structural and textural features of these two DFT‐optimized structures are in line with the experimental data collected on the MIL‐120(Al)‐AP sample (see Table S4, Supporting Information). The X‐ray diffraction patterns calculated for these two structures showing very similar unit cell parameters/cell volume (only 2.4% cell volume difference) (see Table S4, Supporting Information) are in good agreement with that collected experimentally (Figure 5B). The total energies of the two structures differ by 11.29 meV atom^−1^, with MIL‐120(Al)‐AP‐Str1 being the most stable one. Their CO_2_ adsorption isotherms were further calculated at 298 K using Grand Canonical Monte Carlo (GCMC) simulations. Figure 5C shows that the simulated adsorption isotherm for MIL‐120(Al)‐AP‐Str1 is in excellent agreement with the experimental data while a steeper increase of CO_2_ uptake at low pressure is simulated for MIL‐120(Al)‐AP‐Str2. This observation strongly supports that the *µ*_2_‐OH orientation plays a critical role in the CO_2_ affinity of the MOF framework and indeed the most energetically stable MIL‐120(Al)‐AP‐Str1 structure enables to capture of the experimental scenario. Typically, the highly symmetric *µ*_2_‐OH orientations toward the pore channel in MIL‐120(Al)‐AP‐Str2 induces strong direct interactions with CO_2_ (see Figure S15, Supporting Information) leading to a simulated adsorption enthalpy at low coverage higher compared to that calculated for MIL‐120(Al)‐AP‐Str1 (−43.8 vs −39.4 kJ mol^−1^) in line with its steeper adsorption isotherm profile observed at low loading.

This distinct CO_2_ adsorption behavior in MIL‐120(Al)‐AP‐Str1 and MIL‐120(Al)‐AP‐Str2 is illustrated in Figure 5D which reports the DFT‐optimized structures for two different CO_2_ loadings. We observed that at the loading of one CO_2_ per unit‐cell (corresponds to 2.02 mol g^−1^ CO_2_ loading), CO_2_ orientated parallel to the organic linker adopts a highly ordered geometry interacting simultaneously with the four *µ*_2_‐OH groups of MIL‐120(Al)‐AP‐Str2 while for MIL‐120(Al)‐AP‐Str1, CO_2_ preferentially interacts with only two *µ*_2_‐OH groups ($d_{O_{CO2}-O_{\mu 2-\mathrm{OH}}}$ = 3.28 Å). This distinct adsorption behavior is in line with a higher CO_2_ affinity for MIL‐120(Al)‐AP‐Str2 and hence its resulting steeper adsorption isotherm at the initial stage of adsorption. When CO_2_ concentration increases up to two molecules per unit‐cell (corresponds to 4.05 mol g^−1^ CO_2_ loading), Figure 5D also reveals a similar simulated guest distribution in both MIL‐120(Al)‐AP structure models with associated $d_{O_{CO2}-O_{\mu 2-\mathrm{OH}}}$ distances of 3.30 Å and from 3.08 to 3.17 Å for MIL‐120(Al)‐AP‐Str1 and MIL‐120(Al)‐AP‐Str2, which match well with the experimental reported data shown in Figure 4A,B.

Next, the 15CO_2_:85N_2_ binary mixture adsorption isotherm was simulated for MIL‐120(Al)‐AP‐Str1 (Figure 5E). This structure model is predicted to predominantly adsorb CO_2_, while N_2_ uptake is almost negligible over the whole pressure range up to 1 bar. Figure 5F evidences that CO_2_ molecules are mostly located at the same positions as in single components while only a very few N_2_ molecules are adsorbed in the MOF pores. The resulting CO_2_/N_2_ selectivity is comprised between 110–170 (see Figure S17, Supporting Information), which agrees with the IAST estimations obtained from the experimental single component adsorption isotherms, further confirming the great potential of MIL‐120(Al)‐AP for CO_2_ capture from flue gases.

### 2.4 Green Scalable Synthesis

**Figure 6:**
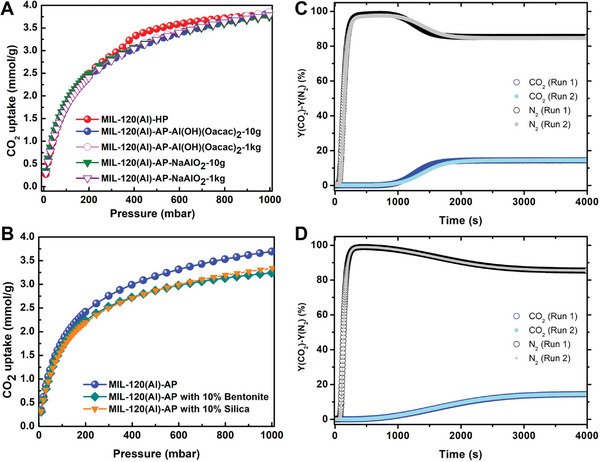
CO_2_ adsorption uptakes of MIL‐120(Al)‐AP. A) CO_2_ adsorption isotherms at 298 K on different scale batch preparations. B) CO_2_ adsorption isotherms comparison between pure and structured samples with 10% Bentonite or Silica. C) Breakthrough curves for MIL‐120(Al)‐AP beads with 10% Si. D) Breakthrough results of MIL‐120(Al)‐AP beads with 10% bentonite. The activation condition for both samples was heating at 50 °C for 12 h under vacuum, run 1 refers to the measurement in dry conditions, run 2 refers to the measurement in dry conditions after exposure to humid conditions, while in between the sample was reactivated at 50 °C under vacuum.

As mentioned earlier, MIL‐120(Al)‐AP possesses a high ratio of metal to ligand (4:1), which is (by far) higher than for the other MOFs. Since the ligand is the main limiting component in MOFs’ production cost, particularly when abundant metal cations are in play,^[^ ^20^ ^]^ this shall be highly advantageous for large‐scale industrial synthesis. To meet with potential use for industrial separation processes where the sorbents need to be produced at a very large scale, first attempts of kilogram‐scale synthesis of the MIL‐120(Al)‐AP solids were carried out (synthesis methods given in Supporting Information) based on the optimized AP synthesis method developed in this work. Considering the cost of aluminum precursors (Table S8, Supporting Information) (Al_2_(SO_4_)_3_ 18H_2_O, Al(NO_3_)_3_ 9H_2_O, AlCl_3_ 6H_2_O) and/or their low solubility, safety or corrosive issues, in addition, their less acidic chemical nature (as discussed in the previous sections), two other precursors were also considered for the synthesis at larger scale (> 100 g). First, the optimized procedure using Al(OH)(CH_3_COO)_2_ was deployed, relying on a 30 L glass line reactor equipped with a pressurized filtration system (Figure S19, Supporting Information), leading to a batch of MIL‐120(Al)‐AP up to 1–2 kg (per single synthesis). Later, a more cost‐effective Al‐precursor, namely NaAlO_2_, among the less expensive Al sources, was explored and served to demonstrate the feasibility of a multi‐kilogram‐scale cheaper synthesis with a higher Space‐Time Yield (STY).^[^ ^39^ ^]^ In both cases, high‐quality MIL‐120(Al)‐AP materials were easily synthesized with a high yield (> 70%) at > 3 kg scales via green methods, as confirmed by SRPD patterns, FT‐IR spectroscopy, TGA, N_2_ adsorption (Figure S20, Supporting Information), CO_2_ adsorption (**Figure** **6**A; Figure S27A, Supporting Information), and SEM/EDX results (Figures S21 and S22, Supporting Information). Noteworthy, the kilogram‐scale and the small‐scale samples exhibited almost the same CO_2_ uptake under the same conditions. When using Al(OH)(CH_3_COO)_2_ as an aluminum precursor, water was the only solvent in the synthesis procedure, and after washing with warm water, the STY reached a moderate value close to 60 kg m^−3^ day^−1^. When using NaAlO_2_ as an aluminum precursor, it was necessary to add acetic acid as a pH modulator to obtain MIL‐120(Al)‐AP because of the too‐high alkaline character of the starting NaAlO_2_/H_2_O solution. The STY value was higher, ≈ 100 kg m^−3^ day^−1^, due to a too higher concentration used for the reaction, which is comparable to the values obtained for zeolites (50 to 150 kg m^−3^ day^−1^) or benchmark Al‐MOFs such as MIL‐160^[^ ^40^ ^]^ or MOF‐303.^[^ ^14^ ^]^ Meanwhile, it is interesting to note the smaller particle sizes of MIL‐120(Al)‐AP obtained when NaAlO_2_ is used instead of Al(OH)(CH_3_COO)_2_ as observed from SEM images (≈ 50 nm vs ≈ 300 nm in large‐scale syntheses; Figure S21, Supporting Information). This could be ascribed to a faster reaction/nucleation between Al(III) and BTeC in the case of the former due to the more basic conditions favoring Al hydroxide condensation. Moreover, to assess the atomic‐scale quality of the scaled‐up samples, solid‐state multinuclear (^1^H, ^13^C, and ^27^Al) NMR (ssNMR) Magic Angle Spinning (MAS) studies were carried out. The ^1^H spectra and their decomposition in three components (Figure S23 and Table S9, Supporting Information) show that neither the synthetic route nor the scale affects significantly the proton structural environments. The ^13^C experiments confirm this analysis: the four spectra are close‐to‐identical and match the literature data. It therefore confirms that the linker local structure and arrangement is the same for all synthesis conditions and that no significant amount of organic impurities can be detected. There is only a small, but significant, increase in linewidth for the aromatic protons and the *µ*_2_‐OH for the large‐scale synthesis done with Al(OH)(CH_3_COO)_2_, showing a compound slightly more disordered than the other three. The ^27^Al ssNMR MAS spectra (Figure S24, Supporting Information) of the four compounds confirm this analysis: the two crystallographic sites are well identified, with all NMR parameters identical within uncertainty (Table S10, Supporting Information). The exception is found again for the Al(OH)(CH_3_COO)_2_ large‐scale synthesis which displays a smoothening of the line shapes, a clear indication of the presence of structural disorder, likely to be due to the higher proportion of defects generated by the residual acetates still embedded within the framework, eventually replacing some BTeC moieties. For the latter, a slightly modified chain arrangement is to be expected based on the reduction in nuclear quadrupole coupling constant *C*_Q_ (8.6 to 7.7 MHz) and quadrupolar asymmetry parameter (0.5 to 0.6) of one of the aluminum sites. Finally, a shoulder at the left side of the peaks observed for the NaAlO_2_ large‐scale synthesis is more intense than the “*n* = 0” spinning sideband of the ←3/2,3/2> transition, and points to the possible presence of an oxide impurity which accounts for ≈6%_at_ of the total aluminum content (although not affecting the adsorption performances).

Considering the requirements of the real application, MOF powders need to be shaped not only to i) avoid the tedious (and possibly risky) manipulation of powder, but also ii) to minimize the pressure drop and thermal gradient across the adsorption column and ensure an optimal fluid and heat diffusion. Here, we have successfully shaped MIL‐120(Al)‐AP using inorganic binders, namely bentonite, and silica, through an extrusion/spheronization method. The CO_2_ uptakes of the MIL‐120(Al)‐AP beads obtained with 10% of silica and 10% of bentonite were in good agreement with the uptake of the MOF in the powder form (Figure 6B; Figure S27B, Supporting Information). Additionally, the MIL‐120(Al)‐AP beads exhibited a high and useful crushing strength particularly when using bentonite (≈38 N for MIL‐120(Al)‐AP with 10% bentonite; ≈ 9 N for MIL‐120(Al)‐AP with 10% silica) (Figures S25 and S26, Supporting Information) which is important for column‐filling robustness during CO_2_ capture applications. The higher mechanical strength of beads obtained with the aluminum silicate bentonite might be due to the strong affinity between Al‐OH groups that are likely to decorate the external surface of bentonite and MIL‐120(Al)‐AP.

### 2.5 Cyclability, Breakthrough Curve Tests

The reusability of MIL‐120(Al)‐AP solids was first investigated upon six consecutive CO_2_ adsorption/desorption cycles (See Table S7, Supporting Information), using different temperatures for the activation. The CO_2_ uptake of MIL‐120(Al)‐AP solids after activating the sample at 298 K under a secondary vacuum during 6 h in the first two measurements exhibited slightly higher values compared with the same sample after activation at 323 K, as depicted in Figure S18 (Supporting Information). This could be due to a slight reorganization of the *µ_2_*‐OH groups, resulting in slightly different CO_2_ uptakes. After a first activation at 323 K, the CO_2_ uptake did however not change much depending on the activation condition, strongly indicating that MIL‐120(Al)‐AP keeps a reproducible CO_2_ uptake due to the dense *µ*_2_‐OH groups in the channels.

Finally, to study the separation performance for 15CO_2_:85N_2_, first, dynamic column breakthrough experiments were performed using a few grams scaled‐up shaped sample, in a packed column filled with activated MIL‐120(Al)‐AP with 10% silica and MIL‐120(Al)‐AP with 10% bentonite with a total flow of 1 NL min^−1^ (experimental set‐up is detailed in Figure S28, Supporting Information). As depicted in Figure 6C,D (run 1), in both cases, highly efficient separation of CO_2_ from the CO_2_/N_2_ mixture could be achieved: N_2_ gas first eluted through the adsorption bed at the beginning of the adsorption process while CO_2_ appears at the outlet of the column from breakthrough time leading to a CO_2_ pure productivity (0.67 mmol cm^−3^ for MIL‐120(Al)‐AP with 10% silica; 0.79 mmol cm^−3^ for MIL‐120(Al)‐AP with 10% bentonite). The breakthrough curves were used to experimentally determine the CO_2_/N_2_ selectivities which are as high as 93 for MIL‐120(Al)‐AP with 10% silica and 98–108 for MIL‐120(Al)‐AP with 10% bentonite, respectively, in excellent agreement with IAST calculated values on the single component adsorption isotherms collected on pure powder form. Additionally, CO_2_/N_2_ adsorption/desorption cycles have been carried out to evaluate the regeneration of both shaped MIL‐120(Al)‐AP under dry and humid conditions (experimental procedure detailed in Supporting Information). In dry conditions, a complete regeneration was observed (Figure S29A,B, Supporting Information) after four cycles without heating or vacuum conditions. However, the same measurements in humid conditions (Figure S29C,D, Supporting Information) highlighted the progressive accumulation of water between each cycle leading to a CO_2_ capacity decrease in the operating conditions (*i*.*e*., without heat or vacuum). After this exposure to moisture, both samples were however fully reactivated at 50 °C under vacuum. The same breakthrough results with a similar CO_2_ adsorbent amount (Run 2 in Figure 6C,D; Table S11, Supporting Information) were observed, strongly indicating that this MOF is very stable and can provide a good repeatability of CO_2_ adsorption with full regeneration after exposure to humid atmosphere, although a slight modification of the breakthrough curves could be observed in the case of beads shaped with silica. It is to be noted that the mass transfer seems slower in the case of the samples shaped with bentonite (Figure 6C,D). This is very likely due to different pore blocking caused by the binder, compared to silica‐shaped beads. Indeed, the dense inorganic Al_4_(OH)_8_ oxo‐chains in MIL‐120(Al) framework could favor a higher interaction with bentonite than silica, leading to slight pore‐blocking behavior which could be inferred from the N_2_ isotherms at 77 K (Figures S25D and S26B, Supporting Information). Since the full regeneration could be achieved by heating (≈ 50 °C) and vacuum, MIL‐120(Al)‐AP appears as a suitable adsorbent well adapted to TSA (thermal swing adsorption) carbon capture under real conditions, but also to V/PSA (vacuum swing adsorption or pressure swing adsorption) conditions when specific considerations are taken into account (i.e., either pre‐drying or very fast cycling process).

### 2.6 Operando Spectroscopic Studies

**Figure 7:**
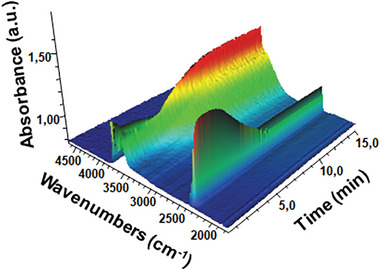
Operando IR spectra. IR spectra in the ν(OH) and ν(C═O) region for MIL‐120(Al)‐AP with time on stream during the first minutes of CO_2_ adsorption in the presence of water (20% CO_2_ and 1% H_2_O in Ar flow at RT).

In order to further evaluate the potential of MIL‐120(Al) for CO_2_ capture at industrial scale, including in the presence of water, and for the regeneration of the MOF, operando infrared (IR) investigations were carried out on the MOF powder in relevant conditions, both as a self‐sustained wafer and deposited on a Si plate (to monitor structural bands), in order to simulate the material behavior at duty. Therefore, the sample was first subjected to an activation step by flowing pure Ar at RT followed by a heating ramp (in Ar) after a steady state was achieved at RT. The desorption of any species, paying particular attention to water molecules, was monitored by Mass Spectrometry (MS) and IR. A first rapid water release was observed, finishing completely after 8 h at RT in Ar flow, as shown in Figure S30 (Supporting Information). After the steady state was achieved at *RT*, the sample was monitored at increasing temperatures (100–300 °C) under the Ar flow. Further water was released in the range of 100–300 °C (Figure S31, Supporting Information). However, residual free acid, if any, could be removed at 200 °C and structural changes are detected from 300 °C. In addition to activation at *RT*, faster removal of water (in <1 h) was evidenced when the activation was carried out in Ar flow at 100 °C. This suggests that MIL‐120(Al) can be activated under mild conditions. Nevertheless, the high density of *µ*_2_‐OH groups in MIL‐120(Al) leads to a highly hydrophilic character (consistent with the experimental water adsorption isotherms (Figure S1E, Supporting Information)). Thus, we aimed to deeply investigate the effect of water during CO_2_ adsorption after activation. Operando IR spectra were collected at room temperature during the adsorption of CO_2_ in MIL‐120(Al)‐AP in the presence of 1% H_2_O (a maximum concentration that could be envisaged in a CO_2_ capture process after a chiller) and compared to the same conditions but in dry CO_2_/Ar flow. **Figure** **7** shows the operando IR spectra recorded versus time during wet CO_2_ adsorption. At the beginning of the adsorption process (during ≈ 2 min), a very fast CO_2_ adsorption is observed, as witnessed by the characteristic band at 2338 cm^−1^; meanwhile no H_2_O adsorption occurs (the IR band ≈ 3500 cm^−1^ associated with stretching vibrations of OH‐bonds remains constant and the characteristic combination band associated to H_2_O at 5170 cm^−1^ is still absent). Then, after ≈ 7 min, the bands of CO_2_ progressively decrease concomitantly to the slow increase of H‐bonds absorbance, revealing that initially CO_2_ successfully competes with water on the adsorption sites. However, after 10 min, a drastic drop in the band of CO_2_ and a stable band intensity for the H_2_O groups are observed, indicating that the adsorbed CO_2_ molecules have been partially replaced by H_2_O molecules. This phenomenon is also observed in the MS signal, confirming that MIL‐120(Al) exhibits much faster adsorption kinetics for CO_2_ than for water molecules, before reaching an equilibrium favorable to water. To further clarify the rapid CO_2_ adsorption in the presence of water, CO_2_ adsorption was analyzed in dry or wet Ar flow at different CO_2_ concentrations. As represented in Figure S32 (Supporting Information), the presence of H_2_O does not decrease the CO_2_ uptake compared with the dry conditions (considering the maximal adsorption before any CO_2_ removal by H_2_O in long‐time term). On the contrary, small amounts of water in the flow seem to even promote the CO_2_ uptake. Furthermore, the uptake of H_2_O decreases slowly with increasing CO_2_ concentration in the Ar flow, demonstrating that CO_2_ can suppress water adsorption to a certain extent favored by kinetics. Figure S33 (Supporting Information) (MS) and Figures S34 and S35 (Supporting Information) (IR) report the quantified amount of CO_2_ and H_2_O adsorbed in the material. It is important to note, first, that in situ IR spectra of the activated sample (Figure S35, Supporting Information) showed four distinct and sharp ν(O‐H) stretching bands revealing four OH groups with relatively different environments in the structure, which is the first time observed in a MOF, to the best of our knowledge. These OH groups appear insensitive to activation under a vacuum up to 150 °C. Furthermore, we observe that the amount of CO_2_ adsorbed in the MIL‐120(Al)‐AP framework is proportional to CO_2_ partial pressure in the flow. For instance, the intensity of CO_2_ under 30% CO_2_ concentration in the gas flow exhibits the highest absorbance in IR spectra. Combining these results with those from the MS signal, we can confirm that the presence of H_2_O does not decrease the CO_2_ uptake at the beginning of the adsorption process. Additionally, the spectra of the hydroxyls, with time on stream in a flow containing CO_2_, show that these groups are only slightly perturbed by the introduction of small aliquots of CO_2_ and water in the cell and confirms the model represented in Figure 5, showing experimentally the interaction of the CO_2_ molecules with four OH groups, inducing a progressive decrease in the pristine peaks (activated sample) and the formation of H‐bonding broad bands proportionally to the CO_2_ partial pressure (Figure S35, Supporting Information). After 1 h, however, the CO_2_ uptake significantly decreases, showing that H_2_O saturates the material during time on stream, significantly removing most of the adsorbed CO_2_. Additionally, the effect of the NOx and SOx was evaluated under the complete flow. While SOx adsorption is not observed, the uptake of NO decreases on increasing CO_2_% fed to the flow and reaches only 4.07 – 4.08 micromol/gMOF for 20% CO_2_ and 1% H2O in the feed (Figure S36, Supporting Information). Interestingly, the adsorbed NO is completely desorbed under inert gas at RT while no sign of MOF degradation was observed after exposure to NOx and SOx (Figure S37, Supporting Information).

Therefore, these operando IR studies confirm the potential of MIL‐120(Al) for the CO_2_/N_2_ kinetic separation even in the presence of water (to some extent) or other contaminants, despite the hydrophilic behavior revealed by the water isotherms and the preliminary results showed by dynamic breakthrough measurements operated at flowrate setpoint of 1 NL h^−1^. This would call, however, for a specific process design taking into account the difference in adsorption kinetics between CO_2_ and H_2_O as well as a regeneration of the sorbent, particularly water, as it is typically done in V/PSA processes. In this regard, suitable dynamic breakthrough measurements with careful tuning of the stream flow rate and dimension of the column should be carried out for optimal process design.

### 2.7 Techno‐Economic Analysis for MIL‐120(Al)‐AP Production

Based on the synthesis at a pilot lab scale (15 L scale), we propose herein an industrial‐scale production process. A batch process design was considered based on process synthesis heuristics, considering a reactor STY of 100 kg m^−3^ day^−1^. The flowsheet with the main block diagrams of the process is shown in Figure S38 (Supporting Information). The process rationale followed the same methodology that was previously described by some of us where the raw materials are fed to a batch‐stirred reactor, the resulting solid is retrieved by filtration, followed by washing, drying, and storage.^[^ ^20^ ^]^ All the equipment was designed for a yearly production of 1 kton (the rationale behind this value was presented in the Introduction), which would represent ≈ only 5% of the total amount of adsorbent required to capture CO_2_ on a yearly basis for the world cement industry's plants. From the equipment size, an investment in base equipment of 2.4 M$ (2022 prices) was estimated. Using common chemical engineering process cost factor estimation methods, we calculated a total investment of 13.9 M$ (2022 prices), as detailed in Tables S12 and S13 (Supporting Information). This investment already includes the capital costs for a 60% investment loan and a project time span of 12 years (2 years for construction and 10 years of operation for amortization; more details in Supporting Information).

The production cost of MIL‐120(Al) was estimated based on the manufacturing costs (direct, indirect, and fixed), and general expenses, as explained in detail in Table S14 (Supporting Information). The final calculated production cost is 12.94 $ kg^−1^ (2022 prices), which is significantly lower than previous estimates, based on raw materials costs obtained from laboratory chemical reagents catalogs (2916.5 $ kg^−1^).^[^ ^25^ ^]^ Compared with previous estimates for MIL‐160(Al) made by some of us in 2019 (29.5 $ kg^−1^ for 1 kton per year),^[^ ^20^ ^]^ the MIL‐120(Al) production cost scaled to 2019 prices is significantly lower (9.86 $ kg^−1^). This is due to two cumulative factors related to the ligand that are favorable for MIL‐120(Al): the lower ligand‐to‐aluminum mass ratio and the lower price of the ligand. The BTeC ligand in MIL‐120(Al) is (at least) ten times less expensive than the 2,5‐furandicarboxylic acid ligand in MIL‐160(Al). Some of us have also recently estimated a production cost for MIL‐100(Fe) of 30 $ kg^−1^ for a 1 kton per year production,^[^ ^41^ ^]^ which is also higher than that for MIL‐120(Al).

**Figure 8:**
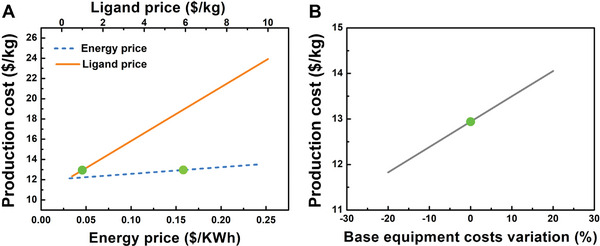
Sensitivity analysis of MIL‐120(Al)‐AP production costs. A) Effect of the ligand and energy costs; B) effect of the base equipment costs. The green dots over the lines represent the base economic scenario (2022 prices).

To better understand how the estimated production cost could vary, we performed a sensitivity analysis of our economic model. From the structure of the direct production costs (Table S14, Supporting Information), one can observe that the main contribution arises from the raw materials (≈ 45%). From these, the ligand price is the most significant and prone to variations (sodium aluminate and acetic acid are common raw materials used in the industry). Thus, we selected the ligand price as one of the variables for the sensitivity analysis. Since the price of electricity has increased significantly over the last three years,^[^ ^42^ ^]^ we have selected this factor as another variable for the sensitivity analysis. We can observe that the ligand price has a stronger influence on the production cost, with a variation from 12.33 to 23.92 $ kg^−1^ when the price of the ligand varies between 0.5 and 10 $ kg^−1^ (**Figure** **8**A). The energy cost has a lower impact on the production cost, with a variation of 12.13 to 13.52 $ kg^−1^, considering a span of energy prices from 0.032 to 0.242 $ kW^−1^ h^−1^. Since investment estimates based on historical equipment prices are always approximations, and the chemical engineering equipment cost has been increasing significantly in recent years,^[^ ^43^ ^]^ we have also looked at the influence of the base equipment cost on the production cost (Figure 8B). A variation of ± 1.11 $ kg^−1^ is observed when the base equipment cost varies by ± 20%. Thus, the production cost of MIL‐120(Al) seems to be more influenced by the ligand price but, nevertheless, it remains significantly lower than other previous estimations for this and other MOFs. For larger production scales, this cost is expected to be even lower due to the principle of the economy of scale. Moreover, with a prospect of some further improvements in the synthesis procedure, we could, for instance, by shortening the synthesis duration to 13 h, significantly increase the STY = 190 kg m^−3^ day^−1^) without hampering the product quality and CO_2_ performance (Figure S39, Supporting Information). Therefore, this will bring the estimation cost to even more attractive values and lead to higher production per year.

## 3 Conclusion

In this work, a highly robust and cost‐effective aluminum‐based microporous Al tetracarboxylate MOF, MIL‐120(Al), was thoroughly studied in view of post‐combustion carbon capture applications. An environmentally friendly and economically viable ambient pressure synthesis route was developed leading to pure MIL‐120(Al)‐AP samples, as an alternative to the previously high‐temperature hydrothermal route. A combination of experimental and computational adsorption results first confirmed the high and selective CO_2_ uptake of this MOF at low pressure, due to an adequate confined space combining the high density of OH groups on the edge sharing Al chains of octahedra and packed phenyl groups aligned along the inner walls of the channels. While the exceptional thermal and hydrolytic stability of this MOF was demonstrated, a moderate CO_2_ enthalpy of adsorption was deduced of ≈ ‐40 kJ mol^−1^, comparable to benchmark MOF physisorbents, which is an asset for its potential use for CO_2_ capture applications. Besides, a kilogram‐scale synthesis protocol for MIL‐120(Al)‐AP was obtained with a high STY, using inexpensive commercially available precursors via a green and cheap ambient pressure route. MIL‐120(Al)‐AP was also shaped with bentonite and silica which provided good mechanic strength while keeping the sorption performances. Then, the efficient CO_2_ adsorption performance of this MOF was further confirmed thanks to dynamic column breakthrough experiments, while fundamental operando IR studies suggested, to some extent, the potential to operate in humid conditions through a kinetic separation process. Since MIL‐120(Al)‐AP is made from cost‐effective raw materials through a sustainable water‐based and scalable synthesis method, a techno‐economic evaluation of the total industrial production cost indicated a ≈ 13 $ kg^−1^ at the kton‐scale, among the lowest ever reported for MOFs. This, in addition to its adsorption characteristics, easy synthesis, low cost, and robustness, suggests that MIL‐120(Al)‐AP lies as one of the scarce MOFs that meet the necessary criteria for its practical use in real‐life post‐combustion carbon capture processes. In particular, this material could be interesting for TSA processes where regeneration could be completed in the presence of humidity at 50 °C, a low temperature that is relatively easy to obtain from waste heat from industrial processes such as cement or steel production.

## Conflict of Interest

The authors declare that they have no conflict of interest.

## Author Contributions

The manuscript was written through the contributions of all authors. B.C. performed the synthesis and the basic characterization. D.F. and G.M. contributed to the theoretical studies. I.D. and C.M. performed the in situ SRPD measurements and I.D. performed the crystal structure data analysis. S.N. contributed to the gas and vapor sorption isotherms measurements and the analysis of associated data. R.P. and D.C. contributed toward the scale‐up and shaping studies with the help and guidance of F.N., I.C., and P.F. performed solid state multinuclear (^1^H, ^13^C, and ^27^Al) NMR (ssNMR) Magic Angle Spinning (MAS) studies. N.G.M., A.V., and M.D. performed, analyzed, and discussed the IR experiments. N.H. and G.D.W. performed adsorption isotherm and the dynamic breakthrough curve measurements, calculated IAST selectivity and isosteric enthalpy of adsorption, and contributed toward identifying the potential in CO_2_ capture processes. M.B., A.A.M., and M.P. investigated the techno‐economic analysis. G.M. and C.S. supervised the work and contributed toward the overall concept.

## Supporting information

Supporting Information

## Data Availability

The data that support the findings of this study are openly available in ChemRxiv at https://doi.org/10.26434/chemrxiv‐2023‐ck7ft, reference number 2023.
