# Supplementary material for: A Scalable Robust Microporous Al‐MOF for Post‐Combustion Carbon Capture
Source: Adv Sci (Weinh). 2024 Mar 25;11(21):2401070. doi: 10.1002/advs.202401070 (PMC11151012; doi:10.1002/advs.202401070)
Supplement: Supplementary file 1 — Supporting Information [file ADVS-11-2401070-s001.pdf]

ADVANCED SCIENCE
Open Access

Supporting Information

for *Adv. Sci.*, DOI 10.1002/advs.202401070

A Scalable Robust Microporous Al-MOF for Post-Combustion Carbon Capture

*Bingbing Chen, Dong Fan, Rosana V. Pinto, Iurii Dovgaliuk, Shyamapada Nandi, Debanjan Chakraborty, Nuria García-Moncada, Alexandre Vimont, Charles J. McMonagle, Marta Bordonhos, Abeer Al Mohtar, Ieuan Cornu, Pierre Florian, Nicolas Heymans, Marco Daturi, Guy De Weireld, Moisés Pinto, Farid Nouar, Guillaume Maurin*, Georges Mouchaham* and Christian Serre**

# -Supporting Information-

## A scalable robust microporous Al-MOF for post-combustion carbon capture

*Bingbing Chen,[1] Dong Fan,[2] Rosana V. Pinto,[1,8] Iurii Dovgaliuk,[1] Shyamapada Nandi,[1] Debanjan Chakraborty,[1] Nuria García-Moncada,[3] Alexandre Vimont,[3] Charles J. McMonagle,[4] Marta Bordonhos,[5,6] Abeer Al Mohtar,[5] Ieuan Cornu,[7] Pierre Florian,[7] Nicolas Heymans,[8] Marco Daturi,[3] Guy De Weireld,[8] Moisés Pinto,[5] Farid Nouar,[1] Guillaume Maurin,[2]* Georges Mouchaham[1]* and Christian Serre[1]*.*

[1] Institut des Matériaux Poreux de Paris, Ecole Normale Supérieure, ESPCI Paris, CNRS, PSL University, 75005 Paris, France.

[2] ICGM, Univ. Montpellier, CNRS, ENSCM, 34293 Montpellier, France.

[3] Normandie Université, ENSICAEN, UNICAEN, CNRS, Laboratoire Catalyse et Spectrochimie, 14000 Caen, France.

[4] Swiss–Norwegian Beamlines, European Synchrotron Radiation Facility, 71 Avenue des Martyrs, 38000 Grenoble, France.

[5] CERENA, Departamento de Engenharia Química, Instituto Superior Técnico, Universidade de Lisboa, 1049-001 Lisboa, Portugal.

[6] CICECO – Aveiro Institute of Materials, Department of Chemistry, University of Aveiro, Campus Universitário de Santiago, 3810-193 Aveiro, Portugal.

[7] Centre National de la Recherche Scientifique (CNRS), UPR3079 CEMHTI, Université d'Orléans, 1D Av. Recherche Scientifique, CEDEX 2, 45071 Orléans, France.

[8] Service de Thermodynamique et de Physique Mathématique, Faculté Polytechnique, Université de Mons, 7000 Mons, Belgium.

**Emails:** guillaume.maurin1@umontpellier.fr; georges.mouchaham@ens.psl.eu; christian.serre@ens.psl.eu

## Contents


**Chapter 1 Experimental procedures ........ 3**

1.1 Chemicals ........ 3
1.2 Instruments ........ 3
1.2.1 Powder X-ray Diffraction (PXRD) ........ 3
1.2.2 Gas adsorption experiments ........ 3
1.2.3 Thermogravimetric analyses (TGA) ........ 4
1.2.4 Infrared investigations ........ 4
1.2.5 Variable temperature powder diffraction (VT-PXRD) ........ 5
1.2.6 Scanning transmission microscopy (SEM) and energy dispersive X-ray spectroscopy ........ 5

**Chapter 2 Synthesis procedures ........ 6**

2.1 Traditional synthesis of MIL-120(Al)-HP (HP stands for high pressure) ........ 6
2.2 Synthesis optimization of MIL-120(Al)-AP (AP stands for ambient pressure) 6
2.3 Synthesis optimization of MIL-120(Al)-AP in small scale ........ 8

**Chapter 3 Ideal Adsorption Solution Theory (IAST) ........ 11**

**Chapter 4 Stability of MIL-120(Al) ........ 14**

4.1 Thermal stability ........ 14
4.2 Water stability test ........ 14

**Chapter 5 *In situ* temperature- and pressure- variable gas-loading synchrotron radiation powder diffraction (SRPD) ........ 16**

5.1 *In situ* SRPD of MIL-120(Al)-HP ........ 16
5.1.1 *In situ* SRPD of MIL-120(Al)-HP under dynamic vacuum ........ 16
5.1.2 *In situ* SRPD of MIL-120(Al)-HP after $CO_2$-loading ........ 20
5.2 *In situ* SRPD of MIL-120(Al)-AP ........ 22
5.2.1 *In situ* SRPD of MIL-120(Al)-AP under dynamic vacuum ........ 22
5.2.2 *In situ* SRPD of MIL-120(Al)-AP after $CO_2$-loading ........ 25

**Chapter 6 Simulation and modeling ........ 27**

6.1 Computational methods ........ 27

**Chapter 7 Reusability of MIL-120(Al)-AP ........ 31**

**Chapter 8 Synthesis optimization of MIL-120(Al)-AP in large-scale ........ 32**

**Chapter 9 Solid state NMR studies ........ 35**

9.1 Solid state NMR studies ........ 35

**Chapter 10 Shaping of MIL-120(Al)-AP ........ 38**

10.1 Shaping MIL-120(Al)-AP with 10% bentonite ........ 39
10.2 Shaping MIL-120(Al)-AP with 10% Silica ........ 40

**Chapter 11 Breakthrough curve measurements ........ 41**

**Chapter 12 *In situ* IR studies ........ 43**

**Chapter 13 Techno-economic analysis of MIL-120(Al)-AP production costs .. 48**

**Chapter 14 References ........ 55**

## Chapter 1 Experimental procedures

### 1.1 Chemicals

All chemicals were purchased from commercial suppliers and used as received without further purification. 1,2,4,5-Benzene-tetracarboxylic acid (Acros Organics/thermo), glacial acetic acid (Fisher Scientific), aluminum acetate (thermo), aluminum nitrate nonahydrate (Acros Organics), and Sodium aluminate (Fisher Scientific).

### 1.2 Instruments

#### 1.2.1 Powder X-ray Diffraction (PXRD)

High-throughput Bruker D8 Advance diffractometer working on transmission mode and equipped with a focusing Göbel mirror producing CuKα radiation (λ = 1.5418 Å) and a LynxEye detector.

#### 1.2.2 Gas adsorption experiments

Nitrogen sorption data at 77 K was collected on a Micromeritics Tristar instrument. The $CO_2$, $N_2$ and $H_2O$ isotherms at 298 K were recorded on a Micromeritics Triflex instrument. In all the cases, the measurements were recorded using ultra-high purity gases (≥ 4.8 grade). Prior to the nitrogen sorption data at 77 K measurement, the sample was degassed at 150 °C for six hours in order to remove all free water molecules from the pores. Prior to the $CO_2$, $N_2$ and $H_2O$ isotherms at 298 K measurement, the sample was degassed at 50 °C for six hours. The degassing was done in one step using a Micromeritics SmartVacPrep degas unit: evacuation at 150 or 50 °C on the degas port ($p$ =$10^{-6}$ mbar), at which point the outgas rate was ≤ 2 $\mu$bar $min^{-1}$.

Six cycle $CO_2$ adsorption at 298 K measurements were collected on intelligent gravimetric adsorption (IGA) using the degas unit: evacuation at 25 and 50 °C. In these six cycle tests, the first three desorption processes were carried out without air exposure, and the rest were exposed to air.

In order to calculate the isosteric enthalpy of adsorption ($Q_{st}$) and $CO_2/N_2$ selectivity,

pure component ($CO_2$ and $N_2$) adsorption isotherm measurements by gravimetric technique have been performed at 298 K, 308 K and 318 K using an apparatus built in-house around a high-pressure magnetic suspension balance marketed by Rubotherm. In order to use IAST correctly, $CO_2$ adsorption isotherms have been measured up to 1 bar while $N_2$ adsorption isotherms have been measured up to 50 bar. This system allows to separate the microbalance (resolution: 10 $\mu$g) from the adsorption chamber in which stand pressure, temperature and corrosive operating conditions. The magnetic system couples an electromagnet linked to the balance with a permanent magnet linked to the crucible containing the adsorbent. Moreover, it avoids subcritical gas condensation. The system allows measurements in a pressure range of 0-150 bar and in a temperature range of 233.15-403.15 K. The adsorbent sample (around 1 g) is exposed to different gases. The sample mass variation $m_{meas}$ (g) is measured as well as pressure and temperature when the thermodynamic equilibrium is reached (with our criterion, when four of the five last mass measurements (noticed each 5 min) are included in an interval of 50 µg). The buoyancy effect of the gas phase on the adsorbent volume $V_{ads}$ ($m^3$) is then corrected to determine the excess adsorbed mass $m_{exc}$ (g) as:

$$m_{exc} = m_{meas} + \rho_{gas} \cdot V_{ads}$$

The gas phase density, $\rho_{gas}$ ($g.m^{-3}$), is determined using an appropriate equation of state for $CO_2$[1] and $N_2$[2]; the adsorbent volume $V_{ads}$ is evaluated by direct helium buoyancy effect measurement since helium is not adsorbed at high pressure (10 bar up to 100 bar). The pressure is measured with two pressure transmitters (MKS Baratron 631 D up to 1.33 bar and Tecsis-Series P3382 up to 160 bar). More details about the installation and the methodology can be found in literature.[3]

### 1.2.3 Thermogravimetric analyses (TGA)

Mettler Toledo TGA/DSC 2, STAR System apparatus with a heating rate of 5 °C $min^{-1}$ under oxygen flow. Mettler Toledo FiveEasyTM Plus pH/mV bench meter.

### 1.2.4 Infrared investigations

*In situ* IR analyses were performed in a Nicolet iS5 FTIR ThermoFisher spectrometer, using a DTGS detector, accumulating 64 scans at a resolution of 4 $cm^{-1}$. 1.17 mg of sample powder were deposited on a Si plate, in order to distinguish the structural bands. The sample spectra were recorded as is and after an evacuation treatment (~$10^{-6}$ mbar)

at room temperature, 100 °C and 150 °C to remove impurities and residual species. *Operando* experiments were performed in a dedicated system including mass flow controllers for feeding gas into the lines,[4] equipped with a Nicolet iS5 FTIR ThermoFisher spectrometer, a "Sandwich" reactor cell [4 $cm^{-1}$], an IR gas cell analyzer, a quadrupole mass spectrometer (Pfeiffer Omnistar GSD 301). Spectra were recorded in transmission mode via a MCT detector, accumulating 64 scans at a resolution of 4 $cm^{-1}$. The sample was deposited on a Si plate as in the case of *in situ* analysis.

### 1.2.5 Variable temperature powder diffraction (VT-PXRD)

The laboratory variable temperature powder diffraction (VT-PXRD) data were collected with Empyrean (Malvern Panalytical) diffractometer equipped with a HTK-1200N (Anton Paar) high-temperature chamber and a GaliPIX$^{3D}$ 2D Hybrid Pixel detector (Cu Kα radiation). PXRD patterns were collected every 25 °C from room temperature to 350 °C, with two hours scan for each temperature.

### 1.2.6 Scanning transmission microscopy (SEM) and energy dispersive X-ray spectroscopy

FEI Magellan 400 scanning electron microscope, the energy-dispersive X-ray spectroscopy (EDX) analysis allows performing chemical mapping using the "Super-X" systems for EDX analysis that equipped the microscope.

## Chapter 2 Synthesis procedures

### 2.1 Traditional synthesis of MIL-120(Al)-HP (HP stands for high pressure)

Based on previously reported synthesis procedure, we did a slight modification to the hydrothermal reaction of MIL-120(Al).[5] In a 23 mL Teflon reactor, 1,2,4,5-benzene-tetracarboxylic acid (BTeC 1 mmol, 254 mg) was dissolved with DI water (10 mL) and further sodium hydroxide (NaOH 8 mmol, 320 mg) was added and the suspension solution changed into a clear solution. Then, aluminum nitrate ($Al(NO_3)_3 \cdot 9H_2O$ 4 mmol, 1.5 g) was added slowly into the above solution. After stirring for 10 min, the reactor was finally put into a pre-heated oven at 180 °C for 24 hours. When the reactor was cooled down to room temperature, the white product was collected by centrifugation (3 min at 10,000 rpm). The slurry product was washed with water (100 mL) overnight at 45 °C in a round bottomed flask. Finally, the sample was collected after drying in air for two days.

### 2.2 Synthesis optimization of MIL-120(Al)-AP (AP stands for ambient pressure)

#### 10 g MIL-120(Al)-AP using aluminum hydroxyacetate ($Al(OH)(OAc)_2 \cdot 1.38H_2O$)

To a 500 mL round-bottom flask, DI water (300 mL), BTeC (20 mmol, 5.08 g), and $Al(OH)(OAc)_2 \cdot 1.38H_2O$ (80 mmol, 15 g) were added with stirring at room temperature. Then the mixture was heated up to 80 °C, and kept at this temperature for 72 hours. The white crude product was collected by filtration or by centrifugation (3 min at 10,000 rpm). The final product was obtained by washing in DI water (200 mL) at 50 °C overnight and finally the sample was collected and dried in air overnight.

#### 100 g MIL-120(Al)-AP using aluminum hydroxyacetate

To a 5 L reactor, DI water (3 L), BTeC (0.2 mol, 50.8 g) and $Al(OH)(OAc)_2 \cdot 1.38H_2O$ (0.67 mol, 125 g) and were mixed and the reaction was conducted under reflux under stirring (200 rpm) for 24 hours. After that time, the white crude product was collected by filtration and the crude sample was washed in boiling DI water (3 L) for 18 hours two times. The sample was finally obtained after filtration and dried in air.

**1 kg MIL-120(Al)-AP using aluminum hydroxyacetate**

To a 30 L reactor, DI water (16 L) after reaching 80 °C, BTeC (2.13 mol, 542 g) and $Al(OH)(OAc)_2 \cdot 1.38H_2O$ (8.57 mol, 1.39 kg) were added and the suspension was heated up to 100 °C and kept under stirring at 200 rpm for 24 hours. The white crude product was collected by filtration. Around 1.5 kg of as-synthesized sample was obtained, and further washed two times with boiling DI water overnight. The washed sample obtained (STY = 60 kg $m^{-3}$ $day^{-1}$) was dried in air overnight.

**1 g MIL-120(Al)-AP using sodium aluminate**

To a 100-mL round-bottom flask, DI water (15 mL), BTeC (1 mmol, 254 mg), $NaAlO_2$ (4 mmol, 328 mg) and acetic acid (0.48 mL) were mixed and the solution was heated at 140 °C for 24 hours. The white crude product could be collected by filtration or by centrifugation (3 min at 10,000 rpm). The final product was obtained by washing in DI water (200 mL) at 40 °C overnight. Finally, the sample was collected after filtration and dried in air.

**100 g MIL-120(Al)-AP using sodium aluminate**

To a 5 L reactor, DI water (800 mL), BTeC (160 mmol, 40.64 g), sodium aluminate (640 mmol, 52.48 g) and acetic acid (73.6 mL) were added slowly into the above suspension. Then, the reflux reaction was carried out for 24 hours. The white crude product was collected by filtration and further washed in boiling DI water (800 mL) overnight twice. The sample was obtained after filtration and dried in the vacuum oven.

**1 kg MIL-120(Al)-AP using sodium aluminate**

To a 30 L reactor, DI water (15 L), BTeC (3.99 mol, 1.016 kg), sodium aluminate (16.005 mol, 1.31 kg) and acetic acid (1.84 L) were added with stirring at RT. The suspension was heated up to 100 °C and the reflux reaction was carried out for 24 hours. The white crude product was collected by filtration. The crude sample was washed in boiling DI water (15 L) overnight two times. The sample (STY ca. 100 kg $m^{-3}$ $day^{-1}$) was finally obtained after filtration and dried in vacuum oven.

### 2.3 Synthesis optimization of MIL-120(Al)-AP in small scale

**Table S1**. All trials involved in the synthesis optimizations of MIL-120(Al)-AP. M(Al(OH)(OAc)$_2$) = 162 g mol$^{-1}$ (Ac = acetate) without 1.38 crystalized water; M(Al(OH)$_3$) = 78 g mol$^{-1}$; M(NaAlO$_2$) = 82 g mol$^{-1}$; M(BTeC) = 254 g mol$^{-1}$. The number of the crystalized water in Al(OH)(OAc)$_2$ was calculated by TGA in $O_2$. The product from the highlighted synthesis procedure in the list has been used for PXRD, IR, TGA, $N_2$ adsorption at 77 K and $CO_2$ adsorption at 298 K.

| Al source (mmol) | BTeC (mmol) | $H_2O$ (mL) | Acid or base (mmol) | Starting pH | Heating plate temperature (°C) | Reaction time (hour) | Result phase |
|---|---|---|---|---|---|---|---|
| 4 Al(OH)$_3$ | 1.5 | 15 | 16 NaOH | >14 | 140 | 24 | MIL-121 |
| 4 Al(OH)$_3$ | 1.2 | 15 | 12 NaOH | >14 | 140 | 24 | MIL-121 |
| 4 Al(OH)$_3$ | 2 | 15 | - | 2 | 140 | 24 | MIL-121 |
| 4 Al(OH)$_3$ | 1 | 15 | 8 acetic acid | - | 140 | 24 | MIL-121 |
| 4 Al(OH)$_3$ | 1 | 15 | 4 acetic acid | - | 140 | 24 | MIL-121 |
| 8 Al(OH)$_3$ | 2 | 15 | 24 acetic acid | - | 140 | 13 | MIL-121 |
| 8 Al(OH)$_3$ | 2 | 15 | 36 acetic acid | - | 140 | 13 | MIL-121 |
| 8 Al(OH)$_3$ | 2 | 15 | 48 acetic acid | - | 140 | 13 | MIL-121 |
| 16 Al(OH)(OAc)$_2$ | 4 | 30 | - | - | 120 | 24 | MIL-120 |
| 8 Al(OH)(OAc)$_2$ | 2 | 30 | - | - | 140 | 6 | MIL-120 |
| 8 Al(OH)(OAc)$_2$ | 2 | 30 | - | - | 140 | 12 | MIL-120 |
| 8 Al(OH)(OAc)$_2$ | 2 | 30 | - | - | 140 | 24 | MIL-120 |
| 8 Al(OH)(OAc)$_2$ | 2 | 30 | - | - | 140 | 48 | MIL-120 |
| **8 Al(OH)(OAc)$_2$** | **2** | **30** | **-** | **-** | **80** | **72** | **MIL-120** |
| 4 Al(OH)(OAc)$_2$ | 1 | 15 | - | 2-3 | 140 | 72 | MIL-120 |
| 2 Al(OH)(OAc)$_2$ | 1 | 15 | - | - | 140 | 24 | MIL-120 |
| 2 Al(OH)(OAc)$_2$ | 0.5 | 15 | - | - | 140 | 24 | MIL-120 |
| 4 Al(OH)(OAc)$_2$ | 0.5 | 30 | - | - | 140 | 24 | MIL-120 |
| 4 NaAlO$_2$ | 1 | 15 | - | - | 140 | 72 | Amorphous |
| 4 NaAlO$_2$ | 1 | 15 | 8 acetic acid | 3-4 | 140 | 24 | MIL-120 |
| 4 NaAlO$_2$ | 1 | 15 | 4 acetic acid | 3-4 | 140 | 24 | MIL-120 |
| 32 NaAlO$_2$ | 8 | 30 | 32 acetic acid | - | 120 | 24 | MIL-120 |

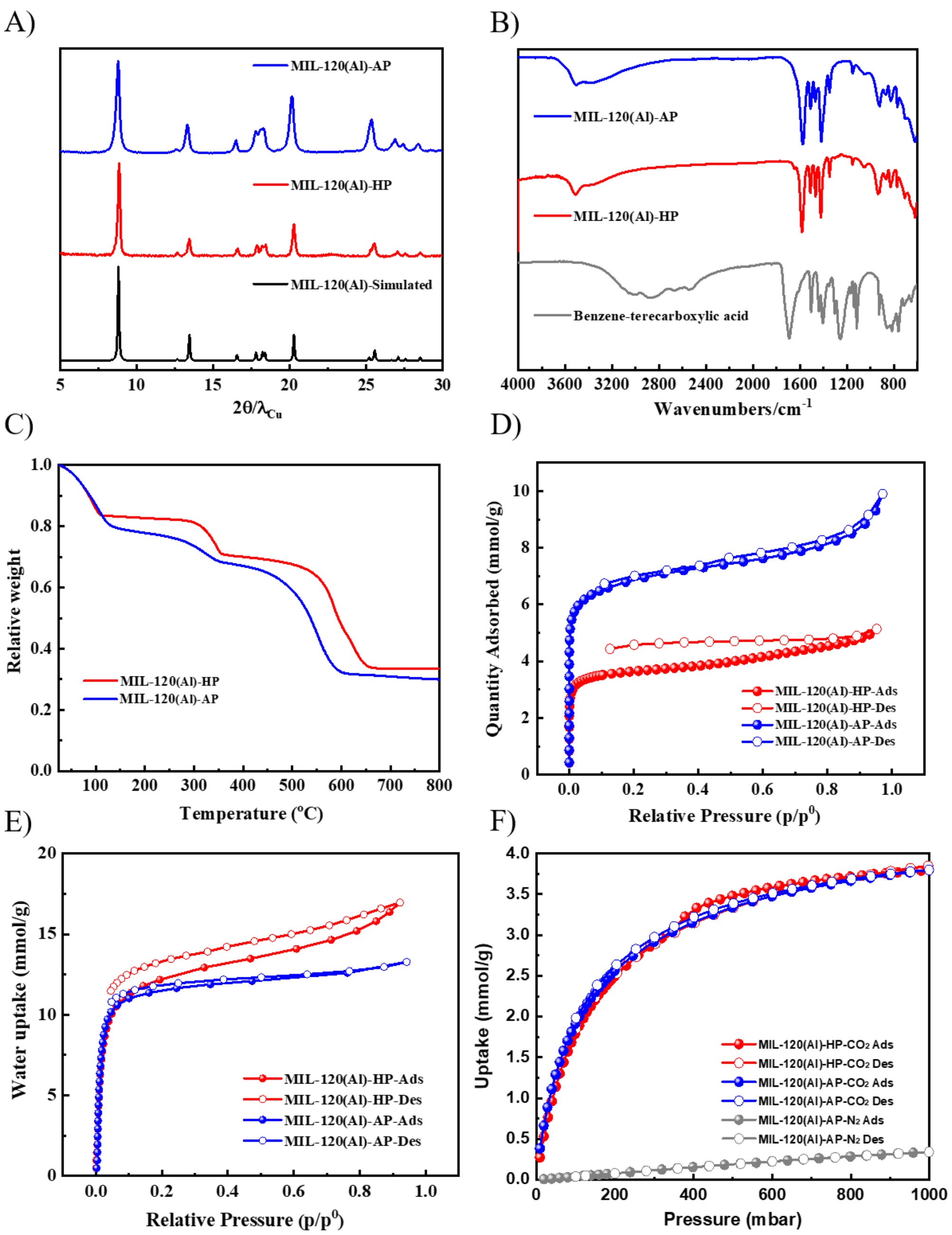


**Figure S1**. Comparison of MIL-120(Al)-HP and MIL-120(Al)-AP: A) PXRD patterns. MIL-120(Al) could be obtained successfully using modified synthesis method. B) FT-IR spectroscopy. Absence of free carboxylic acid groups in MIL-120(Al)-AP from the C=O stretch vibration at 1705 $cm^{-1}$, and large vibration (around 3514 $cm^{-1}$) assigned to hydroxyl group could be observed in both MIL-120(Al)-HP and MIL-120(Al)-AP structures. C) TGA curves obtained under $O_2$ flux (5 °C $min^{-1}$). The MIL-120(Al)-AP sample contains around 3.7% more water than the MIL-120(Al)-HP sample as can be

observed during free water release stage, which was consistent with a 3.8% lower weight of the MIL-120(Al)-AP sample at the end of measurement. D) $N_2$ sorption isotherms at 77 K. $N_2$ uptake of MIL-120(Al)-HP at 77 K was consistent with the data previously reported in 2009.[5] For the MIL-120(Al)-AP sample, the $N_2$ uptake at 77 K is almost two times higher using proper washing procedure. E) Water sorption isotherms at 298 K. F) $CO_2$ and $N_2$ sorption isotherms at 298 K. For all isotherms, adsorption and desorption branches are represented in filled and empty symbols, respectively.

## Chapter 3 Ideal Adsorption Solution Theory (IAST)

This model developed by Myers and Prausnitz[6] allows to predict mixture adsorption equilibrium from only pure compound adsorption isotherms. This requires an expression that represents as accurately as possible the pure compound adsorption isotherms. Herein, the Langmuir equation[7] is used due to the homogeneity of the pore environment:

$$q = q_s \frac{bp}{1 + bp}$$

with $q_s$, the adsorbed amount at saturation, $b$, the adsorption constant. The $CO_2$ adsorption isotherms were carried out until 1 bar while the $N_2$ adsorption isotherms were carried out until 50 bar for a correct use of IAST.[8]

The selectivity is then calculated by:

$$S_{1,2} = \frac{q_1/q_2}{p_1/p_2}$$

With $p_i$, the partial pressure of component $i$ and $q_i$, the adsorbed amount of component $i$ in mixture conditions determined by IAST predictions.

Langmuir parameters with evaluation of uncertainties with a 95% confidence level according to the method described by Tolazzi et al.[9]:

**Table S2**. Fitted Langmuir parameters and related uncertainties for $CO_2$ and $N_2$.

| | $CO_2$ | | | $N_2$ | | |
|---|---|---|---|---|---|---|
| | 298 K | 308 K | 318 K | 298 K | 308 K | 318 K |
| $q_s$ *(mmol/g)* | 4.099 +/- 0.111 | 3.921 +/- 0.102 | 3.734 +/- 0.092 | 2.975 +/- 0.034 | 2.914 +/- 0.026 | 2.862 +/- 0.019 |
| *b (1/bar)* | 7.307 +/- 0.820 | 5.037 +/- 0.456 | 3.732 +/- 0.275 | 0.133 +/- 0.006 | 0.106 +/- 0.003 | 0.086 +/- 0.002 |
| *RSS* | 0.0295 | 0.0145 | 0.0073 | 0.135 | 0.006 | 0.002 |

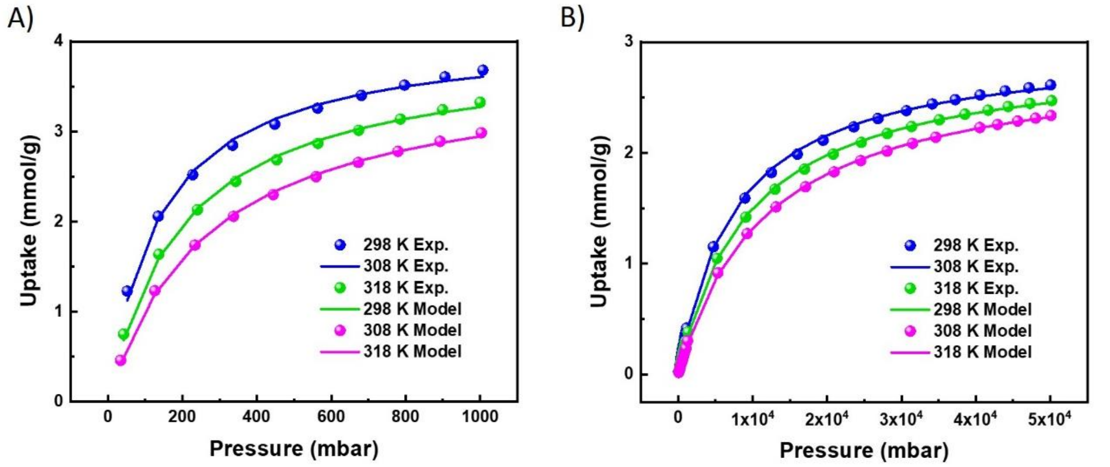


**Figure S2.** Langmuir plots for $CO_2$ and $N_2$ adsorption for MIL-120(Al)-AP. A) Comparison of experimental $CO_2$ isotherms with the isotherms calculated from the Langmuir model at three different temperatures, 298 K, 308 K, and 318 K. B) Comparison of experimental $N_2$ isotherms with the isotherms calculated from the Langmuir model at three different temperatures, 298 K, 308 K, and 318 K.

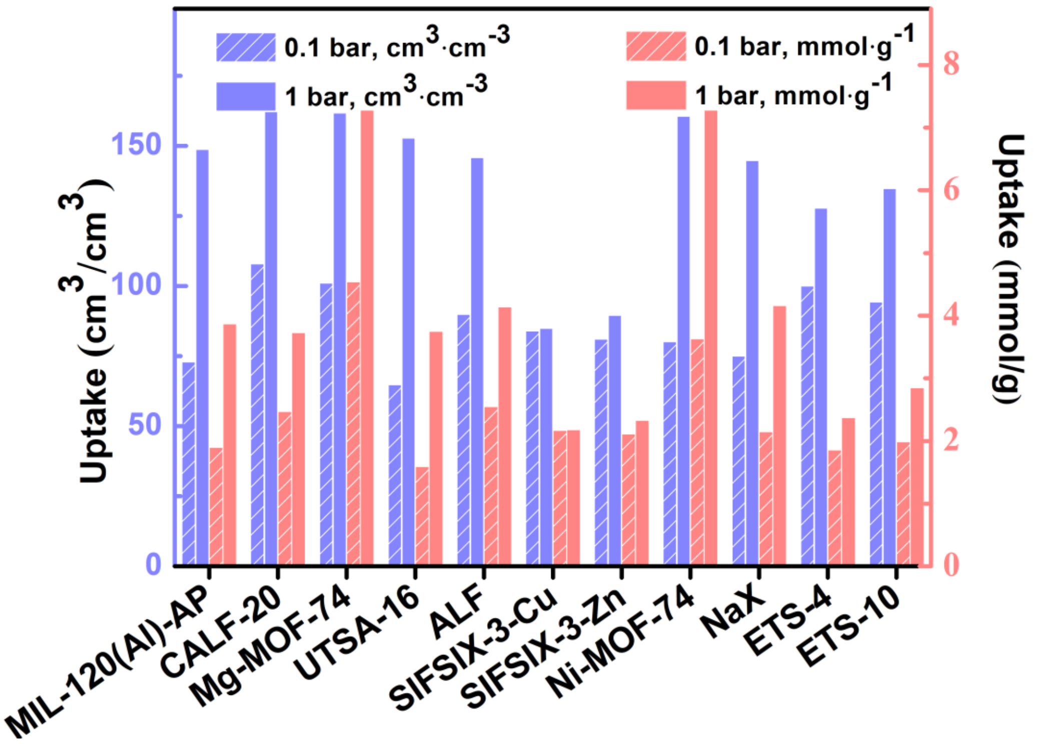


**Figure S3.** Comparison of volumetric and gravimetric $CO_2$ uptakes at 0.1, 1 bar, at 298 K between MIL-120(Al)-AP and benchmark adsorbents including MOFs and zeolites. The volumetric uptake was calculated using the crystallographic density.

**Table S3.** Selected promising adsorbents studied for $CO_2$ capture in VPSA cycles under simulated post-combustion conditions.

| $CO_2$ adsorbents | HOA for $CO_2$ (kJ/mol) | $CO_2$ capacity (mmol/g) at 100/200 mbar | $CO_2/N_2$ Selectivity | Reference |
|---|---|---|---|---|
| K-CHA | 24.6 | 3.8/4.0 (303 K) | 80[a] | [10] |
| Na-CHA | 39.4 | 4.2/4.4 (273 K) | 362-142[b] | |
| Ca-X | 53.3 | 2.0/- (323 K) | 250[c] | [11] |
| 13X | 42.5 | 2.6/3.3 (295 K) | 420[d] | [12] |
| Na-Y | 36.1 | 2.0/2.5 (295 K) | - | |
| CALF-20 | 39 | 2.3/2.8 (298 K) | 230[e] | [13] |
| UTSA-16 | 37 | 1.9/2.8 (298 K) | 171[e] | [14] |

[a]Si:Al=1.2, at 273 K; [b]Depending on the $Na^+$ exchange in CHA zeolite and ideal selectivity is defined as the ratio of Henry's law constants calculated from the Langmuir-Freundlich model at 298 K; [c]0.15 bar $CO_2$, 0.75 bar $N_2$ and 313 K; [d]$0.1CO_2/0.9N_2$ at 298 K; [e]10:90 $CO_2/N_2$ mixture.

## Chapter 4 Stability of MIL-120(Al)

### 4.1 Thermal stability

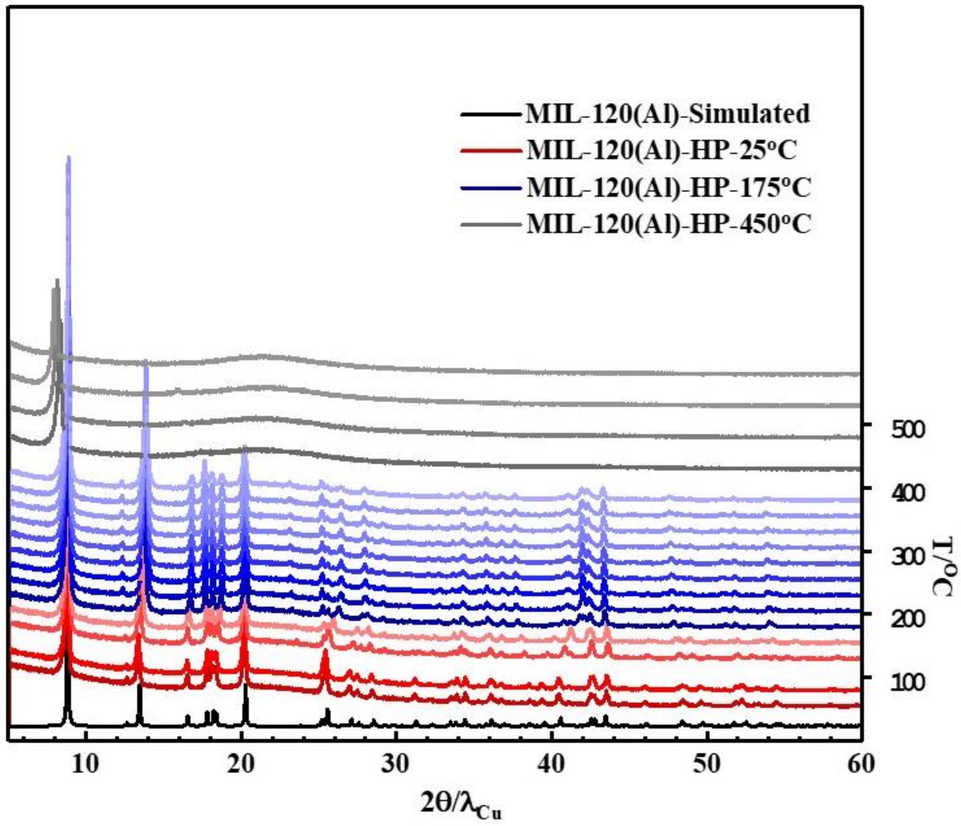


**Figure S4.** VT-PXRD of MIL-120(Al)-HP.

### 4.2 Water stability test

MIL-120(Al)-AP sample (200 mg) was dispersed into 50 mL DI water solution, then kept under reflux conditions for 10 days. White dry sample was collected after centrifugation and dried in air.

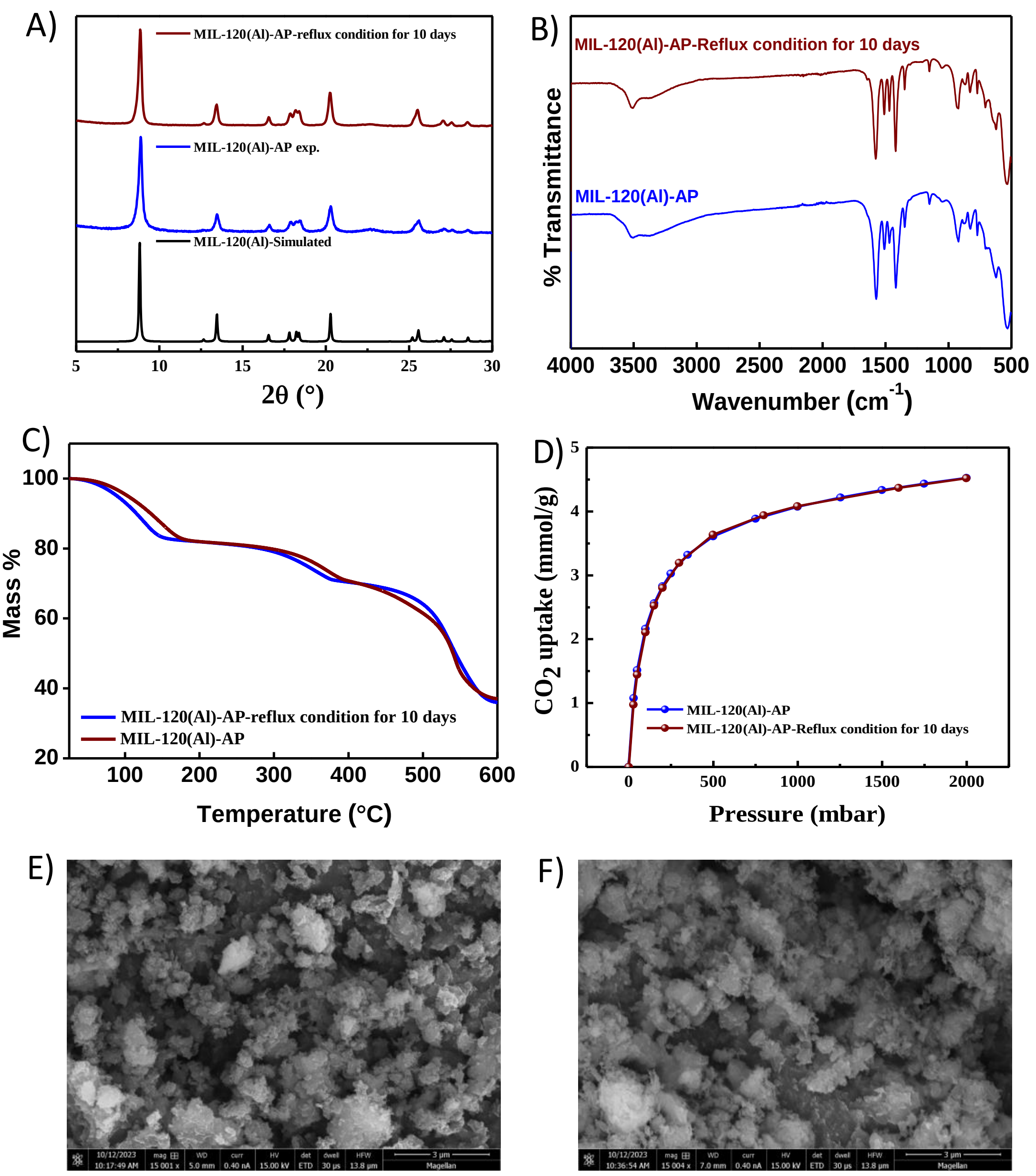


**Figure S5.** Water stability of MIL-120(Al)-AP. A) PXRD patterns (CuKα radiation (λ = 1.5418 Å)) showing the high crystallinity could be retained after 10 days' water reflux. B) Infra-red spectra showing the integrity of the MOF after the treatment. C) TGA curve of the MOF after treatment compared to the pristine MOF. D) $CO_2$ adsorption isotherm showing $CO_2$ uptake after 10 days' water reflux was highly consistent with that of the pristine MIL-120(Al)-AP sample. E) and F) are the SEM images of the MOF before and after treatment, respectively.

# Chapter 5 *In situ* temperature- and pressure- variable gas-loading synchrotron radiation powder diffraction (SRPD)

The *in situ* temperature- and pressure- variable SRPD data were collected at BM01 from the Swiss-Norwegian beamlines (SNBL) at the European Synchrotron Radiation Facility (ESRF, Grenoble in France). The wavelength of the experiment ($\lambda = 0.69668$ Å) was calibrated on $LaB_6$ standard using PyFAI.[15] The diffraction patterns were collected with multipurpose PULATUS@SNBL hybrid pixel 2D diffractometer and have been azimuthally integrated using Bubble.[16] The samples of MIL-120(Al)-HP and MIL-120(Al)-AP were loaded to 0.7 mm glass capillaries and connected to the gas-loading system. Prior to the gas loading ($CO_2$), the samples were kept under dynamic vacuum using a turbo-pump.

## 5.1 *In situ* SRPD of MIL-120(Al)-HP

### 5.1.1 *In situ* SRPD of MIL-120(Al)-HP under dynamic vacuum

The sample of MIL-120(Al)-HP was heated under dynamic vacuum using Oxford Cryostream 700+ from 298 to 400 K (6 K $min^{-1}$ heating rate), hold and cooled back (2.5 hours in total). The powder diffraction patterns were continuously collected every 10 seconds. The holding at 400 K continued until no visual changes of the powder diffraction patterns were observed. The sequential Le Bail refinement of the unit cell parameters using Fullprof [17] (Figure S6) suggests the irreversible change without any phase transition due to the water/solvent removal (up to ~370 K). The cooling of the activated sample gives the linear decrease of the unit cell parameters (due to thermal expansion, rather than solvent influence). The lower unit cell volume at 298 K after activation is also in line with this assumption (Figure S7). The crystal structure refinement using the Rietveld method (Figure S8) suggests the complete removal of water solvent from the crystal structure. The refinement was performed using the published *C*2/*m* crystal structure[5] without solvent molecules (water) with the following conditions:

a) The $B_{iso}$ are the same for all C and O atoms;
b) The coordinates of the framework's atoms were refined using soft distance

constraints:

*d*(Al...O) = 1.85 with sigma of 0.10 Å;

*d*(C...O) = 1.29 with sigma of 0.001 Å;

*d*(C-C) = 1.50 with sigma of 0.001 Å;

*d*(C=C) = 1.40 with sigma of 0.001 Å;

*d*(C=C=C) in benzyl ring = 2.80 with sigma of 0.001 Å;

c) The coordinates of the framework's atoms were refined using soft angle constraints:

angle(O-C-O) = 120 deg with sigma of 0.5 deg;

angle(O-C-C) = 120 deg with sigma of 0.5 deg;

angle(C-C-C) = 120 deg with sigma of 0.5 deg;

d) Slight preferred orientation along [0 2 0] was also refined (modified March's function) with a coefficient of 0.94413, where in the case of 1 there is no preferred orientation. Needle-like habit is expected for Debye-Scherrer geometry.

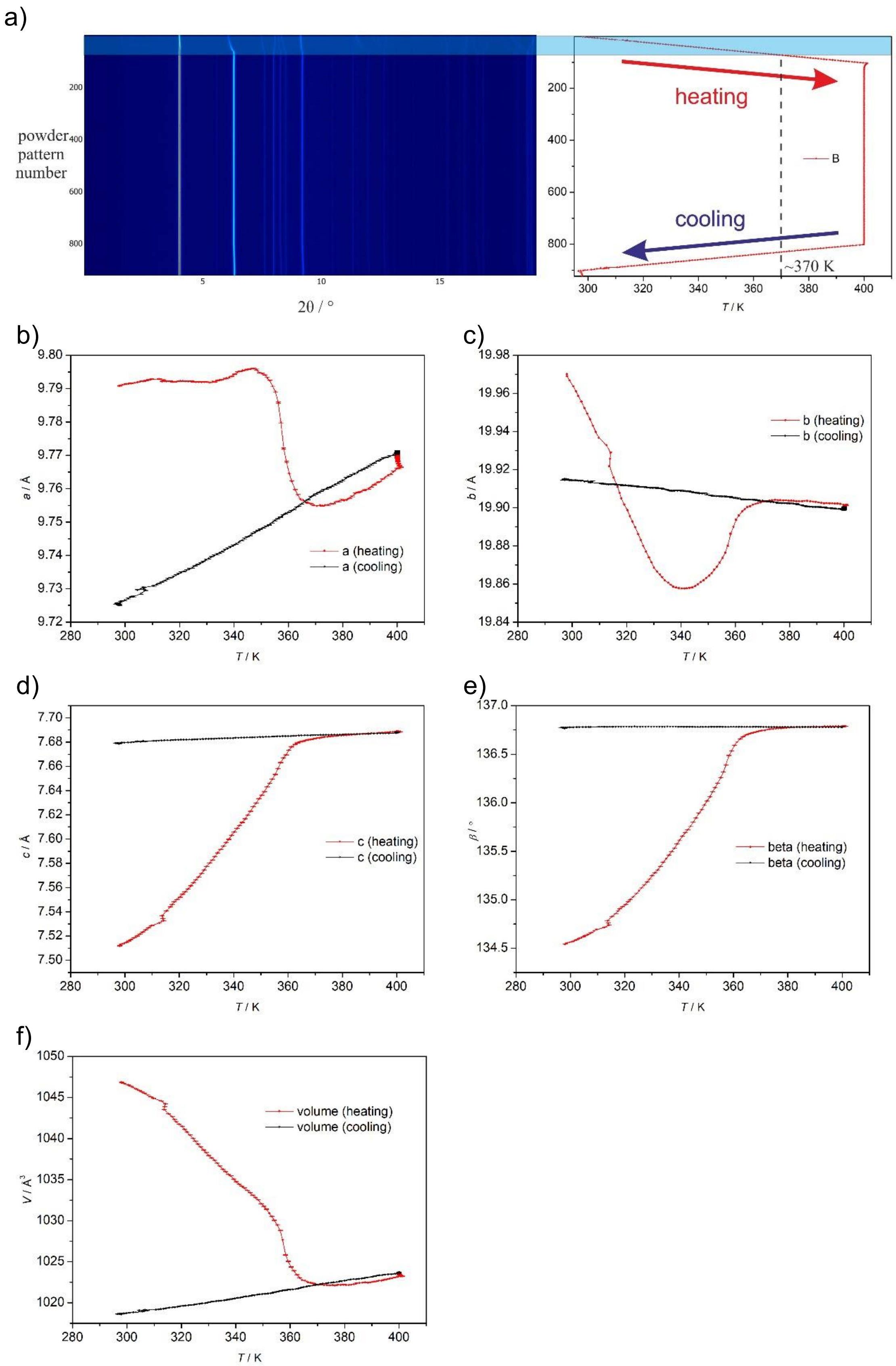
a)
powder pattern number
200
400
600
800
5
10
15
2θ / °
heating
cooling
B
~370 K
300
320
340
360
380
400
T / K
b)
a / Å
a (heating)
a (cooling)
T / K
c)
b / Å
b (heating)
b (cooling)
T / K
d)
c / Å
c (heating)
c (cooling)
T / K
e)
β / °
beta (heating)
beta (cooling)
T / K
f)
V / Å³
volume (heating)
volume (cooling)
T / K

**Figure S6.** a) Variable temperature SRPD of MIL-120(Al)-HP upon activation and b-f) the corresponding unit cell parameters dependences. Upon heating the unit cell parameters change dramatically until ~370 K (water removal), while after, their change is promoted mostly by the thermal expansion.

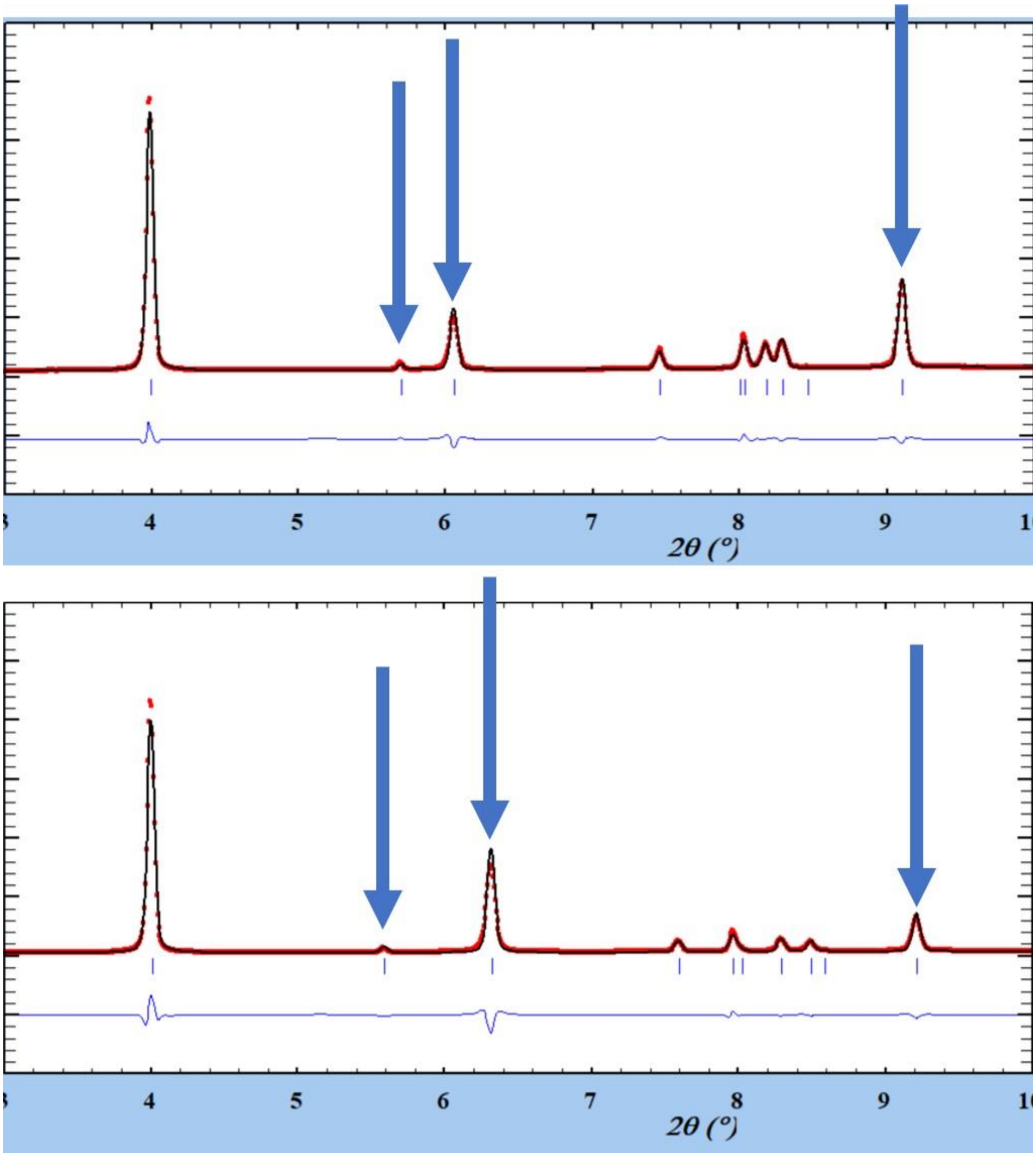


**Figure S7.** No structural transition occurred upon activation of the sample: according to the Le Bail fits of MIL-120(Al)-HP before (top: T = 298 K, space group *C*2/*m* with *a* = 9.7908 Å, *b* = 19.9702 Å, *c* = 7.5119 Å, *β* = 134.54°, *V* = 1046.9 $Å^3$) and after activation (bottom: T = 298 K, space group *C*2/*m* with *a* = 9.7205(2) Å, *b* = 19.9102(4) Å, *c* = 7.6750(2) Å, *β* = 136.7599(12)°, *V* = 1017.59(4) $Å^3$), only the change of the unit cell parameters due to the removal of water molecules from the pores is observed.

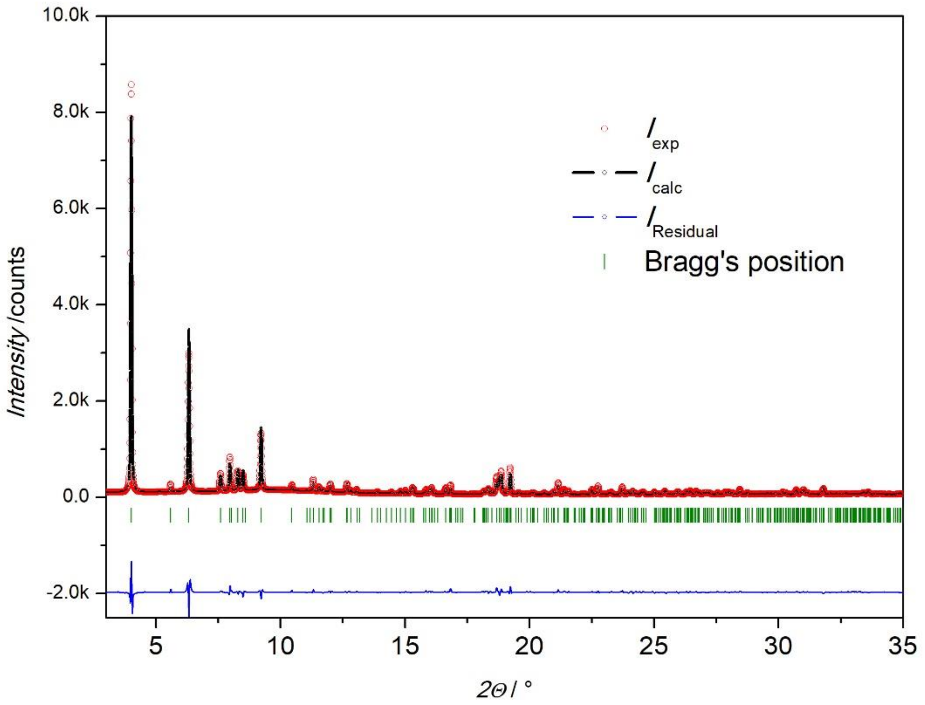


**Figure S8.** Rietveld plot of the MIL-120(Al)-HP crystal structure refinement (298 K) after activation (space group *C*2/*m*, $R_{I\ (Bragg)}$ = 6.02 %, $R_f$ = 5.52 %: *a* = 9.7205(2) Å, *b* = 19.9102(4) Å, *c* = 7.6750(2) Å, *β* = 136.7599(12)°, *V* = 1017.59(4) Å$^3$).

### 5.1.2 *In situ* SRPD of MIL-120(Al)-HP after $CO_2$-loading

The $CO_2$-loading to the activated sample (298 K) was performed stepwise with 100, 200, 300, 400, 1000, 1500, 2000, 3000 and 4000 mbar. The powder diffraction patterns have been measured several times at the same pressure step until no change of the diffraction pattern was observed (for the equilibrium). The phase transition from the monoclinic *C*2/*m* to triclinic *P*-1 was observed at 4000 mbar pressure. The crystal structure of the *C*2/*m* model was transformed to *P*-1 using the PowderCell program by group-subgroup relations.[18] The direct space methods in FOX[19] confirmed the presence of $CO_2$ molecules in the pores and the crystal structure refinement conditions were similar to the activated sample:

a) The $B_{iso}$ are the same for all C and O atoms of the framework and for the $CO_2$ molecule (another parameter);
b) The coordinates of the framework's atoms were refined using soft distance constraints:
   *d*(Al...O) = 1.85 with sigma of 0.10 Å;
   *d*(C...O) = 1.29 with sigma of 0.001 Å;

$d$(C-C) = 1.50 with sigma of 0.001 Å;

$d$(C=C) = 1.40 with sigma of 0.001 Å;

$d$(O=C=O) = 1.20 with sigma of 0.001 Å for the $CO_2$ molecule;

$d$(C=C=C) in benzyl ring = 2.80 with sigma of 0.001 Å;

c) The coordinates of the framework's atoms were refined using soft angle constraints:

angle(O-C-O) = 120 deg with sigma of 0.5 deg;

angle(O-C-C) = 120 deg with sigma of 0.5 deg;

angle(C-C-C) = 120 deg with sigma of 0.5 deg;

d) The $x$, $y$, $z$ coordinates of C and O of the $CO_2$ molecule were refined together;

e) The preferred orientation along [1 -1 0] was fixed and taken from the activated $C2/m$ sample (where it was applied to [0 2 0] with 0.94413 coefficient using modified March's function).

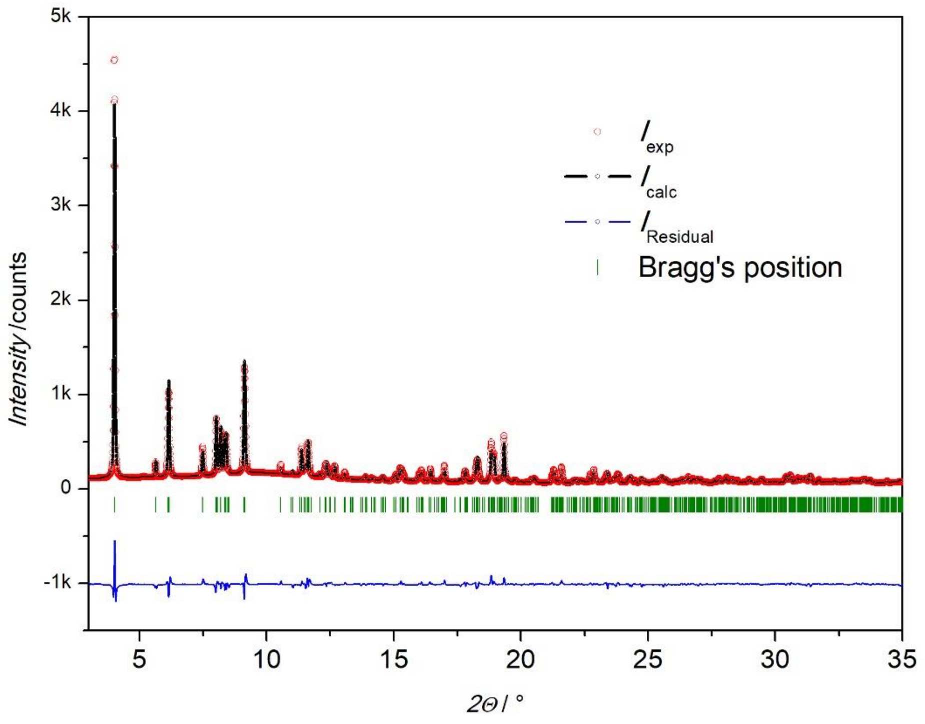


**Figure S9.** Rietveld plot of the MIL-120(Al)-HP crystal structure refinement (298 K) after 4000 mbar of $CO_2$-loading (space group $P$-1, $R_{I\ (Bragg)}$ = 8.50 %, $R_f$ = 7.44 %: $a$ = 11.0773(2) Å, $b$ = 11.0382(2) Å, $c$ = 7.6003(2) Å, $\alpha$ = 108.419(2)°, $\beta$ = 108.326(2)°, $\gamma$ = 127.4723(16)°, $V$ = 517.67(2) Å$^3$).

In order to increase the $CO_2$-loading, a low temperature (200 K) experiment was performed with stepwise pressure increase of 100, 200, 300, 400, 500 and 1000 mbar.

The powder diffraction patterns have been measured several times at the same pressure step until no change of the diffraction pattern was observed (for the equilibrium). The refinement conditions were the same as for the 298 K data with $CO_2$, except that one more molecule was included in the refinement. The coordinate of its C6 was fixed to 0 0 ½ and the occupancy for its $CO_2$ molecule was fixed to ½.

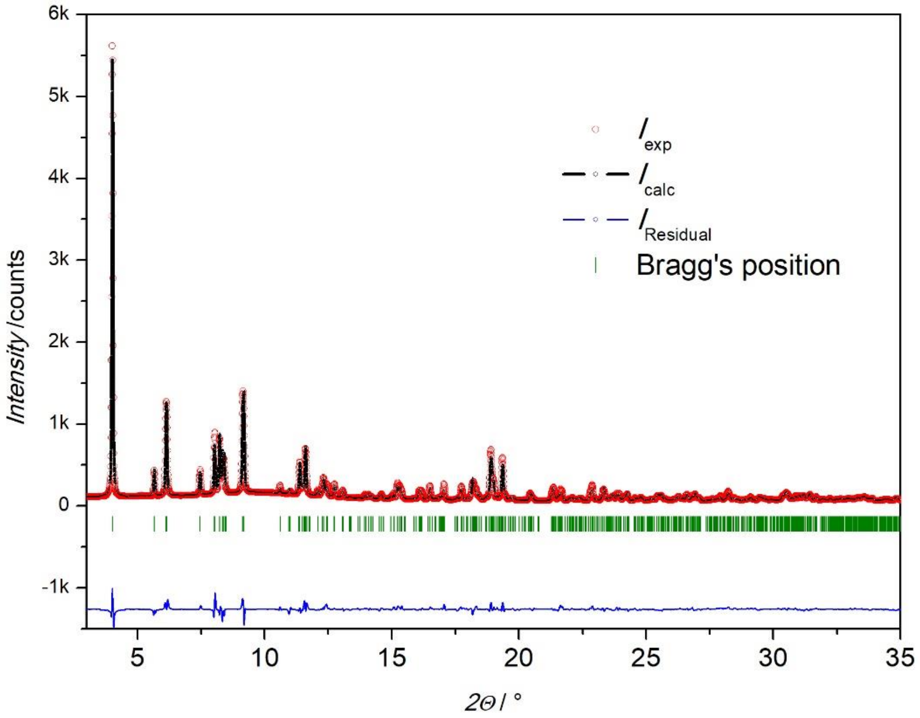


**Figure S10.** Rietveld plot of MIL-120(Al)-HP crystal structure refinement (200 K) after 1000 mbar of $CO_2$-loading (space group *P*-1, $R_{I\ (Bragg)}$ = 9.94 %, $R_f$ = 6.88 %: *a* = 11.0773(2) Å, *b* = 11.0277(3) Å, *c* = 7.5710(3) Å, *α* = 108.214(3)°, *β* = 108.155(3)°, *γ* = 127.6722(19)°, *V* = 517.21(3) Å$^3$).

## 5.2 *In situ* SRPD of MIL-120(Al)-AP

### 5.2.1 *In situ* SRPD of MIL-120(Al)-AP under dynamic vacuum

Taking into account the behavior of the MIL-120(Al)-HP sample, where the change occurred around 370 K, the MIL-120(Al)-AP sample was heated using Oxford Cryostream 700+ from 293 to 373 K (6 K min$^{-1}$ heating rate) under dynamic vacuum, hold and then cooled back (1 hour in total). Contrary to the MIL-120(Al)-HP sample, the splitting of some powder diffraction peaks has been observed (Figure S12). This transformation was irreversible and could be described as *C*2/m to *P*-1 transition. The

crystal structure refinement in *P*-1 by the Rietveld method was performed using the same model as MIL-120(Al)-HP polymorph without $CO_2$ molecules with the same constraints (Figure S13). The preferred orientation along [1 -1 0] was refined as 0.94924 coefficient using modified March's function.

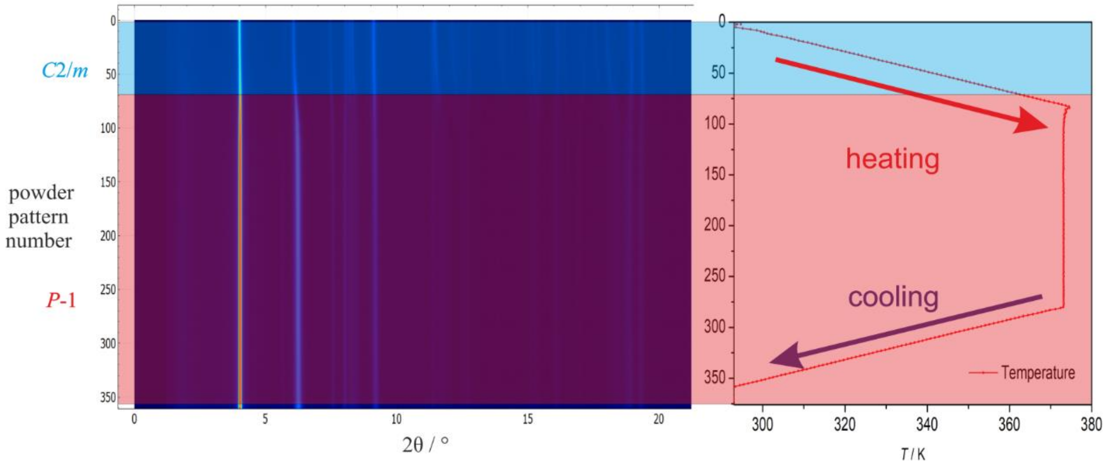


**Figure S11.** Variable temperature SRPD of MIL-120(Al)-AP upon activation. The irreversible phase transition from *C*2/m to *P*-1 is observed under dynamic vacuum.

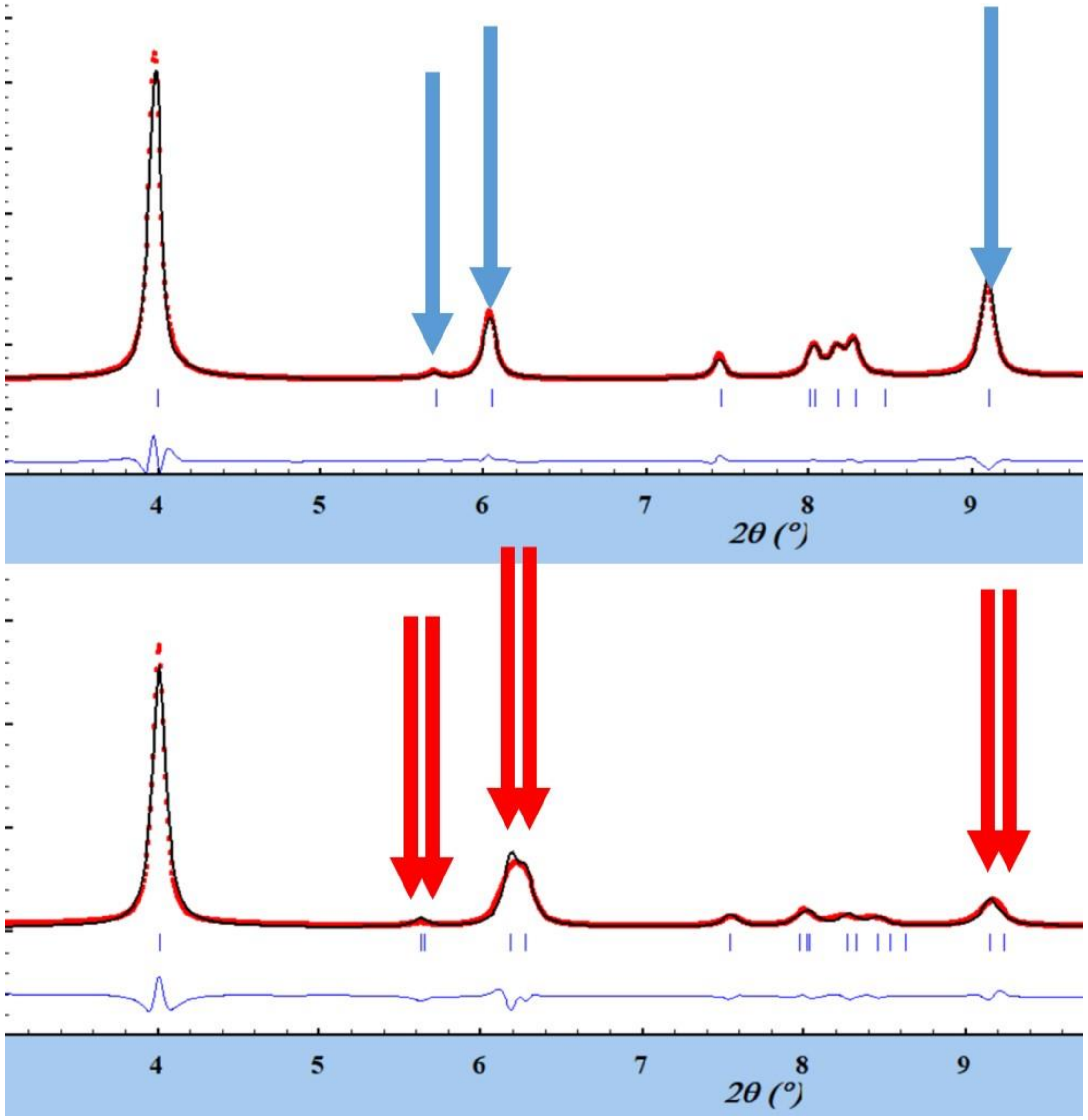


**Figure S12.** The peaks change of MIL-120(Al)-AP before (top) and after activation (bottom).

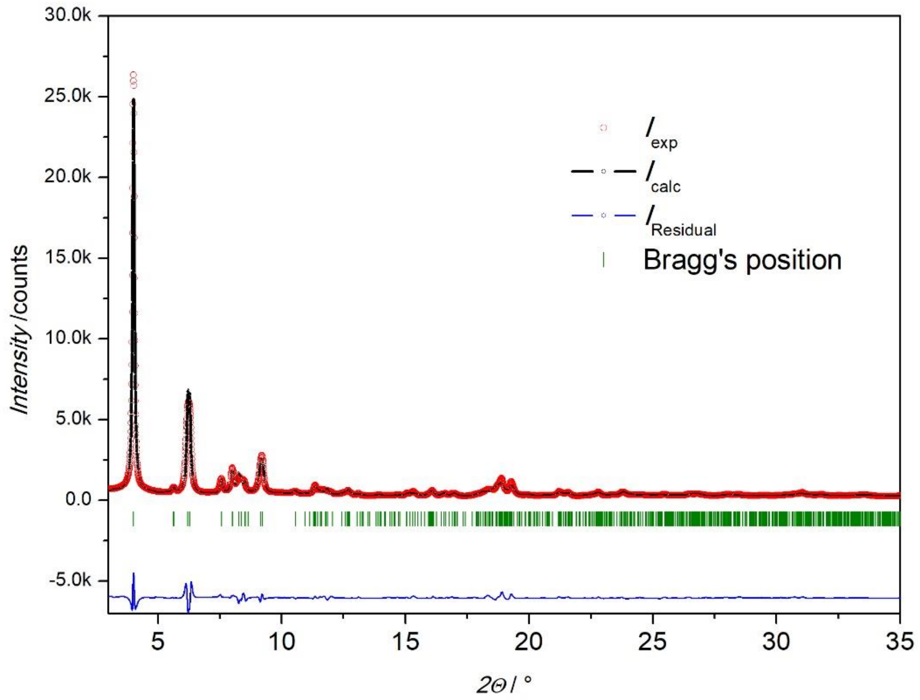


**Figure S13.** Rietveld plot of the MIL-120(Al)-AP crystal structure refinement (298 K) after activation (space group *P*-1, $R_{\mathrm{I\,(Bragg)}}$ = 5.28 %, $R_{\mathrm{f}}$ = 4.47 %: *a* = 11.1134(19) Å, *b* = 11.0236(20) Å, *c* = 7.6018(12) Å, α = 108.797(18)°, β = 108.143(18)°, γ = 127.823(8)°, *V* = 510.19(15) Å$^3$).

### 5.2.2 *In situ* SRPD of MIL-120(Al)-AP after $CO_2$-loading

The low temperature (200 K) experiment with stepwise pressure increase of 100, 200, 300, 400, 500, 750 and 1000 mbar suggested the best fit using the *C*2/*m* crystal structure model, see Figure S14. The crystal structure refinement was performed using two independent $CO_2$ molecules and the same interatomic soft distance/angle constraints of the previous refinements. The position of the C6 was fixed to 0 0 ½ (2*c*) and the occupancy was full (as well as O6). The occupancy of another $CO_2$ molecule was refined to be 0.823. The preferred orientation along [0 2 0] was refined as 0.89315 using modified March's function.

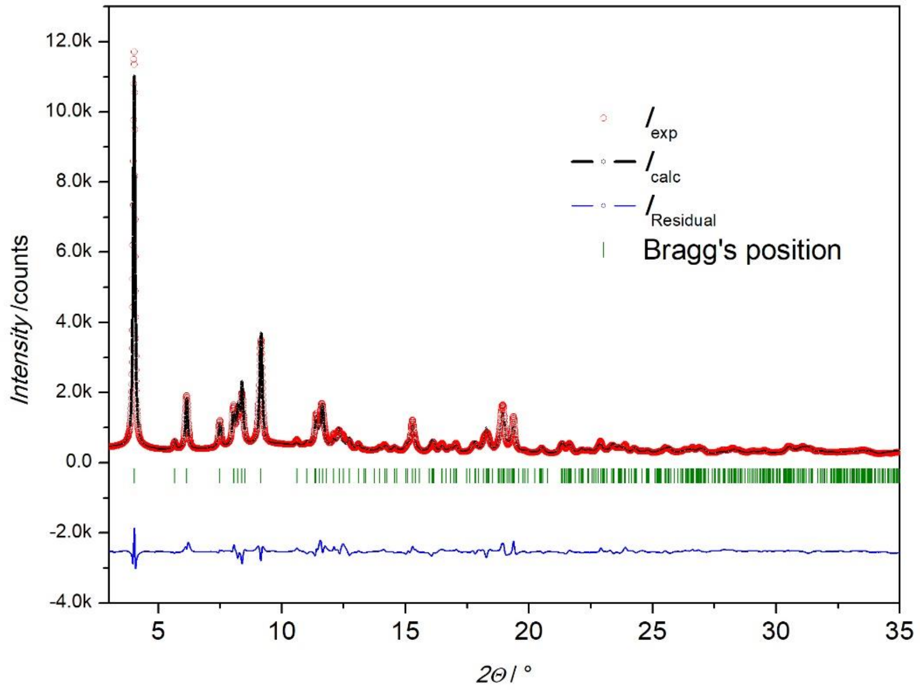


**Figure S14.** Rietveld plot of the MIL-120(Al)-AP crystal structure refinement (200 K) after 1000 mbar of $CO_2$-loading ($C2/m$, $R_{I\ (Bragg)}$ = 8.45 %, $R_f$ = 5.81 %: $a$ = 9.7589(7) Å, $b$ = 19.8260(12) Å, $c$ = 7.5685(8) Å, β = 135.254(5)°, $V$ = 1030.85(15) Å$^3$).

# Chapter 6 Simulation and modeling

## 6.1 Computational methods

The structural geometry optimizations and total energy calculations of MIL-120(Al) were carried out with the DFT method as implemented in the Vienna Ab initio Simulation Package (VASP, version 5.4.4).[20] The projector augmented wave (PAW) method was applied to describe the electron-ion interaction.[21] The exchange-correlation interaction functional is the generalized gradient approximation (GGA) in the Perdew-Burke-Ernzerhof (PBE) functional[22] and the electron wave functions are expanded using the plane waves with an energy cutoff of 650 eV. The convergence of total energy for the self-consistent wave function and the force between atoms for geometrical optimization are set to $10^{-5}$ eV and 0.01 eV/Å, respectively. The DFT-D3 method of the Grimme van der Waals correction approach[23] was also used to accurately account for the long-range interactions between $CO_2$ molecules and the MIL-120(Al) structure. The Brillouin zone was sampled with a 5 × 5 × 6 Monkhorst-Pack k-points grid.[24]

We employed grand canonical Monte Carlo (GCMC) to calculate adsorption isotherms for $CO_2$ in the MIL-120(Al) structure. The interactions between the host and guest molecules were considered by combining Coulombic and 12-6 Lennard-Jones (LJ) contributions. Electrostatic interactions were computed using Ewald summation with a precision of $10^{-6}$. The LJ interaction cutoff was set at 12.0 Å. To achieve equilibrium, each data point underwent $1 \times 10^7$ Monte Carlo cycles, preceded by $2 \times 10^7$ equilibration cycles, by using the Complex Adsorption and Diffusion Simulation Suite (CADSS) code.[25] The universal force field (UFF) force field parameters[26] were adopted to describe the LJ parameters for the MIL-120(Al) framework. The $CO_2$ molecule was modeled by using the EPM2 model,[27] which involves three atom-centered charged LJ sites. The $N_2$ molecule was described by the TraPPE model, corresponding to three-site model.[28] The LJ cross parameters were determined using Lorentz-Berthelot mixing rules.[29] The adsorption enthalpies were determined using the revised Widom's test particle insertion method.[30]

All the force-field parameters of the MOF and molecules ($CO_2$ and $N_2$) can be found in the Table S6.

**Table S4.** Comparison of the simulated cell parameters for MIL-120(Al)-AP-Str1 and MIL-120(Al)-AP-Str2.

| **MIL-120(Al)-AP** | ***a*** (Å) | ***b*** (Å) | ***c*** (Å) | ***α*** (deg.) | ***β*** (deg.) | ***γ*** (deg.) | **V** (Å$^3$) |
|---|---|---|---|---|---|---|---|
| Exp. [dry] | 11.1134 | 11.024 | 7.6018 | 108.7970 | 108.1430 | 127.8230 | 510.2 |
| Str1 [PBE-D3] | 11.103 | 11.142 | 7.581 | 108.092 | 107.637 | 128.777 | 515.105 |
| Str2 [PBE-D3] | 11.100 | 11.083 | 7.500 | 107.644 | 107.555 | 127.914 | 527.776 |

**Table S5.** Universal force-field (UFF)[26] parameters for MIL-120(Al)-AP-Str1 and MIL-120(Al)-AP-Str2 used in the GCMC simulations.

| **Lennard-Jones Parameters** | | |
|---|---|---|
| **Atom** | **ε (kcal mol$^{-1}$)** | **σ (Å)** |
| **Al** | 0.000 | 4.0082 |
| **O** | 0.060 | 3.1181 |
| **C** | 0.105 | 3.4309 |
| **H$_{(linker)}$** | 0.044 | 2.5711 |
| **H$_{(\mu2\text{-}OH)}$** | 0.000 | 2.5711 |

**Table S6.** Force-field parameters for the $CO_2$[27] and $N_2$ molecules.[28]

| **$CO_2$** | | | |
|---|---|---|---|
| **Atom** | **ε (kcal mol$^{-1}$)** | **σ (Å)** | **Charge** |
| C | 0.05590 | 2.757 | 0.65120 |
| O | 0.15998 | 3.033 | -0.3256 |
| **$N_2$** | | | |
| N | 0.0715 | 3.31 | -0.482 |
| Pseudo atom | 0.000 | 0.000 | 0.964 |

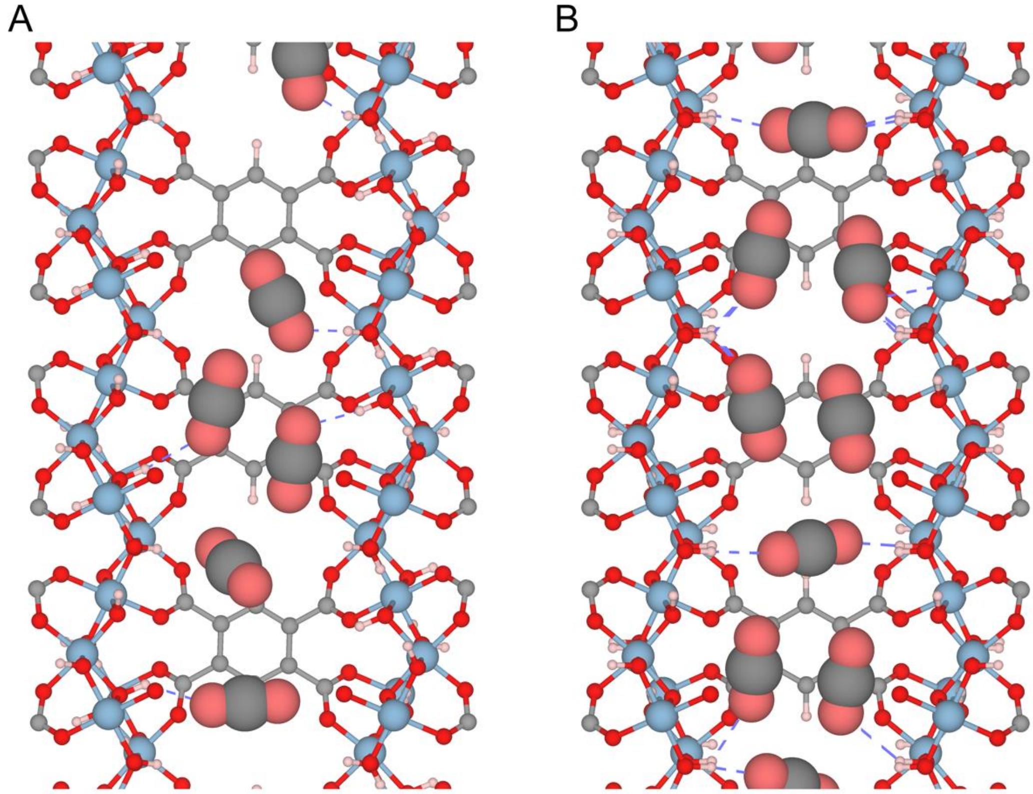


**Figure S15**. The representative snapshots for $CO_2$-loaded (A) MIL-120(Al)-AP-Str1 and (B) MIL-120(Al)-AP-Str1 channels at 1 bar and 298 K, calculated using the GCMC method. The blue dashed line represents interactions between $CO_2$ molecule and $\mu_2$-OH group of MOFs. Color code Al; light blue, C, gray; O, red; H, light pink.

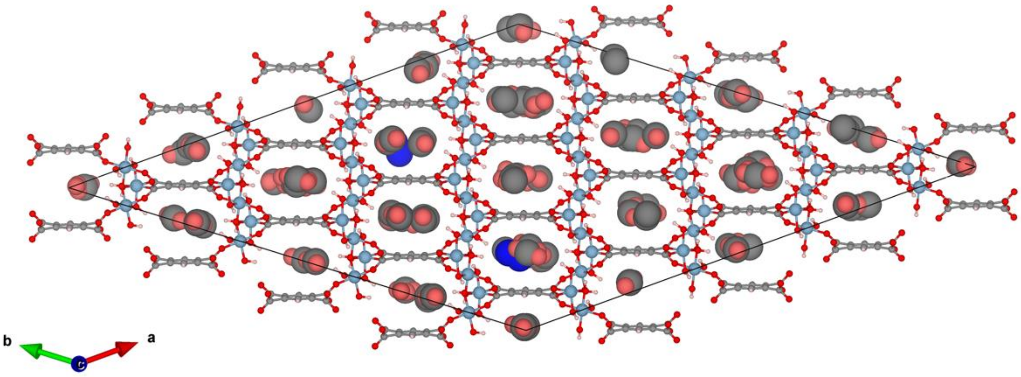


**Figure S16**. The snapshot showing the location of $15CO_2$:$85N_2$ in MIL-120(Al)-AP-Str1 at 1 bar and 298 K from GCMC simulation. Color code Al; light blue, C, gray; O, red; H, light pink; N, blue.

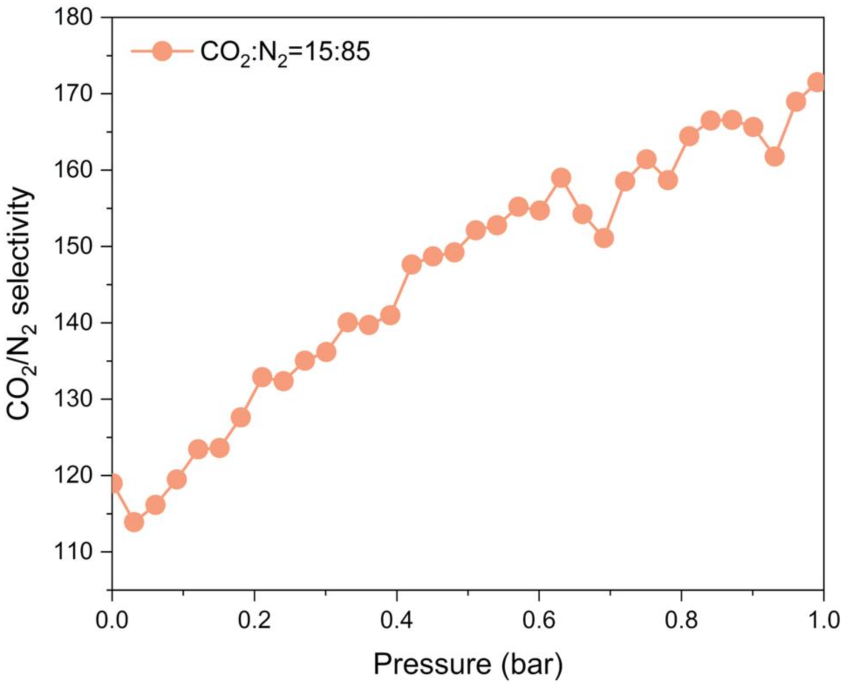


**Figure S17.** The GCMC simulated $15CO_2{:}85N_2$ co-adsorption thermodynamic selectivity at 298 K as a function of pressure.

**Table S7.** Six times $CO_2$ adsorption measurements of MIL-120(Al)-AP.

| | **Before exposure to air** | | | **After exposure to air** | | |
|---|---|---|---|---|---|---|
| **Act. Temp.** | **1st activation at 25 °C** | **2nd activation at 25 °C** | **3rd activation at 50 °C** | **1st activation at 25 °C** | **2nd activation at 25 °C** | **3rd activation at 50 °C** |
| Act. Time (hour) | 6 | 6 | 6 | 6 | 6 | 6 |
| Sample weight (mg) | 78.54 | 78.44 | 78.02 | 78.50 | 78.48 | 78.00 |
| $CO_2$ uptake (mmol $g^{-1}$) at 0.15 bar | 2.55 | 2.52 | 2.26 | 2.32 | 2.30 | 2.26 |

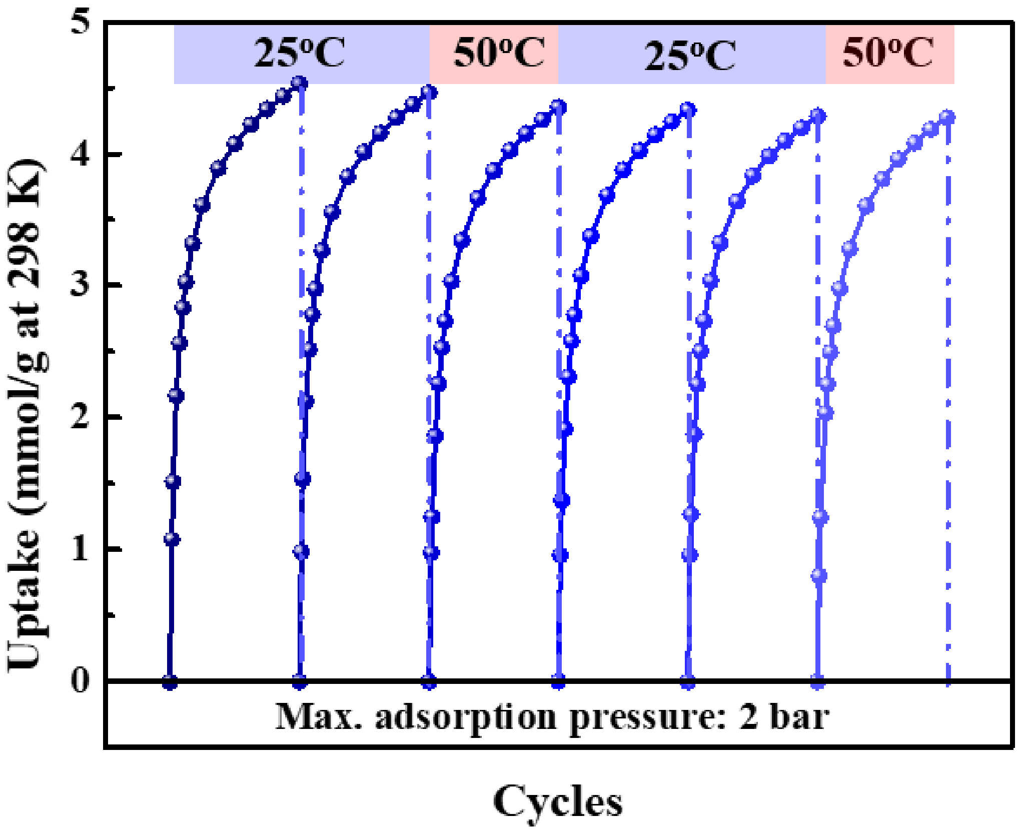


**Figure S18.** Six times $CO_2$ adsorption measurements of MIL-120(Al)-AP.

## Chapter 8 Synthesis optimization of MIL-120(Al)-AP in large-scale

**Table S8.** The price of different aluminum precursors from Sigma supplier.

| Aluminum precursor | Quantity (kg) | Price (euro) |
|---|---|---|
| $NaAlO_2$ | 2.5 | 81.8 |
| $Al_2(SO_4)_3\,xH_2O$ | 2.5 | 119 |
| $Al(OH)_3$ | 2.5 | 156 |
| $Al(OH)(C_2H_3O_2)_2$ | 2.5 | 214 |
| $Al_2(SO_4)_3\,18H_2O$ | 2.5 | 263 |
| $Al(NO_3)_3\,9H_2O$ | 2.5 | 294 |
| $AlCl_3\,6H_2O$ | 2.5 | 298 |

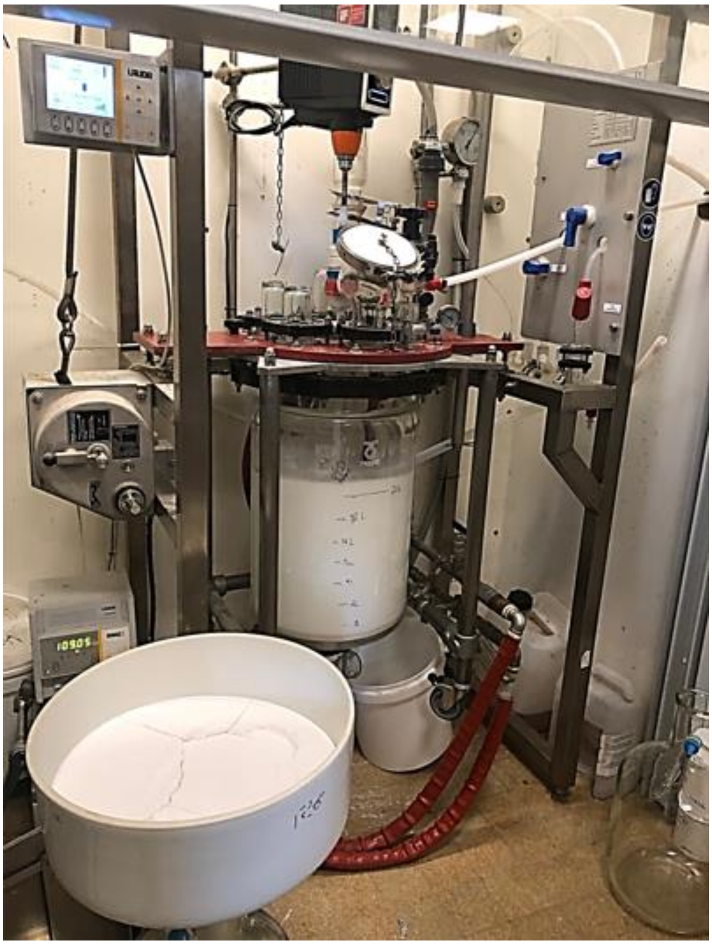

**Figure S19.** 30 L reactor used for the large-scale synthesis of MIL-120(Al)-AP in the lab with filtration part.

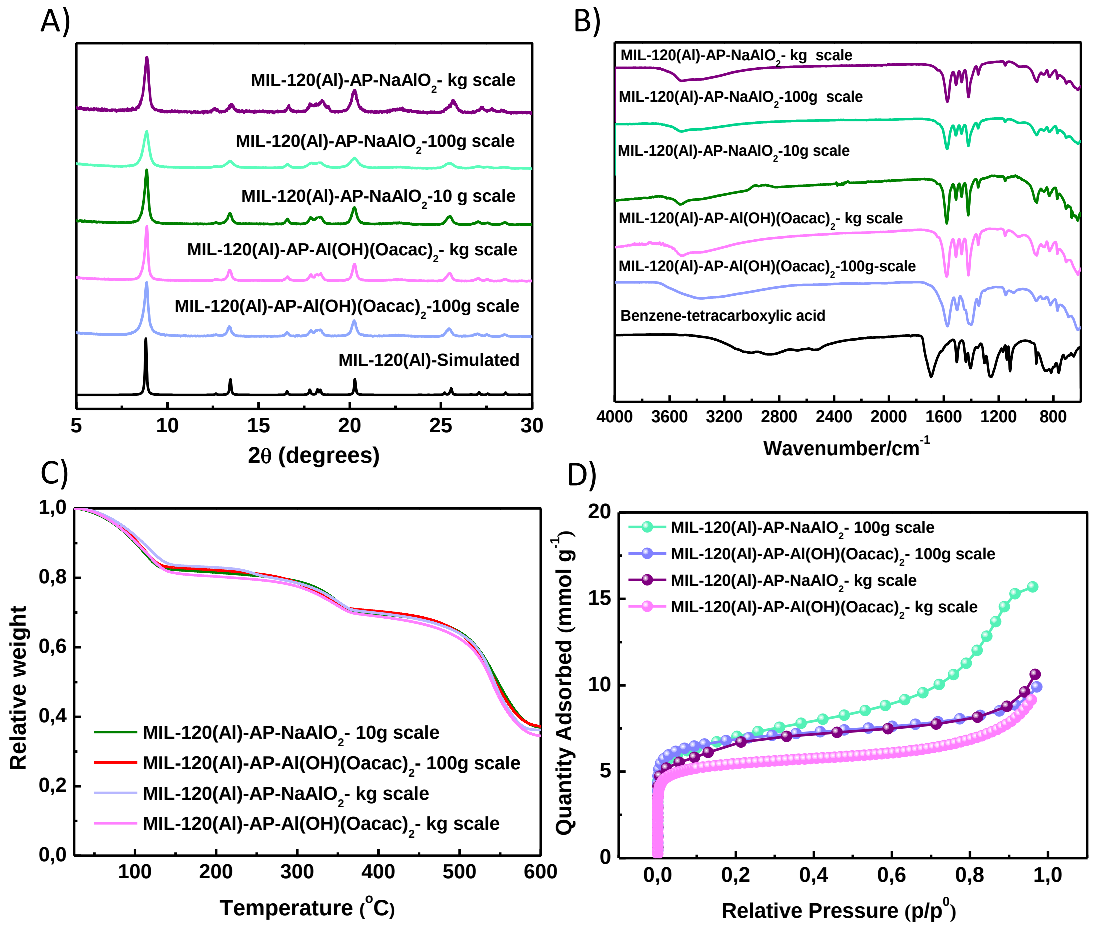


**Figure S20.** Characterizations of the different synthesis scale of MIL-120(Al)-AP. A) PXRD patterns (CuKα radiation (λ = 1.5418 Å)) showing that pure MIL-120(Al)-AP phase can be obtained. B) FT-IR spectra showing that there is no free carboxylic group in the different MIL-120(Al)-AP samples. C) TGA results showing similar composition between the different MIL-120(Al)-AP. D) $N_2$ adsorption isotherms showing similar BET surface areas (471 $m^2$ $g^{-1}$ for MIL-120(Al)-AP-Al(OH)($CH_3COO$)$_2$-1kg; 527 $m^2$ $g^{-1}$ for MIL-120(Al)-AP-$NaAlO_2$-100g); the slight difference might be caused by the different activation conditions under vacuum.

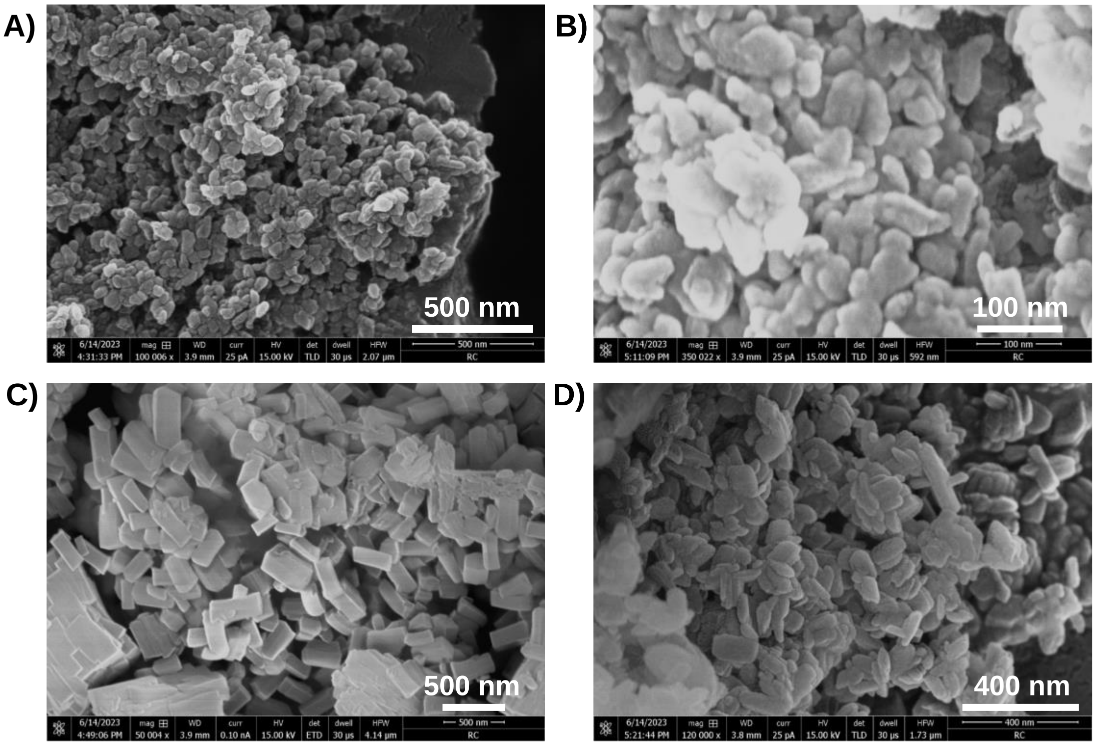


**Figure S21.** SEM images of the different synthesis scale of MIL-120(Al)-AP. A) Small scale synthesis using $Al(OH)(CH_3COO)_2$ showing a particle size around 50-70 nm. B) Small scale synthesis using $NaAlO_2$ showing a particle size around 30-40 nm. C) Large scale synthesis using $Al(OH)(CH_3COO)_2$ showing a particle size around 300 nm. D) Large scale synthesis using $NaAlO_2$ showing a particle size around 40-50 nm.

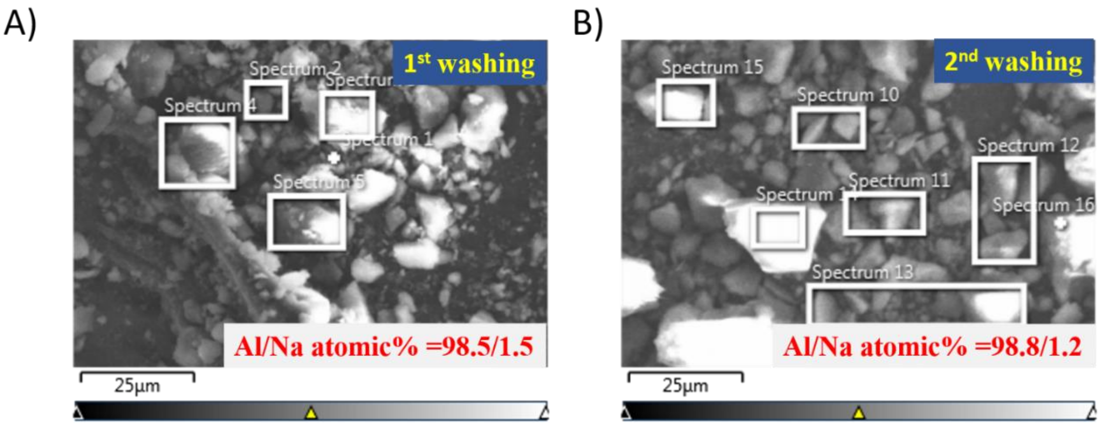


**Figure S22.** EDX results of sodium element in the MIL-120(Al)-AP-100g scale. A) First time washing, B) Second time washing in water at 60 °C for 24 hours, showing there existed around 1.5% of sodium element in the sample.

## Chapter 9 Solid state NMR studies

### 9.1 Solid state NMR studies

$^{1}H$ and $^{27}Al$ spectra were acquire on a 17.6T Bruker NEO spectrometer operating at 750.0 MHz and 195.4 MHz respectively, spinning the sample at 60 kHz in 1.3 mm diameter zirconia rotors. $^{1}H$ spectra were acquired using a Hahn echo sequence with an inter-pulse delay of 167 $\mu$s (10 rotor periods), a recycle delay of 0.7 s and a radio-frequency field of 130 kHz. Quantitative $^{27}Al$ spectra were obtained using a Bloch-decay sequence with an rf-field of 50 kHz, a short pulse of 0.33 $\mu$s (*i.e.*, less than $\pi/18$) and a recycle delay of 0.7 s. $^{27}Al$ MQMAS experiments were performed with a Z-filter sequence[31] using excitation and reconversion pulses of 1.5 $\mu$s and 0.53 $\mu$s respectively at rf-fields of 200 kHz, a selective $\pi/2$ pulse of 8.3 $\mu$s at 10 kHz rf-field, a Z-filter of 3 rotor periods (50 $\mu$s) and a recycle delay of 0.7 s. $t_1$ increment in the indirect dimension was 33.33 $\mu$s (30 kHz spectral width) with 160 slices acquired with a States procedure. $^{13}C$ and $^{1}H$ spectra were acquired using variable amplitude cross-polarization on a 9.0 T Bruker Avance III spectrometer operating at Larmor frequencies of 100.6 MHz and 400.2 MHz. Samples were packed in 4 mm diameter zirconia rotors and spun at 10 kHz. For cross-polarization, a 500 $\mu$s contact time was used with an rf-field of 25 kHz on $^{13}C$ and 35 kHz on $^{1}H$ and a 90% to 110% ramp through the Hartman-Hahn condition. Recycle delay was set to 1.0 s and $^{1}H$ decoupling was performed with the spinal64 scheme at an rf-field of 75 kHz.

$^{1}H$ and $^{13}C$ spectra are reference with respect to TMS and $^{27}Al$ with respect to a 1 M aqueous solution of $Al(NO_3)_3$. All spectra are simulated with the DMFit software.[32]

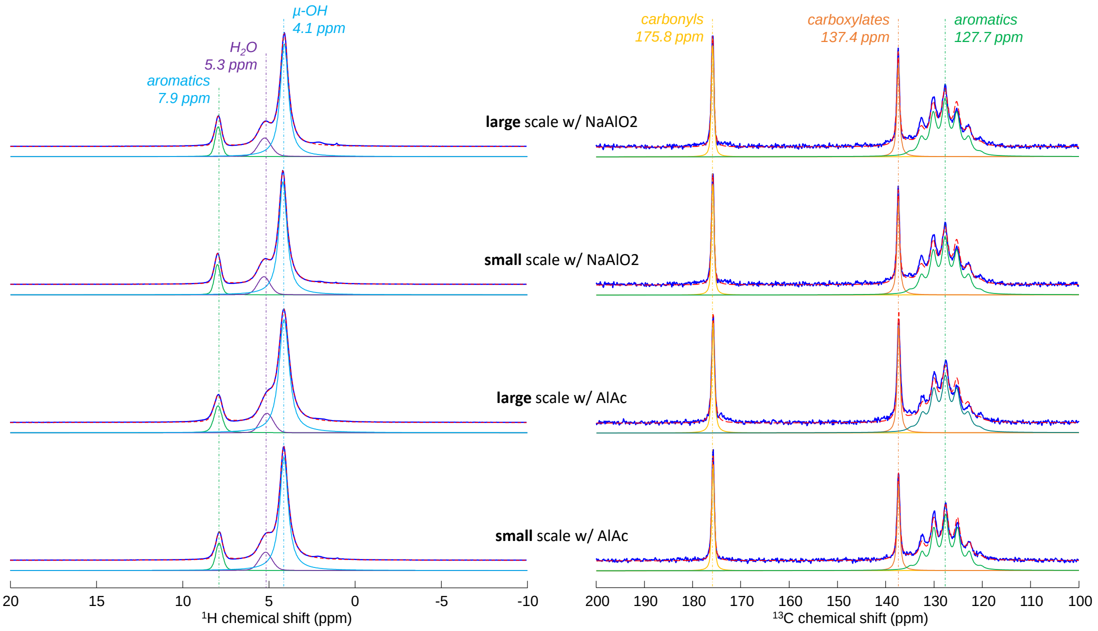


**Figure S23.** Experimental (dark blue) and simulated (dashed red) $^{1}H$ (left) and $^{13}C$ (right) ssNMR MAS spectra for all compositions studied here. Individual components of the simulations are given below each spectra.

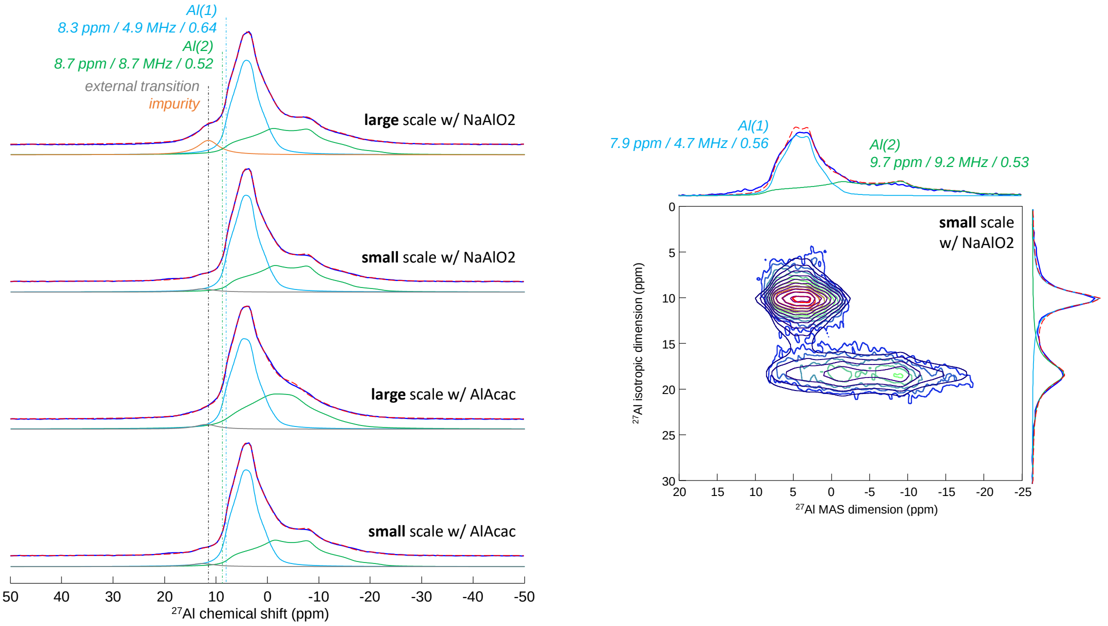


**Figure S24.** Left: experimental (dark blue) and simulated (dashed red) $^{27}Al$ ssNMR MAS spectra for all compositions studied here, with individual components of the simulations given below each spectra. Right: $^{27}Al$ 2D MQMAS spectra (colored contour lines) and simulation (dark purple contour lines) of the small scale $NaAlO_2$ synthesis, along with projections onto the two dimensions showing also the individual components used for the simulation.

**Table S9.** $^{1}H$ and $^{13}C$ NMR parameters extracted from the simulations using Voigt functions: population (%), isotropic chemical shift ($\delta_{iso}$, ppm), full width at half maximum ($\Delta\delta_{iso}$, ppm) and Gaussian/Lorentzian ratio. "SS" stands for "small scale" and "LS" for "large scale".

| $^{1}H$ NMR | H-aromatics | | | | $H_2O$ | | | | μ-OH | | | |
|---|---|---|---|---|---|---|---|---|---|---|---|---|
| Compound | % | $\delta_{iso}$ | $\Delta\delta_{iso}$ | G/L | % | $\delta_{iso}$ | $\Delta\delta_{iso}$ | G/L | % | $\delta_{iso}$ | $\Delta\delta_{iso}$ | G/L |
| SS $NaAlO_2$ | 11.9 | 8.0 | 0.43 | 0.71 | 13.3 | 5.3 | 0.85 | 0.86 | 74.8 | 4.2 | 0.57 | 0.03 |
| LS $NaAlO_2$ | 11.7 | 7.9 | 0.43 | 0.73 | 14.9 | 5.3 | 0.90 | 0.75 | 73.4 | 4.1 | 0.57 | 0.07 |
| SS AlAcac | 11.5 | 7.9 | 0.47 | 0.67 | 13.2 | 5.2 | 0.88 | 0.86 | 75.3 | 4.1 | 0.59 | 0.02 |
| LS AlAcac | 12.0 | 7.9 | 0.56 | 0.61 | 11.8 | 5.1 | 0.88 | 0.96 | 76.1 | 4.1 | 0.71 | 0.14 |
| $^{13}C$ NMR | Carbonyls | | | | Carboxylates | | | | Aromatics | | | |
| Compound | % | $\delta_{iso}$ | $\Delta\delta_{iso}$ | G/L | % | $\delta_{iso}$ | $\Delta\delta_{iso}$ | G/L | % | $\delta_{iso}$ | $\Delta\delta_{iso}$ | G/L |
| SS $NaAlO_2$ | 16.5 | 175.8 | 0.57 | 0.30 | 15.5 | 137.4 | 0.60 | 0.08 | 68.0 | 127.7 | 1.57 | 0.00 |
| LS $NaAlO_2$ | 17.7 | 175.8 | 0.57 | 0.25 | 15.2 | 137.4 | 0.60 | 0.12 | 67.1 | 127.7 | 1.69 | 0.00 |
| SS AlAcac | 17.3 | 175.8 | 0.57 | 0.30 | 16.3 | 137.4 | 0.67 | 0.17 | 66.4 | 127.6 | 1.40 | 0.00 |
| LS AlAcac | 16.9 | 175.8 | 0.67 | 0.18 | 19.5 | 137.4 | 0.78 | 0.00 | 63.6 | 127.7 | 1.65 | 0.00 |

**Table S10**. $^{27}Al$ NMR parameters extracted from the simulations using second-order quadrupolar broadened models: Al(1)/(Al(1)+Al(2)) relative population (%), isotropic chemical shift ($\delta_{iso}$, ppm), distribution of isotropic chemical shift ($\Delta\delta_{iso}$, ppm), nuclear quadrupolar coupling constant ($C_Q$, MHz) and quadrupolar asymmetry parameter ($\eta_Q$). "SS" stands for "small scale" and "LS" for "large scale". * "n=0" spinning sideband of the satellite transition and/or impurity, amount is calculated from the total signal area.

| $^{27}Al$ NMR | Al(1) | | | | | Al(2) | | | | | Impurity/ssb | | |
|---|---|---|---|---|---|---|---|---|---|---|---|---|---|
| Compound | Al1/Al2 | $\delta_{iso}$ | $\Delta\delta_{iso}$ | $C_Q$ | $\eta_Q$ | Al1/Al2 | $\delta_{iso}$ | $\Delta\delta_{iso}$ | $C_Q$ | $\eta_Q$ | %* | $\delta_{iso}$ | $\Delta\delta_{iso}$ |
| SS $NaAlO_2$ | 57.3 | 8.3 | 2.42 | 4.86 | 0.64 | 42.7 | 8.6 | 1.26 | 8.61 | 0.52 | 1.2 | 13.5 | 3.83 |
| LS $NaAlO_2$ | 56.5 | 8.3 | 2.61 | 4.87 | 0.65 | 43.5 | 9.1 | 1.22 | 8.81 | 0.52 | 8.2 | 11.5 | 5.01 |
| SS AlAcac | 57.9 | 8.3 | 2.44 | 4.85 | 0.65 | 42.2 | 8.4 | 1.13 | 8.62 | 0.53 | 2.1 | 12.6 | 5.38 |
| LS AlAcac | 48.3 | 8.7 | 2.50 | 4.96 | 0.62 | 51.7 | 7.9 | 2.53 | 7.74 | 0.60 | 2.7 | 11.9 | 5.21 |

# Chapter 10 Shaping of MIL-120(Al)-AP

The MOF sample was shaped into spheres by extrusion followed by spheronization: at first, the MOF was mixed with the desired amount of binder in a mortar. After that, a paste was formed by adding to the MOF/binder powder a judicious amount of solvent until a favorable rheology is obtained. The paste was extruded using an extrusion apparatus (MULTILAB 806076) through a die with a nozzle diameter of 3 mm. The speed of extrusion was maintained at 20 rpm until all the paste was transformed into pellets. The attained pellets were then fed to the spheronizer with the speed set to 250 rpm for 10 minutes to obtain spheres (diameter around 3 mm). The spheres were dried under vacuum for 12 hours.

## 10.1 Shaping MIL-120(Al)-AP with 10% Bentonite

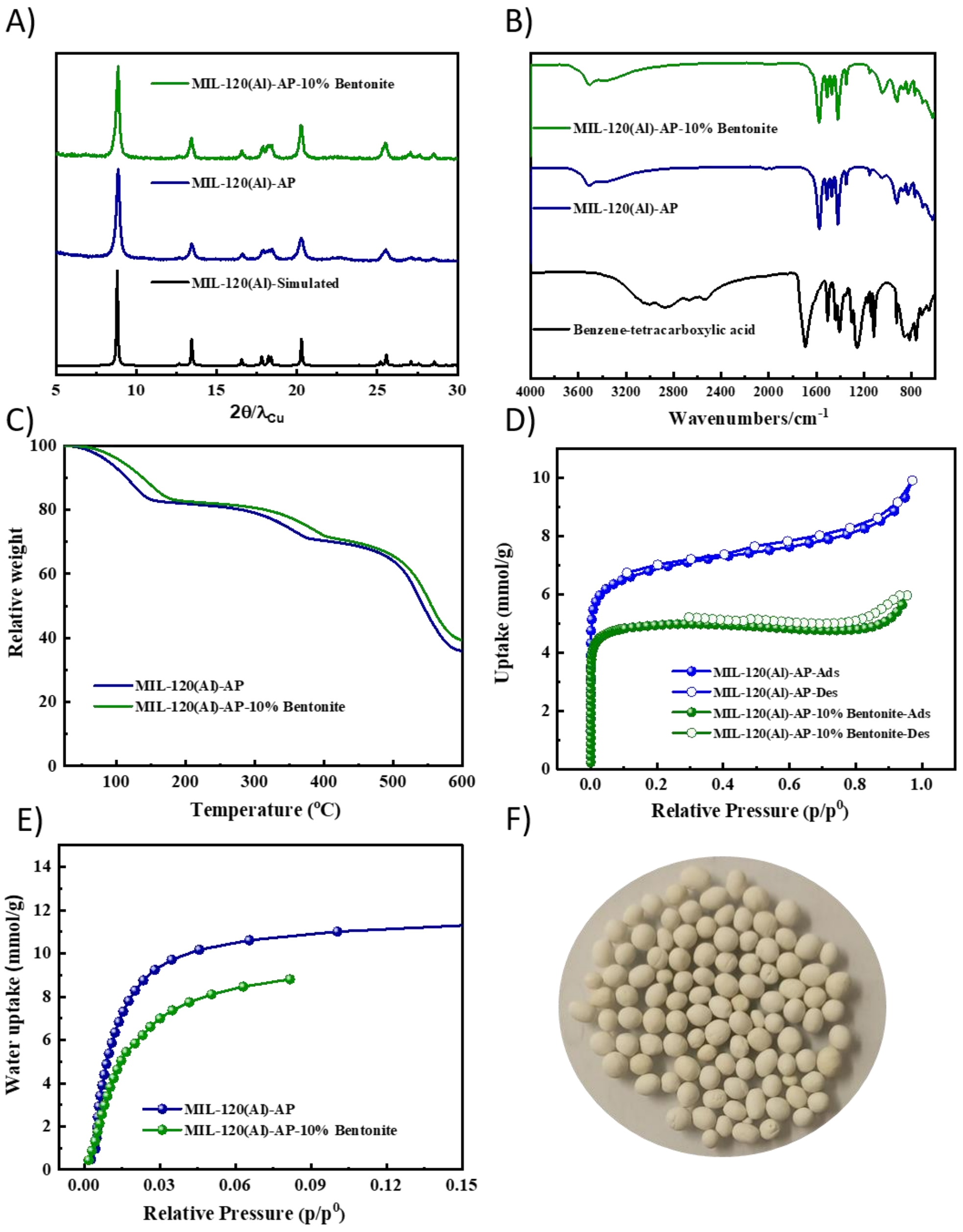


**Figure S25.** Comparison of the MIL-120(Al)-AP and MIL-120(Al)-AP with 10% Bentonite. A) PXRD patterns (CuKα radiation (λ = 1.5418 Å)). B) FT-IR spectra. C) TGA curves in $O_2$. D) $N_2$ sorption isotherms at 77 K (adsorption in filled symbols and desorption in empty symbols). E) Water adsorption isotherms at 298 K, F) Photograph of the MIL-120(Al)-AP beads with 3-5 mm diameter.

## 10.2 Shaping MIL-120(Al)-AP with 10% Silica

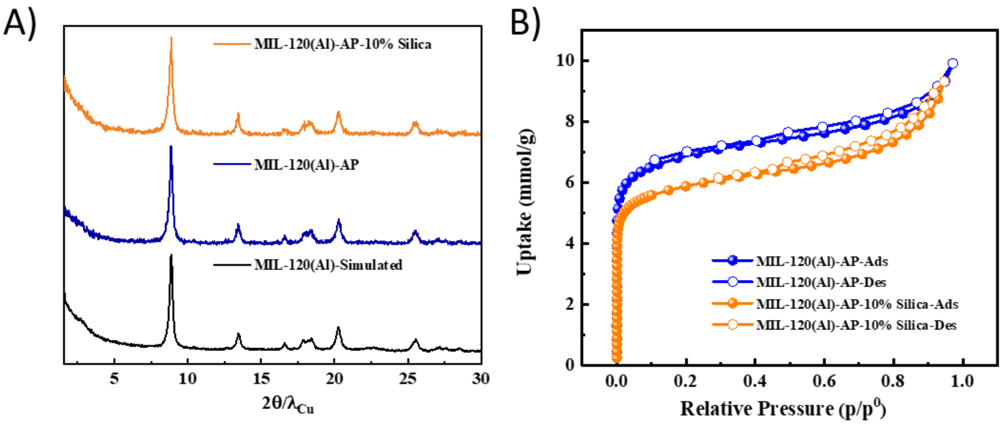


**Figure S26.** Comparison of the MIL-120(Al)-AP and MIL-120(Al)-AP with 10% Silica. A) PXRD patterns (CuKα radiation (λ = 1.5418 Å)). B) $N_2$ adsorption isotherms at 77 K K (adsorption in filled symbols and desorption in empty symbols).

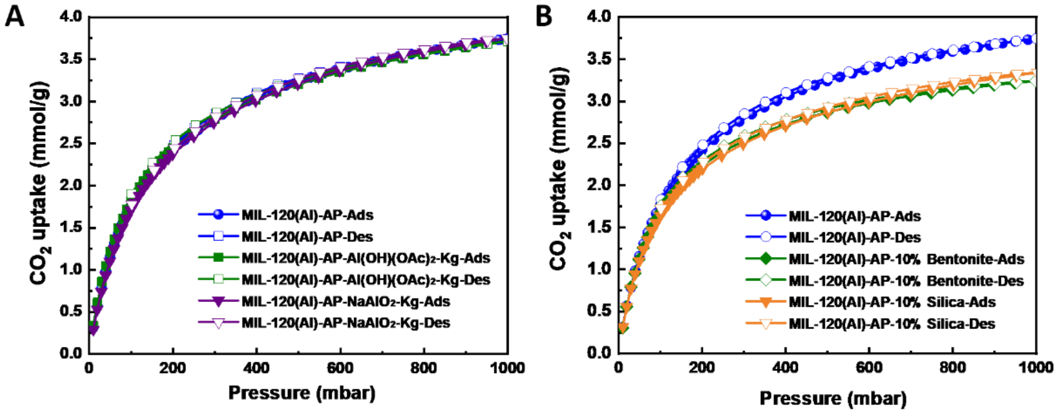


**Figure S27.** $CO_2$ sorption isotherms of MIL-120(Al)-AP at 298 K for A) samples prepared at different batch scales and using different Al precursors; and (B) for shaped samples with 10% Bentonite or Silica. Adsorption and desorption branches are represented in filled and empty symbols, respectively.

## Chapter 11 Breakthrough curve measurements

The experimental set-up is represented in Figure S28. It is a homemade device mainly composed of an adsorption column (5 cm height and 1 cm diameter (volume: 3.9 cm³)) containing the adsorbent, a gas supply system with four mass flow controllers [MFC] (one for helium, one for nitrogen, one for the $N_2/CO_2$ mixture and one for the $N_2/CO_2/H_2O$ mixture - Brooks Instrument: 0-10 NL $h^{-1}$), a pressure sensor (PS), a Pt100 temperature sensor, a vacuum pump (VP) and a mass spectrometer [MS] (InProcess Instruments - GAM 200 with a mass range from 1 to 200 amu) to analyze the gas composition at the outlet of the column. The mass spectrometer is calibrated before each set of measurements.

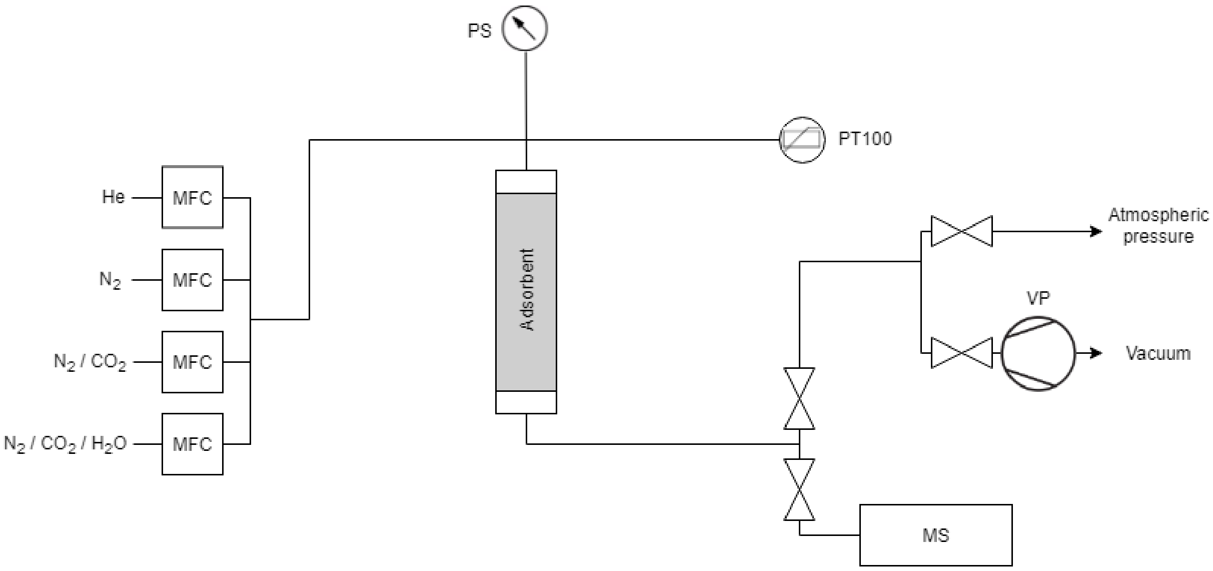


**Figure S28.** Experimental device for breakthrough curves measurements.

The experimental procedure for Run 1 was:

- Adsorbent activation at 50 °C for 12 hours under primary vacuum;
- Column pressurization: with helium at 30 °C (Flowrate setpoint: 2 NL $h^{-1}$);
- Adsorption measurement: $CO_2/N_2$ (0.15/0.85 molar fraction) at 30 °C (Flowrate setpoint: 1 NL $h^{-1}$)

The experimental procedure for the regenerability test was:

- Adsorbent activation at 50 °C for 12 hours under primary vacuum;
- Column pressurization: with nitrogen at 30 °C (Flowrate setpoint: 2 NL $h^{-1}$);

- Adsorption: Dry: $CO_2/N_2$ (0.15/0.85 molar fraction) or Humid: $CO_2/N_2/H_2O$ (0.15/0.85 molar fraction + water partial pressure of 0.03 bar) at 30 °C (Flowrate setpoint: 1 NL $h^{-1}$);
- Desorption: $N_2$ at 30 °C (Flowrate setpoint: 2 NL $h^{-1}$).

After this exposure to humid conditions, the sample was reactivated at 50 °C for 12 hours under vacuum. Then, Run 2 was carried out with a procedure similar to Run 1.

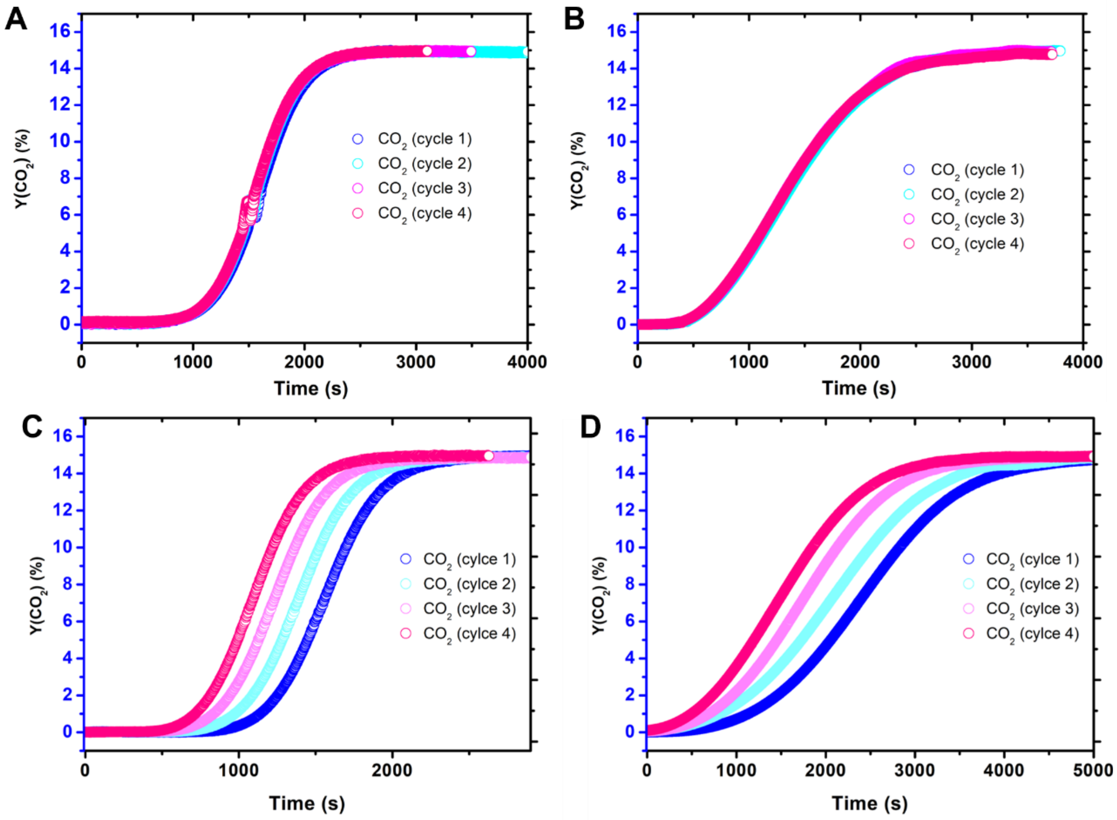


**Figure S29**. (A) & (C) $CO_2$ breakthrough curves for MIL-120(Al)-AP beads with 10% Si, and (B) & (D) 10% bentonite, obtained after four back-to-back cycles measurements under dry (upper, A & B) and wet conditions (without heating between the cycles) (lower, C & D).

**Table S11**. $CO_2$ adsorbed amounts after back-to-back cycling at 30 °C under dry conditions.

| | Cycle 1 | Cycle 2 | Cycle 3 | Cycle 4 |
|---|---|---|---|---|
| MIL-120(Al)-AP-10%Bentonite | 1.88 mmol/g | 1.84 mmol/g | 1.86 mmol/g | 1.85 mmol/g |
| MIL-120(Al)-AP-10%Silica | 1.65 mmol/g | 1.67 mmol/g | 1.66 mmol/g | 1.63 mmol/g |

## Chapter 12 *In situ* IR studies

IR experiments were carried out simulating *operando* conditions of post-combustion processes, using a weight hourly space velocity (WHSV) of 6 $Nm^3$ $h^{-1}$ $kg^{-1}$. Thus, thin pellets were prepared from powdered samples in order to perform IR analysis with a constant total flow of 25 mL $min^{-1}$. Pure Ar was flowed for the activation of the sample, from room temperature (RT) up to 300 °C, continuously monitoring the system *versus* time, to measure water and other species desorption.

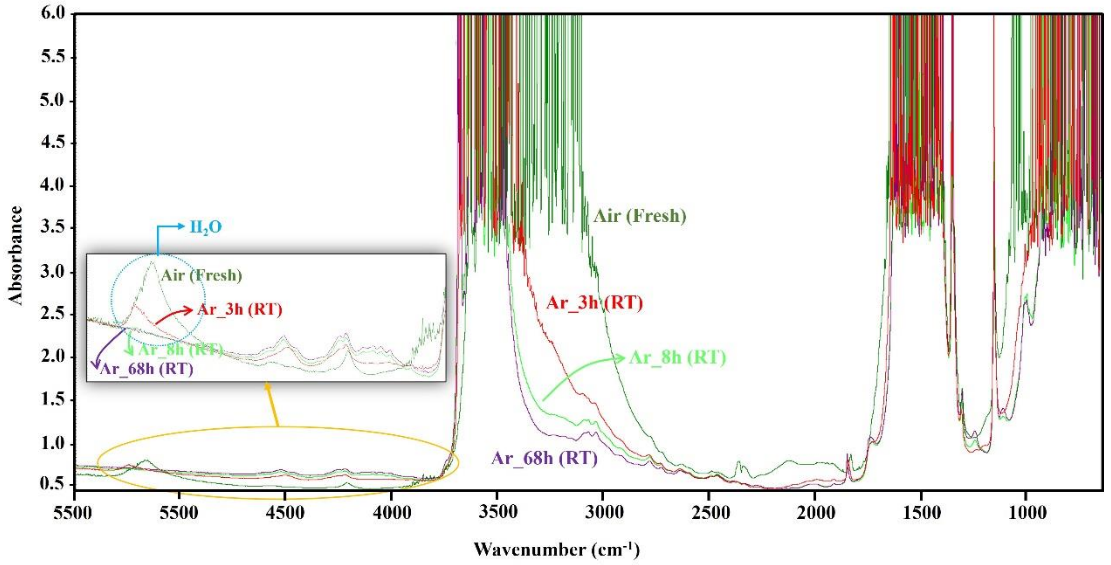


**Figure S30.** IR spectra of MIL-120(Al)-AP recorded in ambient air followed by room temperature activation (up to 68 hours) in Ar flow (WHSV 6 $Nm^3$ $h^{-1}$ $kg^{-1}$)). The decrease in intensity of the characteristic $(\nu+\delta)H_2O$ combination band of water (5170 $cm^{-1}$, highlighted in the insert) with activation time, shows that the latter was completely removed after 8 hours under stream at room temperature.

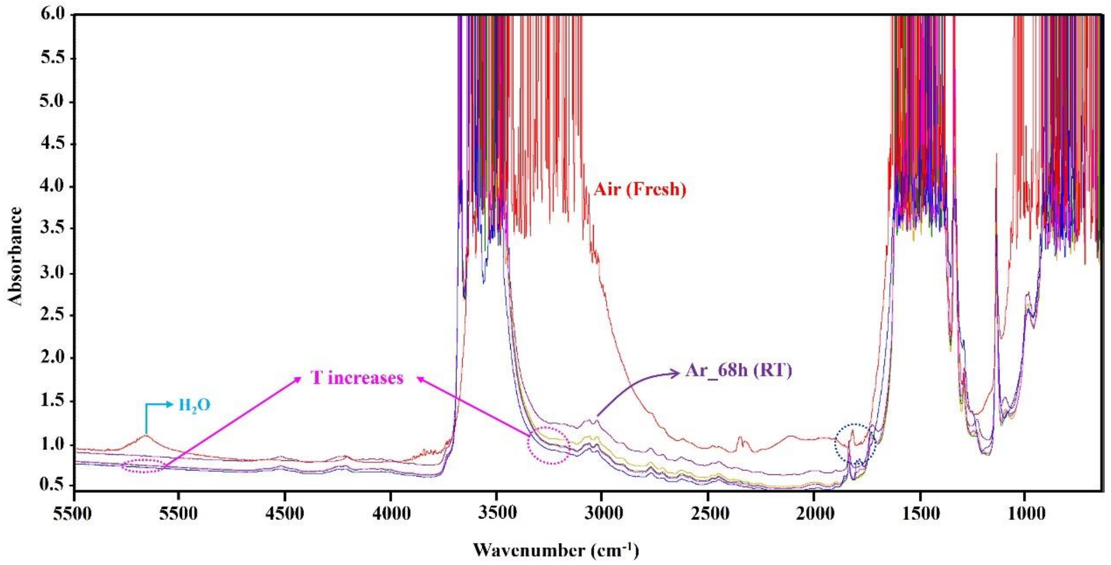


**Figure S31.** IR spectra of MIL-120(Al)-AP at different temperatures (50 – 100 – 150 – 200 – 300 °C) in Ar flow (WHSV 6 $Nm^3\ h^{-1}\ kg^{-1}$), after 68 hours activation in Ar flow at room temperature, confirming that the totally of the adsorbed water is removed without thermal activation.

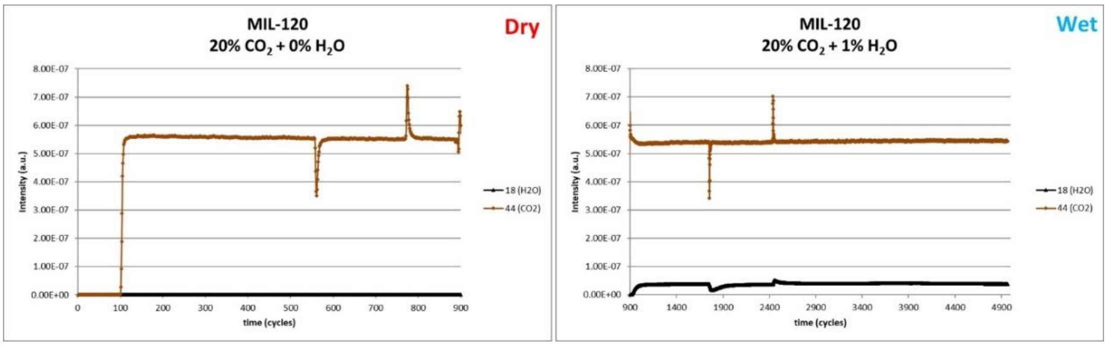


**Figure S32.** Mass Spectrometric (MS) signals of $CO_2$ (m/z=44) and $H_2O$ (m/z=18) recorded during $CO_2$ ads/desorption in dry & wet conditions at room temperature in Ar flow. The dry flow contains 20% of $CO_2$ in Ar flow(WHSV 6 $Nm^3\ h^{-1}\ kg^{-1}$); the wet flow consists of 20% $CO_2$ and 1% $H_2O$ in Ar flow (WHSV 6 $Nm^3\ h^{-1}\ kg^{-1}$).

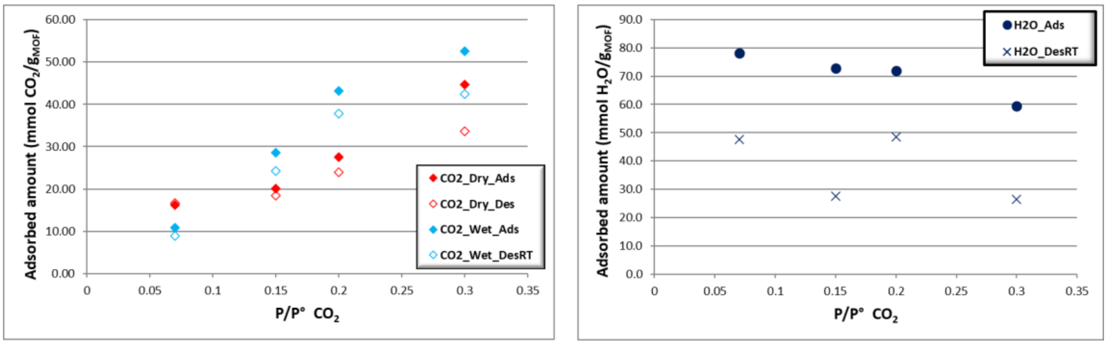


**Figure S33.** $CO_2$ and $H_2O$ uptakes in MIL-120(Al)-AP determined from MS signals. Dry $CO_2$: 7, 15, 20 and 30% $CO_2$ in Ar flow (WHSV 6 $Nm^3$ $h^{-1}$ $kg^{-1}$) at RT; Wet $CO_2$: 7, 15, 20 and 30% $CO_2$ + 1% $H_2O$ in Ar flow at room temperature.

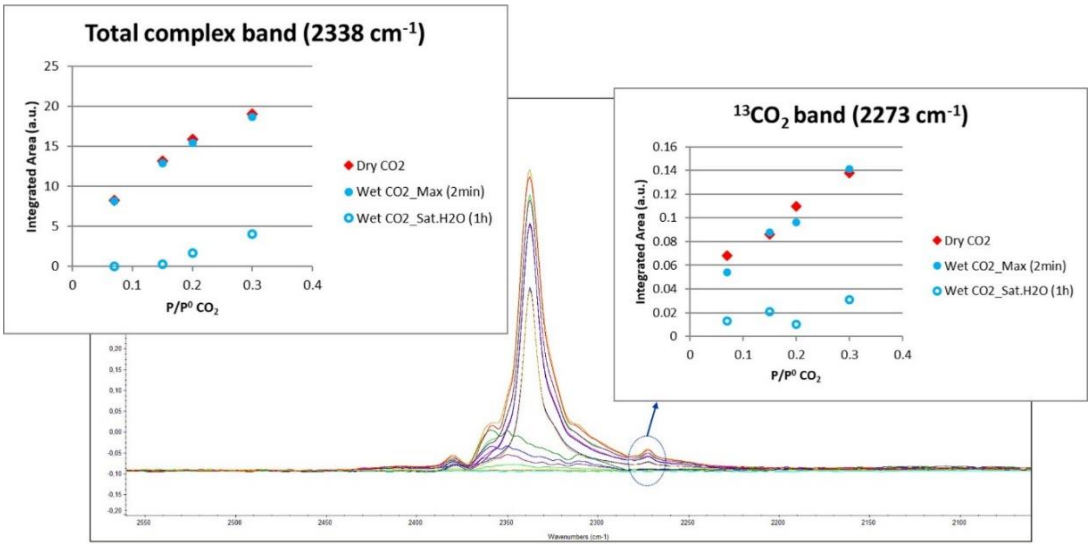


**Figure S34**. IR spectra of $CO_2$ adsorbed on MIL-120(Al)AP recorded during $CO_2$ ads/desorption (Dry & Wet) in Ar flow (25 ml·min$^{-1}$). Inserts: relative $CO_2$ uptake calculated from the integrated intensities of the ν3($CO_2$) bands. Dry $CO_2$: 7, 15, 20 and 30% $CO_2$ in Ar flow at room temperature; wet $CO_2$: 7, 15, 20 and 30% $CO_2$ + 1% $H_2O$ in Ar flow at room temperature. Both $^{12}CO_2$ and $^{13}CO_2$ isotopes have been taken into account.

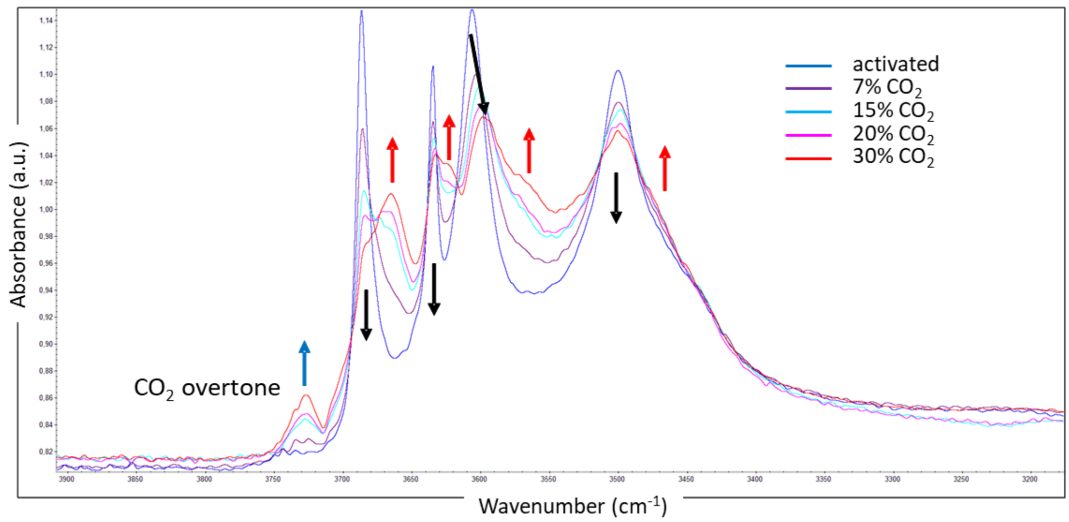


**Figure S35**. IR spectra of the OH groups (ν(OH) range) of the activated sample and upon contact with a dry flow (25 mL min$^{-1}$) containing 7, 15, 20 and 30% $CO_2$ in Ar at RT. Upon $CO_2$ concentration increase, OH groups perturbations become more and more evident.

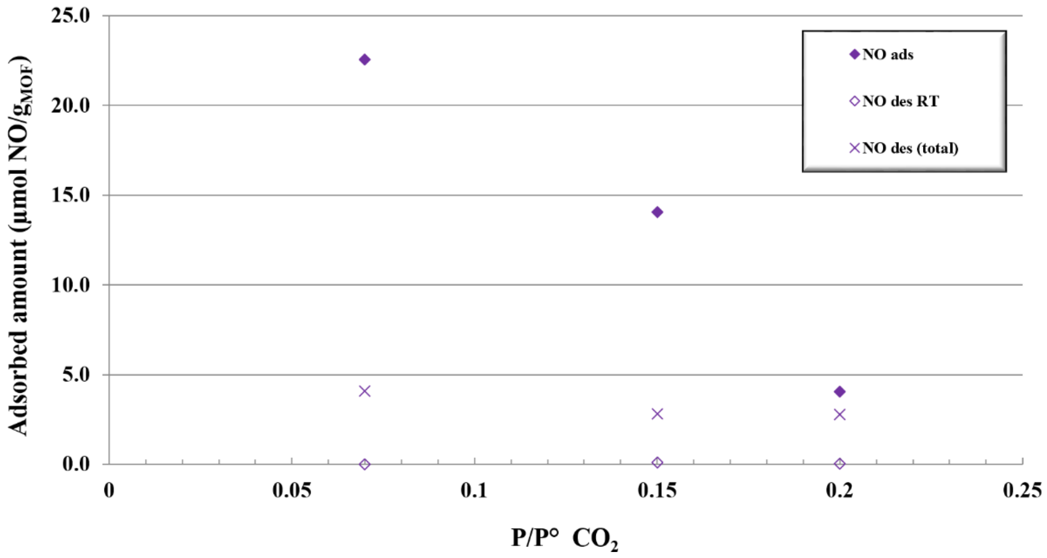


**Figure S36.** NO adsorption and desorption for MIL-120 under the complete flow, quantified by *operando* IR. The NO uptake is evaluated for 20% $CO_2$ and 1% $H_2O$ feed.

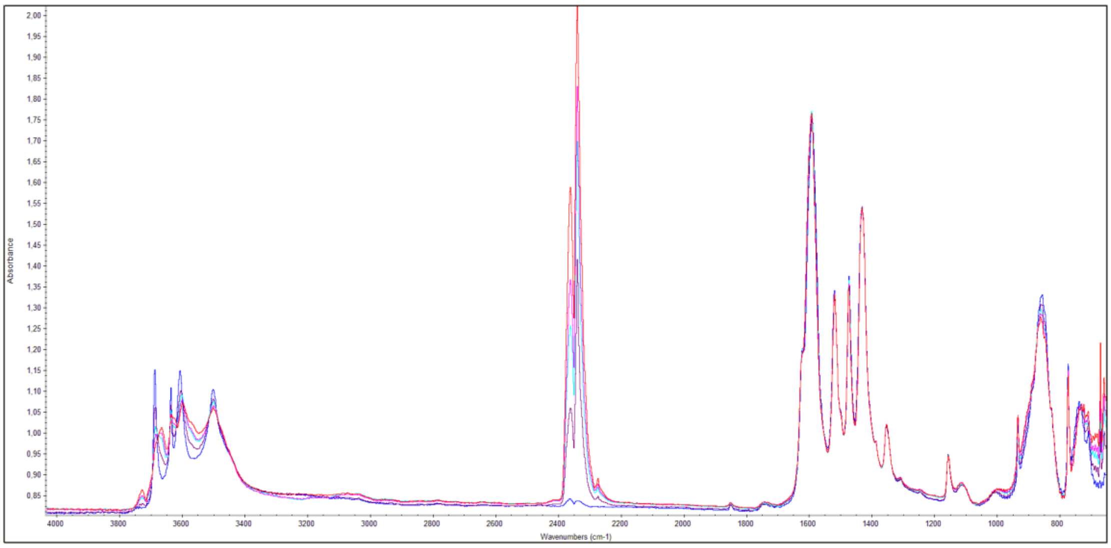


**Figure S37.** IR spectra of the MIL-120 sample deposed on a Si plate and submitted to the complete reaction flow with 20% of $CO_2$. Only changes due to the adsorption of $CO_2$ and water are observed (spectral regions 3800 – 2200 $cm^{-1}$ and below 1000 $cm^{-1}$), together with a very weak band above 1850 $cm^{-1}$, due to NO adsorption. No other band increase or modifications are observed in the region 2000 – 1000 $cm^{-1}$, where NOx and SOx impurities adsorption are expected, nor in the skeletal bands of the solid.

## Chapter 13  Techno-economic analysis of MIL-120(Al)-AP production costs

The simplified process block diagram of the proposed industrial-scale production of MIL-120(Al)-AP is represented in Figure S37. The major equipment parts considered for the techno-economic analysis were silo/storage tanks for each reactant and the final product, a batch stirred reactor with a propeller agitator, a plate-and-frame filter to filter and wash the solid material produced in the reactor, a rotary dryer to dry the washed solid and a conveyor belt to transport the dried MIL-120 to its storage silo.

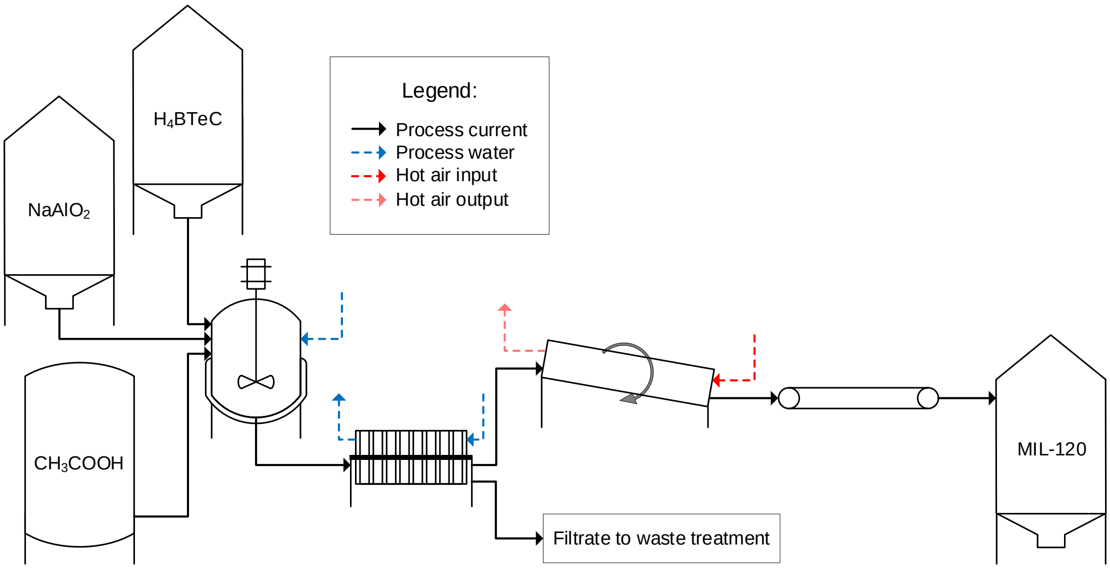


**Figure S38**. Process block diagram for the industrial-scale production of MIL-120.

The process equipment was designed and dimensioned based on common chemical engineering heuristics, the properties of the compounds, the process conditions and targeted production (considering the reaction yield and STY), and 260 days of work per year. The cost of each equipment was obtained from online databases indexed for the years 2014[33] and 2002[34]. These costs were updated to the years 2019 and 2022 based on the Chemical Engineering Plant Cost Index (CEPCI), according to:

$$\mathrm{Cost}_{\mathrm{Year\ 2}} = \mathrm{Cost}_{\mathrm{Year\ 1}} \times \frac{\mathrm{CEPCI}_{\mathrm{Year\ 2}}}{\mathrm{CEPCI}_{\mathrm{Year\ 1}}}$$

The obtained equipment costs with the relevant specifications used to obtain each cost are shown in Table S12. We note that all silos/storage tanks were dimensioned for 30 days of storage and the volume needed was over-dimensioned by 20%.

**Table S12**. Major equipment cost.

| **Major equipment** | **Relevant specifications** | **Cost 2019 (USD, \$)** | **Cost 2022 (USD, \$)** | **Source** |
|---|---|---|---|---|
| Reactor and agitator | • $V_{reactor}$ = 45 m$^3$<br>• $P_{agitator}$ = 192 kW<br>• $p_{atm}$<br>• Stainless steel (SS) 316 | \$716 494.71 | \$931 135.02 | [33] |
| Storage: $H_4BTeC$ | • $\rho_{bulk}$ = 560 kg m$^{-3}$ [[35]]<br>• $V_{silo}$ = 168 m$^3$<br>• SS-304 | \$168 580.58 | \$219 082.26 | [33] |
| Storage: $NaAlO_2$ | • $\rho_{bulk}$ = 977 kg m$^{-3}$ [[36]]<br>• $V_{silo}$ = 124 m$^3$<br>• SS-304 | \$141 991.27 | \$184 527.59 | [33] |
| Storage: $CH_3COOH$ | • $\rho$ = 1049 kg m$^{-3}$ [[37]]<br>• $V_{tank}$ = 169 m$^3$<br>• SS-316 | \$222 522.01 | \$289 182.92 | [33] |
| Storage: MIL-120 | • $\rho_{bulk}$ = 600 kg m$^{-3}$ [a]<br>• $V_{silo}$ = 231 m$^3$<br>• SS-304 | \$191 682.77 | \$249 105.17 | [33] |
| Plate-and-frame filter | • $\delta_{cake}$ = 0.05 m<br>• $L_{plate}$ = $W_{plate}$ = 1.5 m<br>• $A_{filtration}$ = 48 m$^2$<br>• SS-CF-8M | \$144 010.68 | \$187 151.96 | [34] |
| Rotary dryer | • $D$ = 0.46 m<br>• $L$ = 1.83 m<br>• $A_{drying}$ = 4.65 m$^2$<br>• $P_{rotation}$ = 3.73 kW<br>• $P_{dry}$ = 1.36 MW<br>• SS-316 | \$204 732.88 | \$266 064.71 | [34] |
| Conveyer belt | • $W$ = 0.4 m<br>• $L$ = 50 m<br>• $P$ = 0.59 kW | \$91 684.11 | \$119 149.92 | [34] |
| **Total base equipment cost** | | \$1 881 699.00 | \$2 445 399.57 | – |

[a] This is a common average value for bulk density of powdered solids. The actual value will depend on the hydration state and particle size.

The fixed investment is composed of direct and indirect costs. A risk provision should also be considered. The direct costs can be estimated based on the total base equipment cost and the indirect costs can be estimated based on the direct costs. Using the total base equipment cost presented in Table S13 and assuming some common cost factors

heuristics for chemical engineering process[34] we estimated the total fixed investment according to the equation:

$$\text{Fixed investment} = [\text{Cost}_{\text{direct}} + \text{Cost}_{\text{indirect}}](1 + f'')$$

$$= \left[\text{Cost}_{\text{base equip.}} \times \sum_{n=1}^{9} f_n + \text{Cost}_{\text{direct}}(f_1' + f_2')\right](1 + f'')$$

with $f_n$ corresponding to factors applied over the total base equipment cost in $n$ different cost segments, $f_1'$ and $f_2'$ to factors applied over the direct cost for the project cost and control, and for the building's cost, respectively, and $f''$ to a factor to be applied over the direct costs for the risk provision.

The total investment is composed of not only the fixed investment, but also the working capital and the interim interests paid over the bank loan. The working capital can be calculated as a percentage over the fixed investment. The interim interests take into account what is the percentage of the total investment (without interest) that will be obtained by bank loan (60%), the interest rate (5%) and 24 months for the construction of the plant with only interim interest payment during this period.

The detailed calculations are shown in the Table S13.

**Table S13.** Segmentation of different costs used to estimate the total investment.

| Cost segment | Factor ($f_n$) | 2019 | 2022 |
|---|---|---|---|
| Base equipment (*cf.* Table S12) | 1 | $1 881 699.00 | $2 445 399.57 |
| Assembly of base equipment | 0.45 | $846 764.55 | $1 100 429.80 |
| Tubes | 0.3 | $564 509.70 | $733 619.87 |
| Control | 0.3 | $564 509.70 | $733 619.87 |
| Buildings | 0.15 | $282 254.85 | $366 809.93 |
| Land and preparation | 0.15 | $282 254.85 | $366 809.93 |
| Electronic installations | 0.15 | $282 254.85 | $366 809.93 |
| Thermal isolation | 0.1 | $188 169.90 | $244 539.96 |
| Utilities and services: | 0.112: | $210 750.29 | $273 884.75 |
| • *Generated vapour* | • *0.03* | *$56 450.97* | *$73 361.99* |
| • *Vapour distribution* | • *0.01* | *$18 816.99* | *$24 454.00* |
| • *Substation electricity* | • *0.013* | *$24 462.09* | *$31 790.19* |
| • *Electricity distribution* | • *0.01* | *$18 816.99* | *$24 454.00* |
| • *Water for general use* | • *0.018* | *$33 870.58* | *$44 017.19* |
| • *Water distribution* | • *0.008* | *$15 053.59* | *$19 563.20* |
| • *Air of instrument* | • *0.01* | *$18 816.99* | *$24 454.00* |
| • *Effluent treatment* | • *0.013* | *$24 462.09* | *$31 790.19* |
| **Total direct costs** | | **$5 103 167.70** | **$6 631 923.62** |
| | | | |
| **Cost segment** | **Factor ($f'_n$)** | **2019** | **2022** |
| Project cost and control | 0.3 | $1 530 950.31 | $1 989 577.09 |
| Building's cost | 0.3 | $1 530 950.31 | $1 989 577.09 |
| **Total indirect costs** | | **$3 061 900.62** | **$3 979 154.17** |
| | | | |
| **Cost segment** | **Factor ($f''$)** | **2019** | **2022** |
| Provision for unforseen events | 0.15 | $765 475.15 | $994 788.54 |
| | | | |
| **Total fixed investment** | | **$8 930 543.47** | **$11 605 866.34** |
| | | | |
| | **Factor** | **2019** | **2022** |
| Working capital | 0.2 | $1 786 108.69 | $1 786 108.69 |
| | | | |
| **Total investment without interest** | | **$10 716 652.17** | **$13 391 975.04** |
| | | | |
| **Interim interests** | | **$446 527.17** | **$557 998.96** |
| | | | |
| **Total investment with interest** | | **$11 163 179.34** | **$13 949 974.00** |

The production costs are composed of the manufacturing costs and the general expenses, with the manufacturing costs being further divided into direct, indirect and fixed costs. For the direct costs of the manufacturing costs, considering the yearly production and

the yield, we calculated the total raw materials cost with the prices of $H_4BTeC$, acetic acid ($CH_3COOH$) and sodium aluminate ($NaAlO_2$), of 1 \$/kg,[38] 0.79 \$/kg (estimated from the total global imports of acetic acid, as listed on the *UN Comtrade Database*,[39] commodity code HS 291521) and 0.58 \$/kg (taken as the average value,[40] and converted to \$/kg), respectively. From the required energy consumption, we calculated the utilities costs as solely based on the energy costs considering an energy tariff of 0.0506 and 0.1472 €/kWh, for 2019 and 2022, respectively, as listed for the European Union in the *Eurostat Data Browser*[41] for an energy consumption in the range 2000-19999 MWh (band ID). For the operating labour cost, we estimated a total of 25 persons operating the plant in 5 shifts, each with an average annual salary of *ca*. \$22440 and \$25576, for 2019 and 2023, respectively (converted from £20535,[42] and retro-adjusted for inflation for 2019). The remaining segments that contribute to the total direct manufacturing costs (Table S14) were estimated using cost factors.[34] The depreciation costs were estimated from the fixed investment using the common depreciation rates and the other factors of the fixed costs were estimated using common chemical engineering factors (Table S14). The financial charges were estimated from the capital borrowed from the bank and the remaining of the general expenses were estimated using cost factors estimation (Table S14).[34] The total production costs were then divided by the yearly production of MIL-120 (1000 ton) to give the final production cost in \$/kg (Table S14). We note that all currency values were converted to USD (\$) using the average exchange rates reported for each year.

**Table S14.** Segmentation of different costs used to estimate the production costs.

| Cost segment | Factor | 2019 | 2022 |
|---|---|---|---|
| Raw materials | – | $2 024 024.15 | $2 718 689.22 |
| Operating labour | – | $690 043.97 | $786 472.53 |
| Operating supervision | 0.15 (over operating labour) | $103 506.60 | $117 970.88 |
| Utilities | – | $283 900.47 | $777 145.54 |
| Maintenance and repairs | 0.07 (over total fixed investment) | $750 165.65 | $937 438.25 |
| Operating supplies | 0.15 (over maintenance and repairs) | $112 524.85 | $140 615.74 |
| Laboratory charges | 0.15 (over operating labour) | $103 506.60 | $117 970.88 |
| Royalties | 0.04 (over total production costs) | $394 412.74 | $517 619.00 |
| **Direct costs** | | **$4 462 085.02** | **$6 113 922.04** |
| | | | |
| **Cost segment** | **Factor** | **2019** | **2022** |
| Indirect costs | 0.6 (over total labour and maintenance costs) | $926 229.73 | $1 105 129.00 |
| | | | |
| **Cost segment** | **Factor** | **2019** | **2022** |
| Depreciation | – | $1 234 289.17 | $1 604 045.17 |
| Taxes | 0.02 (over total fixed investment) | $214 333.04 | $267 839.50 |
| Insurance | 0.01 (over total fixed investment) | $107 166.52 | $133 919.75 |
| Rent | 0.01 (over total fixed investment) | $107 166.52 | $133 919.75 |
| **Fixed costs** | | $1 662 955.26 | $2 139 724.17 |
| | | | |
| **Manufactoring costs** | | **$7 051 270.01** | **$9 358 775.22** |
| | | | |
| **Cost segment** | **Factor** | **2019** | **2022** |
| Administration | 0.6 | $476 130.34 | $542 666.05 |
| Marketing, sales and distribution | 0.2 | $1 972 063.69 | $2 588 094.99 |
| R&D | 0.05 (over sales) – Not considered | – | – |
| Financial charges | – | $360 854.39 | $450 938.68 |
| **General expenses** | | **$2 809 048.42** | **$3 581 699.72** |
| | | | |
| **Production cost** | | **$9 860 318.43** | **$12 940 474.93** |
| **Production cost per kg** | | **$9.86** | **$12.94** |

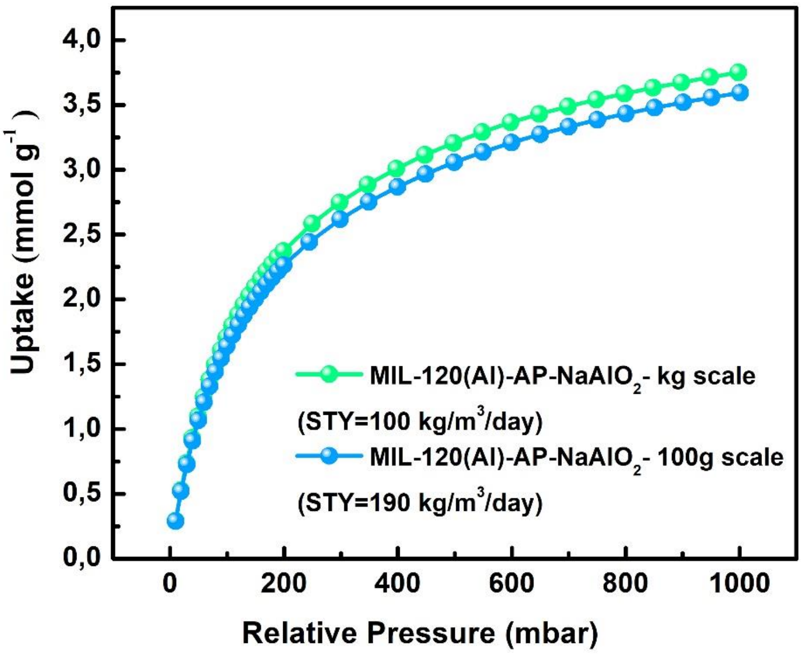


**Figure S39.** $CO_2$ adsorption isotherms at 298 K on different batches prepared with different synthesis durations (green = 24h; blue = 13h), showing no significant decrease on the $CO_2$ uptake performance.

# Chapter 14 References


[1] R. Span, W. Wagner, *Journal of Physical and Chemical Reference Data* **1996**, 25, 1509.

[2] R. Span, E. W. Lemmon, R. T. Jacobsen, W. Wagner, A. Yokozeki, *Journal of Physical and Chemical Reference Data* **2000**, 29, 1361.

[3] a)N. Heymans, S. Vaesen, G. D. Weireld, *Microporous and Mesoporous Materials* **2012**, 154, 93; b)G. D. Weireld, M. Frère, R. Jadot, *Measurement Science and Technology* **1999**, 10, 117.

[4] T. Lesage, C. Verrier, P. Bazin, J. Saussey, M. Daturi, *Physical Chemistry Chemical Physics* **2003**, 5, 4435.

[5] C. Volkringer, T. Loiseau, M. Haouas, F. Taulelle, D. Popov, M. Burghammer, C. Riekel, C. Zlotea, F. Cuevas, M. Latroche, *Chemistry of Materials* **2009**, 21, 5783.

[6] A. L. Myers, J. M. Prausnitz, *AIChE Journal* **1965**, 11, 121.

[7] I. Langmuir, *Journal of the American Chemical society* **1918**, 40, 1361.

[8] P. Billemont, N. Heymans, P. Normand, G. De Weireld, *Adsorption* **2017**, 23, 225.

[9] N. Tolazzi, E. Steffani, E. Barbosa-Coutinho, J. B. S. Júnior, J. C. Pinto, M. Schwaab, *Chemical Engineering Research and Design* **2018**, 138, 144.

[10] a)J. Zhang, R. Singh, P. A. Webley, *Microporous and Mesoporous Materials* **2008**, 111, 478; b)J. Shang, G. Li, R. Singh, Q. Gu, K. M. Nairn, T. J. Bastow, N. Medhekar, C. M. Doherty, A. J. Hill, J. Z. Liu, *Journal of the American Chemical Society* **2012**, 134, 19246; c)Y. Guo, T. Sun, Y. Gu, X. Liu, Q. Ke, X. Wei, S. Wang, *Chemistry–An Asian Journal* **2018**, 13, 3222.

[11] a)F. Brandani, D. Ruthven, C. G. Coe, *Industrial & Engineering Chemistry Research* **2003**, 42, 1451; b)T.-H. Bae, M. R. Hudson, J. A. Mason, W. L. Queen, J. J. Dutton, K. Sumida, K. J. Micklash, S. S. Kaye, C. M. Brown, J. R. Long, *Energy & Environmental Science* **2013**, 6, 128.

[12] a)P. J. E. Harlick, F. H. Tezel, *Microporous and Mesoporous Materials* **2004**, 76, 71; b)P. Nugent, Y. Belmabkhout, S. D. Burd, A. J. Cairns, R. Luebke, K. Forrest, T. Pham, S. Ma, B. Space, L. Wojtas, *Nature* **2013**, 495, 80.

[13] J.-B. Lin, T. T. Nguyen, R. Vaidhyanathan, J. Burner, J. M. Taylor, H. Durekova, F. Akhtar, R. K. Mah, O. Ghaffari-Nik, S. Marx, *Science* **2021**, 374, 1464.

[14] A. Masala, J. G. Vitillo, G. Mondino, C. A. Grande, R. Blom, M. Manzoli, M. Marshall, S. Bordiga, *ACS applied materials & interfaces* **2017**, 9, 455.

[15] G. Ashiotis, A. Deschildre, Z. Nawaz, J. P. Wright, D. Karkoulis, F. E. Picca, J. Kieffer, *Journal of Applied Crystallography* **2015**, 48, 510.

[16] V. Dyadkin, Pattison, P., Dmitriev, V., Chernyshov, D., *Journal of Synchrotron Radiation* **2016**, 23.

[17] J. Rodríguez-Carvajal, *Physica B: Condensed Matter* **1993**, 192, 55.

[18] W. Kraus, G. Nolze, *Journal of Applied Crystallography* **1996**, 29, 301.

[19] Vincent Favre-Nicolin, R. Cerny, *Journal of Applied Crystallography* **2002**, 35, 734.

[20] G. Kresse, J. Furthmüller, *Physical Review B* **1996**, 54, 11169.

[21] P. E. Blöchl, *Physical Review B* **1994**, 50, 17953.

[22] J. P. Perdew, K. Burke, M. Ernzerhof, *Physical review letters* **1996**, 77, 3865.

[23] S. Grimme, J. Antony, S. Ehrlich, H. Krieg, *The Journal of Chemical Physics* **2010**, 132.
[24] H. J. Monkhorst, J. D. Pack, *Physical Review B* **1976**, 13, 5188.
[25] Q. Yang, C. Zhong, *The Journal of Physical Chemistry B* **2006**, 110, 17776.
[26] A. K. Rappe, C. J. Casewit, K. S. Colwell, W. A. Goddard, III, W. M. Skiff, *Journal of the American Chemical Society* **1992**, 114, 10024.
[27] J. G. Harris, K. H. Yung, *The Journal of Physical Chemistry* **1995**, 99, 12021.
[28] J. J. Potoff, J. I. Siepmann, *AIChE Journal* **2001**, 47, 1676.
[29] H. A. Lorentz, *Annalen der Physik* **1881**, 248, 127.
[30] B. R. Svensson, C. E. Woodward, *Molecular Physics* **1988**, 64, 247.
[31] J.-P. Amoureux, C. Fernandez, S. Steuernagel, *Journal of Magnetic Resonance, Series A* **1996**, 123, 116.
[32] D. Massiot, F. Fayon, M. Capron, I. King, S. Le Calvé, B. Alonso, J.-O. Durand, B. Bujoli, Z. Gan, G. Hoatson, *Magnetic Resonance in Chemistry* **2002**, 40, 70.
[33] D. Milligan, J. Milligan., *Matches' engineering to chemical energy manufactoring metallurgical industries,,* **2014**.
[34] M. S. Peters, Klaus D. Timmerhaus, R. E. West., *Plant Design and Economics for Chemical Engineers*, New York: McGraw-Hill, **2003**.
[35] *Chemical Book, Pyromellitic acid*, **2023**.
[36] in *Anval Valves PVT LTD, Bulk Density Chart, (n.d.) 1–15*, (Ed: https://anval.net/Downloads/Bulk%20Density%20Chart.pdf).
[37] *Thermo Fisher Scientific Inc., Acetic acid, glacial,* Safety Data Sheet, **2021**.
[38] S. Khodabakhshi, S. Kiani, Y. Niu, A. O. White, W. Suwaileh, R. E. Palmer, A. R. Barron, E. Andreoli, *Carbon* **2021**, 171, 426.
[39] *U. Nations, UN Comtrade Database,* **2022**.
[40] F. Ye, B. He, C.-m. Tian, M. Zhao, J. Wang, X.-b. Han, D. J. Armaghani, *Tunnelling and Underground Space Technology* **2021**, 117, 104156.
[41] *Eurostat, Electricity prices components for non-household consumers - annual data (from 2007 onwards),* **2023**.
[42] in *The Stepstone Group, What is the average salary for Machine Operator in Europe?,* (Ed: https://www.totaljobs.com/jobs/machine-operator/in-europe).
